# The sirtuin family: from mechanisms to therapy

**DOI:** 10.1186/s43556-026-00631-0

**Published:** 2026-10-23

**Authors:** Jiaying Xiao, Yiling Li, Yuyao Xie, Yuan Chen, Qihan Liu, Hao Li, Rui Sun

**Affiliations:** 1 Department of Gastroenterology, Shengjing Hospital Affiliated to China Medical University, Shenyang, China; 2 Department of Gastroenterology, The First Affiliated Hospital of China Medical University, Shenyang, China; 3 Times Joyee (Liaoning) Information Co., Ltd, Shenyang, China; 4 Department of Urology, Urologic Surgery Centre, Xinqiao Hospital, Third Military Medical University (Army Medical University), Chongqing, China; 5 Department of Medical Genetics, China Medical University, Shenyang, China

**Keywords:** Sirtuins, NAD^+^, Metabolism, Immunity, Therapeutics

## Abstract

Sirtuins (SIRT1–SIRT7) are evolutionarily conserved NAD^+^-dependent deacylases that function as metabolic sensors, coupling cellular metabolic state to epigenetic, transcriptional and post-translational regulatory programs across distinct subcellular compartments. Despite two decades of research, isoform- and disease-specific aspects remain poorly integrated, limiting both conceptual clarity and therapeutic translation. This review synthesizes recent advances in sirtuin biology into a family-wide framework. Rather than functioning as isolated longevity factors, sirtuins operate as a distributed compartmentalized regulatory network in which nuclear SIRT1, SIRT6 and SIRT7, cytosolic SIRT2 and mitochondrial SIRT3, SIRT4 and SIRT5 collectively govern glucose and lipid metabolism, mitochondrial quality control, autophagy, inflammation and immune tolerance. This network operates across metabolic disorders, cardiovascular and neurodegenerative diseases, inflammatory conditions and cancer, where dysregulated sirtuin signalling influences disease development in a disease- and tissue-specific manner. Therapeutic strategies are evaluated, ranging from isoform-selective small-molecule modulators and NAD^+^-targeted interventions to emerging protein-degradation approaches, with isoform selectivity, off-target effects and the systemic consequences of NAD^+^ modulation identified as key translational barriers that have contributed to heterogeneous clinical outcomes. Viewing sirtuins as a context-sensitive compartmentalized network, not isolated targets, offers a coherent framework to guide mechanistic interrogation and the design of more precise sirtuin-based therapies, with priority areas including tissue-selective degradation and network-level biomarkers.

## Introduction

Sirtuins are NAD^+^-dependent deacylases. They link cellular energy status to epigenetic, metabolic and stress responses. The founding member, yeast Sir2, silences transcription and extends replicative lifespan under caloric restriction. Its mammalian homologues, SIRT1–SIRT7, are key regulators of ageing across eukaryotes [1].

Sir2 was subsequently identified as an NAD^+^-dependent histone deacetylase, directly linking cellular metabolism to epigenetic control [2, 3]. Sir2 overexpression extends replicative lifespan in yeast and promotes longevity in Caenorhabditis elegans and Drosophila melanogaster, effects closely associated with caloric restriction and NAD^+^ availability [4–6].

**Fig. 1:**
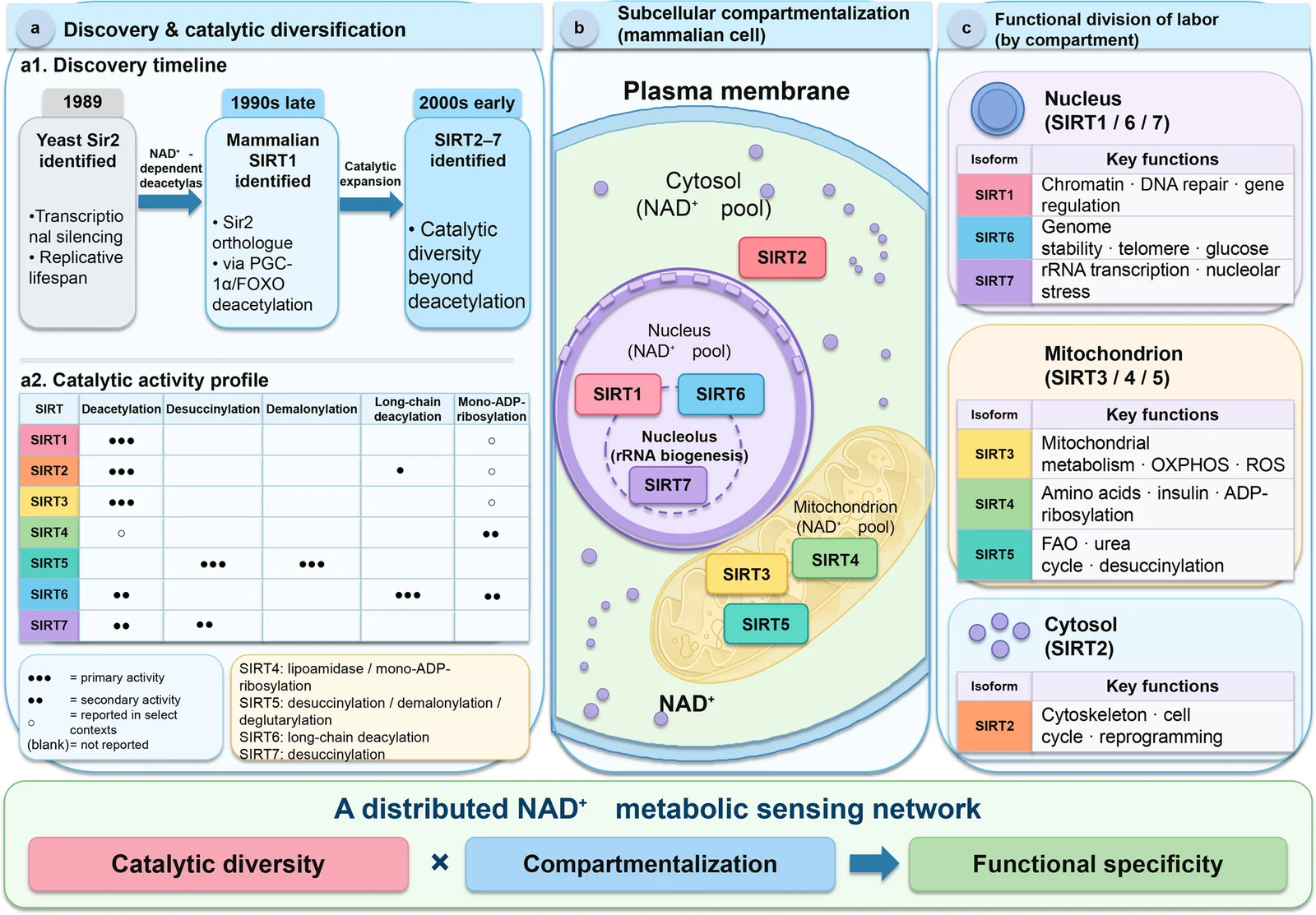
Discovery, diversification and compartmentalization of the sirtuin family. **a** Discovery and catalytic diversification. Yeast Sir2 was identified in 1989 as a transcriptional silencer and was subsequently shown to be an NAD^+^-dependent histone deacetylase; Sir2 overexpression extends replicative lifespan in yeast. Mammalian SIRT1, the closest orthologue of Sir2, was identified in the late 1990 s and regulates metabolic adaptation, stress resistance and genome maintenance through deacetylation of PGC-1α and FOXO. SIRT2–7 were identified in the early 2000 s, revealing catalytic diversity beyond deacetylation: SIRT5 catalyses desuccinylation and demalonylation, SIRT6 mediates long-chain deacylation, and select isoforms exhibit mono-ADP-ribosylation. **b** Subcellular compartmentalization. SIRT1, SIRT6 and SIRT7 are nuclear (SIRT7 is enriched in the nucleolus); SIRT3, SIRT4 and SIRT5 are mitochondrial; SIRT2 is predominantly cytosolic. **c** Functional division of labour by compartment. Nuclear sirtuins regulate chromatin remodelling, DNA repair and metabolic gene regulation; mitochondrial sirtuins control oxidative phosphorylation, fatty-acid oxidation, amino-acid metabolism and the urea cycle; cytosolic SIRT2 modulates cytoskeletal dynamics and cell-cycle progression. Catalytic diversity and compartmentalization jointly confer functional specificity, forming a distributed NAD^+^ metabolic sensing network

In mammals, seven homologues (SIRT1–SIRT7) were identified in the late 1990 s and early 2000 s (Fig. 1). Although they share a conserved catalytic core, they differ in localization and regulatory function. SIRT1, the closest orthologue of yeast Sir2, initially attracted considerable attention due to its roles in metabolic adaptation, stress resistance and genome maintenance through deacetylation of transcriptional regulators such as PGC-1α and FOXO family members [7–9]. However, it soon became clear that sirtuin biology extends beyond SIRT1 and beyond deacetylation alone. SIRT5 catalyses desuccinylation and demalonylation [10, 11], SIRT6 mediates long-chain deacylation [12], and select isoforms exhibit mono-ADP-ribosylation activity [13]. These diverse enzymatic activities underpin isoform-specific functions in mitochondrial homeostasis, chromatin organization, immune regulation and cellular stress responses [14–17]. These findings have reframed sirtuins as versatile metabolic regulators not merely chromatin-associated deacetylases.

A defining feature of mammalian sirtuins is their distinct subcellular localization, which provides a structural basis for functional specialization. Despite sharing an NAD^+^-dependent catalytic mechanism, individual sirtuins occupy discrete compartments: SIRT1, SIRT6 and SIRT7 are primarily nuclear; SIRT3, SIRT4 and SIRT5 are localized to mitochondria; and SIRT2 is predominantly cytoplasmic [18]. This compartmentalization restricts substrate accessibility and aligns each isoform with distinct metabolic environments. Increasing evidence suggests that nuclear, cytosolic and mitochondrial NAD^+^ pools are dynamically regulated and partially insulated rather than freely interchangeable, rendering sirtuin activity highly sensitive to compartment-specific NAD^+^ availability [19]. Direct measurement of compartment-specific NAD^+^ dynamics in vivo, however, remains technically challenging. Within the nucleus, SIRT1, SIRT6 and SIRT7 integrate NAD^+^ levels with transcriptional and chromatin regulation: SIRT1 modulates stress and metabolic pathways via deacetylation of p53, FOXOs and PGC-1α; SIRT6 contributes to DNA repair, glucose metabolism and inflammatory control; and SIRT7 regulates ribosomal biogenesis and RNA polymerase I activity [20–22]. In mitochondria, SIRT3, SIRT4 and SIRT5 regulate oxidative phosphorylation, fatty acid oxidation, amino acid metabolism and the urea cycle through NAD^+^-dependent deacylation and ADP-ribosylation reactions [23–25]. SIRT2, positioned in the cytoplasm, modulates cytoskeletal organization, cell-cycle progression and stress responses through deacetylation of non-histone substrates [26]. This spatial organization establishes a functional division of labour in which NAD^+^ acts as a shared metabolic currency, while subcellular localization confers regulatory specificity.

Despite extensive investigation of individual isoforms, current understanding of sirtuin biology is fragmented by isoform-centric and disease-specific perspectives. This reductionist approach has generated seemingly contradictory findings, where the same sirtuin may exert protective or deleterious effects depending on tissue, metabolic state or timing, which points to the need for a unifying conceptual framework. This review conceptualizes the sirtuin family as a distributed metabolic sensing network that connects NAD^+^ availability with transcriptional, epigenetic and post-translational regulation across subcellular compartments [27]. This view rests on the idea that spatially segregated NAD^+^ pools and compartment-specific substrate landscapes confer functional specificity despite a shared catalytic dependency [18, 28, 29]. From this perspective, dysregulation of sirtuin-mediated metabolic sensing contributes to disease initiation and progression differently depending on tissue metabolic state, disease stage and stress duration, shaping whether cells undergo adaptive repair, maladaptive remodeling or irreversible decline [30]. This review integrates these principles to show how compartmentalized sirtuin activities collectively shape metabolic and stress responses across diseases. However, the relative contribution of each isoform to specific metabolic states is poorly quantified, and whether functional redundancy among compartmentalized sirtuins exists is largely unexplored. The available evidence does not yet permit definitive conclusions on either point, and we acknowledge this as an explicit limitation of the present synthesis. Compartmentalization itself provides a principled basis for approaching these gaps: if sirtuin function is spatially defined, then isoform-specific modulation, rather than global NAD^+^ elevation, offers a rational route to resolving relative contributions and redundancy experimentally. By framing sirtuins as context-sensitive integrators of metabolism and cell fate, this review aims to provide a coherent conceptual foundation for interpreting the expanding literature and to identify principles that may guide the development of more selective sirtuin-targeted interventions. A central thesis is that the limited and often heterogeneous clinical efficacy of global NAD^+^-boosting strategies is not incidental, but a predictable consequence of compartmentalization. Because global NAD^+^ elevation is blind to subcellular compartment, it necessarily co-activates sirtuin isoforms with opposing, compartment-specific actions. Isoform-selective, compartment-aware modulation therefore represents the more rational route to therapeutic translation.

## Biochemical activities and regulatory logic of the sirtuin family

### Catalytic repertoire and acyl-lysine specificity

As core components of a distributed NAD^+^-sensing network, individual sirtuin isoforms are distinguished first by their catalytic preferences and subcellular compartmentalisation. Although sirtuins were initially defined as NAD^+^-dependent deacetylases, it is now recognized that the mammalian sirtuin family catalyzes a broad spectrum of lysine deacylation reactions beyond canonical deacetylation [31]. Individual isoforms show clear substrate preferences, acting on a variety of acyl-lysine modifications. This catalytic diversity partitions sirtuin function within the family, helping to account for why specific isoforms preferentially regulate chromatin structure, metabolic enzymes or stress-response proteins [32–34].

However, catalytic preference alone does not fully explain sirtuin functional specificity. Nuclear sirtuins (SIRT1, SIRT6 and SIRT7) mainly regulate acetylated histones and transcriptional regulators, shaping gene expression programs, chromatin accessibility and transcriptional memory [35]. By contrast, mitochondrial sirtuins (SIRT3-5) regulate oxidative metabolism, amino acid catabolism and redox balance through deacylation of metabolic enzymes. SIRT2, which is largely cytosolic but stress-responsive, targets both metabolic enzymes and structural proteins involved in cell-cycle progression and stress adaptation [36]. Moreover, even when both catalytic preference and subcellular localization are considered, the biological output of sirtuins is not solely determined by their enzymatic properties. Because many lysine acylations arise from metabolic flux rather than dedicated enzymatic writing, sirtuin activity is highly sensitive to substrate availability [25].rather than total [37].

### Subcellular localisation and compartmental NAD^+^ pools

A defining feature of the sirtuin family is a largely conserved subcellular compartmentalisation, although this segregation is not absolute as noted above. Despite a conserved catalytic core and obligate dependence on NAD^+^, sirtuins are predominantly segregated into the nucleus (SIRT1, SIRT6 and SIRT7), mitochondria (SIRT3-5) and cytosol (SIRT2). This spatial separation constrains substrate access and couples each isoform to a distinct metabolic and redox microenvironment [38].

NAD^+^ is not uniformly distributed within the cell. Nuclear, cytosolic and mitochondrial NAD^+^ pools are dynamically regulated and only partially equilibrated [39]. Accordingly, sirtuin activity reflects local NAD^+^ availability within specific compartments not total cellular NAD^+^ abundance. Global elevations in NAD^+^ may preferentially activate specific sirtuins, depending on which compartmental pools and which isoforms are expressed or poised for activation [40].

This spatial coupling helps explain why identical mechanistic perturbations can produce divergent sirtuin-dependent responses across tissues. For example, viral infection may preferentially suppress SIRT3–5 activity by depleting mitochondrial NAD^+^, while leaving nuclear sirtuins largely intact [41]. In contrast, chromatin stress or DNA damage can selectively engage nuclear sirtuins even when mitochondrial metabolism is preserved. Thus, NAD^+^ availability is interpreted locally, allowing sirtuin activity to be tuned by compartment-specific metabolic cues instead of a global switch.

### Regulation of sirtuin activity beyond expression levels

Sirtuin activity is regulated through post-translational modifications, protein–protein interactions, subcellular relocalisation and substrate availability. Accordingly, sirtuin protein abundance correlates poorly with functional output, reflecting the importance of regulatory mechanisms that operate at the level of enzyme activation and substrate engagement [42].

Several sirtuins undergo context-dependent stabilisation or degradation through phosphorylation- or ubiquitination-dependent pathways that couple protein turnover to stress signalling. Similarly, interactions with scaffolding proteins or transcriptional complexes can restrict sirtuins to defined substrate pools, conferring selectivity that is not evident from in vitro enzymatic assays [43]; these regulatory layers are particularly prominent for sirtuins acting on non-histone substrates, where transient interactions and local concentration effects dominate.

An important methodological implication of this complexity is that genetic ablation and pharmacological inhibition of sirtuins are not mechanistically equivalent [44]. Genetic ablation abolishes both catalytic and non-catalytic functions and permits compensatory rewiring over developmental or chronic timescales, whereas small-molecule modulators typically impose acute, substrate-selective perturbations that may preferentially affect specific deacylation reactions. Failure to distinguish between these modes of perturbation has contributed to apparent inconsistencies in the literature. This distinction is not merely technical. It reflects the underlying principle that sirtuin output is shaped by regulatory context beyond catalytic activity alone. Integrating genetic and pharmacological evidence within a unified framework that accounts for substrate availability, subcellular localisation and compensatory adaptation is therefore essential for resolving contradictions and mapping the true regulatory logic of the sirtuin family. However, the relative contribution of each regulatory layer (transcriptional abundance, post-translational modification, subcellular redistribution, and local NAD^+^ availability) to net sirtuin output is poorly defined for most isoforms, and whether these layers operate hierarchically or redundantly under different physiological conditions has not been established.

### Family-level design principles and the basis of functional diversity

The biochemical and organizational features of the sirtuin family indicate that functional specialization emerges from the interplay of catalytic repertoire and subcellular localization (which jointly determine substrate access and regulatory context) rinstead of isoform identity alone. Sirtuin activity is in turn licensed by compartment-specific NAD^+^ pools and by the metabolic processes that generate acylated substrates, linking enzymatic output to local metabolic state. Superimposed on these constraints, regulatory inputs at the level of protein stability, complex formation and substrate access introduce additional layers of control that can override changes in gene expression.

These principles help explain the functional divergence observed across sirtuin biology. Conflicting outcomes attributed to the same sirtuin more often reflect differences in tissue type, metabolic state, stress duration or the subcellular compartment engaged, not genuine functional contradiction [45]. Acute versus chronic perturbations, for example, can engage distinct regulatory layers; similarly, the same sirtuin may fulfil fundamentally different roles in immune, stromal and parenchymal cells. From a translational perspective, this family-level logic has important implications. Interventions that globally elevate NAD^+^ are likely to activate multiple sirtuins in a compartment- and tissue-biased manner, whereas isoform-selective modulators must contend with substrate selectivity and cell-type- and state-dependent dual functions [46]. Accordingly, predicting therapeutic efficacy and avoiding unintended consequences requires an integrated understanding of catalytic specificity, subcellular localization and metabolic state.

These biochemical and regulatory principles frame the subsequent discussion of sirtuin function in metabolism, inflammation and disease. Instead of being considered in isolation, sirtuins can be viewed as a distributed network of NAD^+^-licensed regulators whose collective behaviour emerges from spatial organisation and metabolic signalling.

## Mechanisms of metabolic regulation by sirtuins

Building on the compartmentalized architecture outlined above, we next consider how individual sirtuin isoforms (operating within a distributed NAD^+^-sensing network) coordinate glycolysis, fatty acid oxidation, and mitochondrial quality control across subcellular compartments. Across mammalian cells, metabolic adaptation to hypoxia, inflammation and oncogenic stress is frequently initiated at the level of glycolysis. Enhanced glycolytic flux supports rapid ATP production under oxygen limitation and generates metabolic intermediates and redox signals that influence downstream biosynthetic and oxidative pathways, including lipid metabolism. As such, glycolysis is an entry point at which cellular stress responses intersect with broader programs of metabolic reprogramming. Sirtuins, as NAD^+^-dependent deacylases distributed across the nucleus, cytosol and mitochondria, are well positioned to modulate this early metabolic response. Through regulation of hypoxia-responsive transcriptional programs and post-translational control of key glycolytic enzymes, individual sirtuins influence the magnitude and routing of glycolytic throughput. These early sirtuin-dependent effects on glycolysis shape both immediate energy production and the redox state and carbon availability that condition subsequent engagement of lipid anabolic and catabolic pathways.

### HIF-1α-centered control of glycolytic activation

HIF-1α functions as a master regulator of oxygen homeostasis that couples hypoxia to metabolic reprogramming, initiating glycolytic activation while shaping downstream carbon flux and redox balance [47]. In the nucleus, SIRT1 limits acetylation-dependent stabilization of HIF-1α, such that reduced SIRT1 activity is associated with prolonged HIF-1α persistence and enhanced pro-glycolytic transcriptional programs, whereas restoration of SIRT1 restrains glycolytic flux in disease-relevant contexts [47].

SIRT6 provides a parallel chromatin-based layer of control by repressing HIF-1α target genes through promoter-associated histone deacetylation, thereby constraining the amplitude of hypoxia-induced glycolytic programs under normoxic or low-stress conditions [48–50]. Consistent with this model, loss of SIRT6 releases this transcriptional restraint under hypoxic or inflammatory pressure and is linked to exacerbated glycolytic activation and worsened inflammatory pathology in ulcerative colitis [51].

**Fig. 2:**
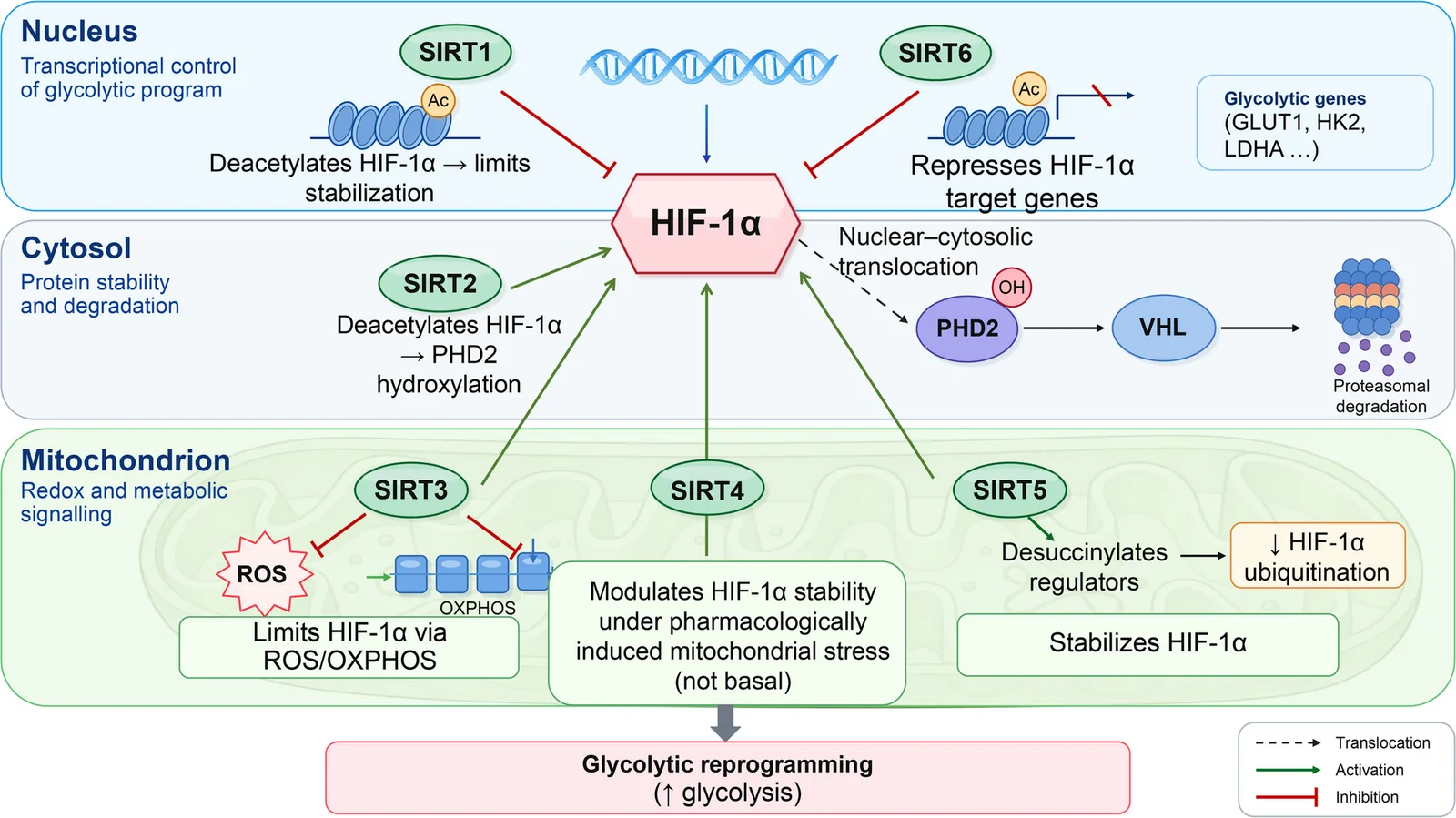
Multi-compartmental sirtuin control of HIF-1α-dependent glycolysis. Sirtuins across nuclear, cytosolic and mitochondrial compartments converge on HIF-1α to regulate glycolytic flux through coordinated transcriptional, post-translational and redox-dependent mechanisms. Distinct sirtuin isoforms modulate HIF-1α stability, activity and chromatin output via NAD^+^-dependent deacetylation, deacylation, hydroxylation and ubiquitination, collectively shaping hypoxic adaptation and metabolic homeostasis

Beyond transcriptional regulation, sirtuins also gate HIF-1α proteostasis. In the cytosol, SIRT2-mediated deacetylation of HIF-1α enhances its coupling to the oxygen-sensing machinery by promoting PHD2-dependent hydroxylation and subsequent VHL-mediated degradation, thereby limiting excessive HIF-1α accumulation in tumour contexts [52]. Mitochondrial sirtuins provide an additional layer of regulation by modulating redox and metabolic signals that influence HIF-1α stability. Through regulation of mitochondrial respiration and reactive oxygen species, SIRT3 indirectly tunes HIF-1α activity during hypoxic stress and the associated glycolytic and angiogenic responses [53]. In metabolic disease models, loss of SIRT3 has been linked to enhanced HIF-1α–LIPIN1 signalling, suppression of PPARα-dependent oxidative pathways and increased lipid uptake, collectively contributing to fatty liver progression [54]. Similarly, SIRT5 contributes to hypoxic adaptation by desuccinylation upstream regulators that reduce HIF-1α ubiquitination and degradation, thereby enhancing HIF-1α stability and promoting glycolytic rewiring in colorectal cancer [55]. These findings support a multi-compartment model in which nuclear transcriptional control, cytosolic regulation of protein stability and mitochondrial redox signalling jointly determine the magnitude and duration of HIF-1α-driven glycolysis. Recent work further indicates that SIRT4 can modulate HIF-1α stability under pharmacologically induced mitochondrial stress, but not under basal conditions [56] (Fig. 2).

### Sirtuins regulate glycolytic checkpoints and NAD^+^/redox balance

Aerobic glycolysis is increasingly understood as a process governed by discrete, rate-limiting enzymatic checkpoints, not as a uniformly regulated metabolic programme. While hypoxia-inducible factor 1α (HIF-1α)-driven transcription establishes a permissive framework for glycolytic activation, accumulating evidence indicates that the magnitude and routing of glycolytic flux are ultimately determined at key enzymatic nodes. In this checkpoint-centric model, sirtuins operate in parallel with transcriptional programs as NAD^+^-sensitive modulators of glycolytic throughput. By sensing changes in redox state and lysine acylation, sirtuins impose node-specific control over glycolytic enzymes that determine pathway commitment, redox balance and downstream metabolic signalling.

A clinically relevant illustration of this principle is provided by oxaliplatin-resistant colorectal cancer, in which PARP-driven NAD^+^ depletion suppresses SIRT1 activity and is associated with enhanced glycolytic throughput, whereas restoration of SIRT1 attenuates glycolytic output and mitigates chemoresistance [57]. This metabolic shift cannot be explained by changes in glycolytic enzyme abundance alone, but instead reflects altered NAD^+^-dependent sirtuin activity. These observations demonstrate that modulation of sirtuin activity can reprogramme glycolytic flux independently of transcriptional induction, setting the stage for node-specific control at key enzymatic checkpoints.

At the entry point of glycolysis, hexokinase 2 (HK2) functions as a key commitment checkpoint whose activity determines whether glucose is channelled into sustained glycolytic flux. Multiple sirtuins target HK2, although the direction and mechanism of regulation are highly stress-type and tissue specific. Environmental stressors provide instructive examples of this context specificity. Low-dose arsenic exposure promotes aerobic glycolysis through engagement of a SIRT1–p53–HK2 axis, in which SIRT1 suppresses p53 expression and deacetylates p53 at lysine 382, thereby relieving p53-mediated repression of HK2 and augmenting glycolytic output [58]. In contrast, chronic hydroquinone exposure is associated with the selection of a pro-glycolytic SIRT1–HK2 programme that supports malignant progression; in this context, genetic or pharmacological inhibition of SIRT1 suppresses HK2 expression, reduces glycolytic throughput and attenuates tumorigenicity [57]. These examples illustrate how SIRT1 modulates early glycolytic commitment in a stress-dependent manner, not enforcing a fixed metabolic outcome. Regulation of HK2, however, extends beyond SIRT1, indicating that multiple sirtuin pathways converge on this entry checkpoint.

SIRT6 illustrates the stress-dependent variability of sirtuin control at glycolytic entry points. While often characterised as a transcriptional brake on glycolysis, SIRT6 can, under defined therapeutic and signalling conditions, facilitate glycolytic commitment. In EGFR-mutant non–small-cell lung cancer, sustained SIRT6 activity promotes a HIF-1α–HK2 axis that maintains glycolytic flux under EGFR inhibition, thereby contributing to erlotinib resistance. Disruption of this axis suppresses glycolysis and restores drug responsiveness, which indicates that SIRT6 does not impose a fixed metabolic outcome but instead modulates glycolytic entry in a chromatin- and stress-dependent manner [59]. By contrast, in inflammatory glioblastoma contexts, SIRT6 acts as a chromatin-level constraint on glycolytic entry. Through cooperation with the transcription factor MZF1, SIRT6 reinforces nucleosomal occupancy at the HK2 promoter, thereby limiting HK2 inducibility and restraining glycolytic activation [60]. These divergent observations likely reflect the capacity of SIRT6 to integrate transcription factor context, chromatin accessibility and stress signalling to shape HK2 output, rather than enforcing a fixed regulatory direction. Accordingly, HK2 functions as a tunable commitment step that couples NAD^+^/acetylation state with tissue- and insult-specific regulatory control.

Beyond the entry checkpoint at HK2, control of glycolysis is further refined at its distal steps. Lactate dehydrogenase A (LDHA) and pyruvate kinase M2 (PKM2) act as key regulatory nodes where glycolytic flux intersects with redox balance and signalling. LDHA supports lactate production by regenerating NAD^+^ from NADH, thereby influencing the NAD^+^/NADH ratio that governs sirtuin activity. This relationship is reciprocal, as sirtuin-dependent metabolic state also feeds back to shape LDHA function. Beyond direct transcriptional control of LDHA, sirtuins also regulate LDHA-centred pathways through chromatin-mediated feedback. For example, loss of SIRT6 increases histone H3K9 acetylation at the Got1 promoter via TIP60, activating a Got1–LDHA axis that supports elevated glycolytic output in disease settings [61]. In addition to transcriptional and chromatin-based regulation, LDHA activity is also directly tuned by sirtuin-dependent post-translational modifications. In aggressive prostate cancer, SIRT5 functions as an LDHA desuccinylase, at lysine 118, linking LDHA succinylation to enzymatic activity, lactate production and metastatic potential [62]. Thus, LDHA emerges as a terminal redox node directly coupled to NAD^+^-dependent sirtuin activity.

PKM2 offers a parallel case at the distal end of glycolysis: it controls pyruvate formation and can also enter the nucleus to support pro-growth transcriptional programs. In this setting, SIRT6 is a key regulator of PKM2 at multiple levels. One well-defined route is post-transcriptional: SIRT1 and SIRT6 deacetylate the splicing factor hnRNP A1, shifting splicing from PKM2 toward PKM1 and thereby limiting the PKM2-linked signalling state [63]. Similarly, SIRT6 can directly deacetylate nuclear PKM2 at lysine 433, promoting exportin-4–dependent cytoplasmic relocation and further restricting PKM2’s transcriptional co-activator functions [50]. However, PKM2 is not an exclusive target of SIRT6. In distinct cellular and pathological contexts, other sirtuins can engage PKM2 through alternative acetylation sites to achieve different metabolic outcomes. For example, in models of Parkinson’s disease, SIRT1 directly deacetylates PKM2 at lysines 135 and 206, reducing PKM2 enzymatic activity and lactate production, thereby constraining glial activation and neuroinflammatory pathology [64]. Beyond SIRT6 and SIRT1, SIRT2 further extends PKM2 regulation in a tissue- and stress-dependent manner. Under hepatic metabolic stress, SIRT2 deacetylation couples PKM2 acetylation status to ubiquitination and protein stability, thereby shaping glycolytic flux and energy balance [65]. In inflammatory immunity, the same modification restrains PKM2’s non-canonical nuclear kinase activity linked to STAT3 signalling and Th17-driven pathology [66]. These findings identify PKM2 as a recurrent sirtuin-sensitive node where metabolic flux control and signal transduction converge.

**Fig. 3:**
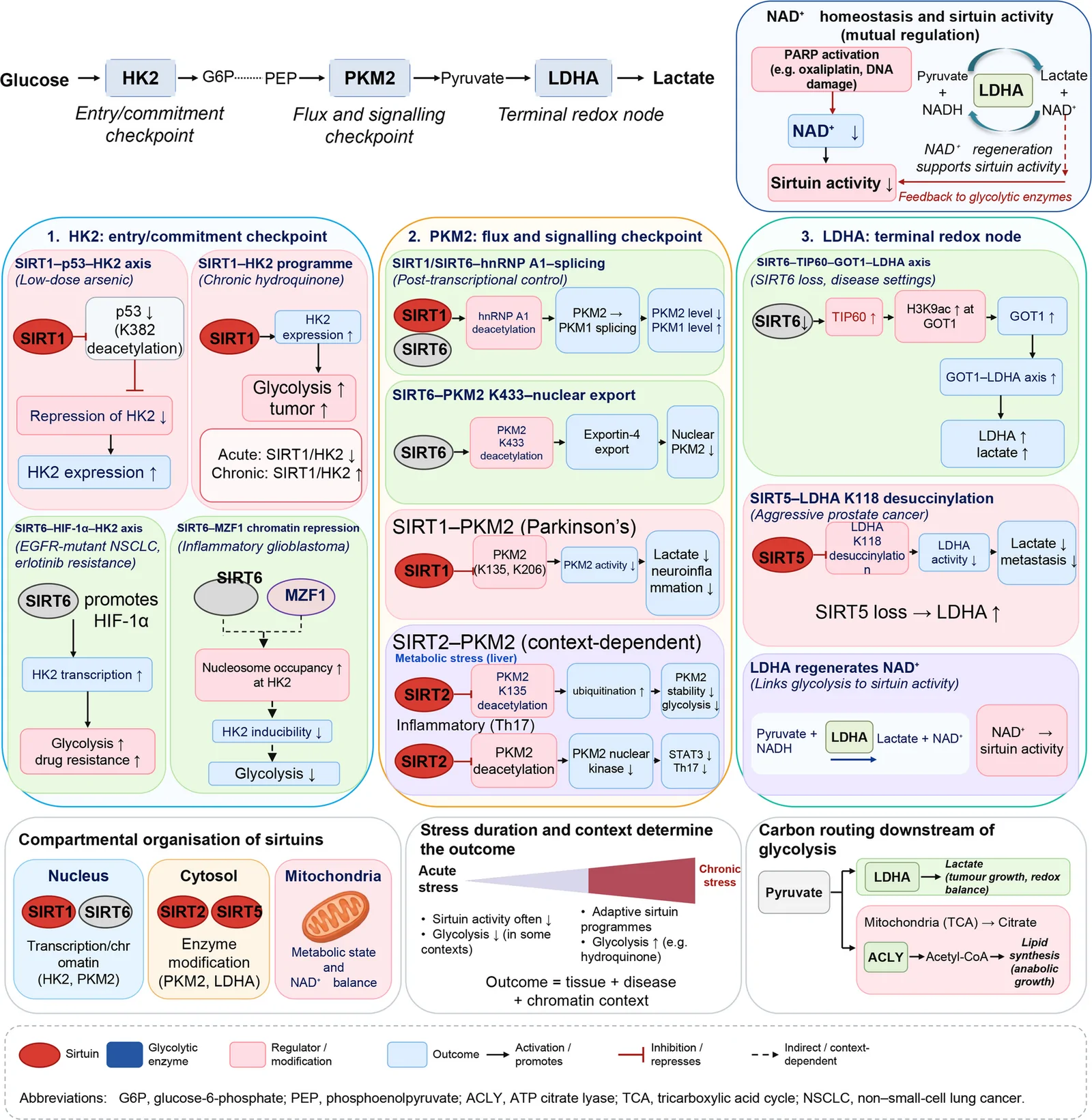
Sirtuin-mediated control of glycolytic flux at rate-limiting enzymatic checkpoints. Sirtuins regulate glycolytic output by imposing node-specific control over key rate-limiting enzymes, including HK2 at pathway entry, PKM2 at the flux and signalling interface, and LDHA at the terminal redox step. Through NAD^+^-dependent deacetylation and deacylation, distinct sirtuin isoforms tune enzyme activity, stability, localization and transcriptional coupling, enabling stress-responsive rewiring of glycolysis in response to metabolic stress and disease states

These studies support a checkpoint-centric view in which sirtuins modulate glycolytic flux through discrete, high-control nodes rather than acting as uniform activators or repressors of glycolysis. The metabolic outcome of sirtuin action is therefore jointly determined by tissue identity, chromatin state and stress signalling. This framework provides a coherent basis for understanding the opposing roles of individual sirtuins across disease settings (Fig. 3).

Instead of acting as redundant regulators, sirtuins form a compartment-stratified network whose influence shifts between mitochondrial redox control and nuclear transcriptional constraint depending on stress duration. This dynamic organisation helps explain the stress-duration-dependent effects of sirtuin isoforms on glycolytic flux. Such flux tuning also determines whether glycolytic carbon is retained for lactate production or diverted toward anabolic pathways, most notably lipid synthesis via the citrate–ACLY–acetyl-CoA axis.

### The glycolysis–lipid interface: citrate/ACLY, nuclear acetyl-CoA and epigenetic control of lipogenesis

Beyond shaping glycolytic throughput, checkpoint control determines how carbon flux is redistributed toward biosynthetic programs. One major route for such redistribution is mitochondrial citrate export and its conversion to nuclear acetyl-CoA by ATP-citrate lyase (ACLY), thereby linking glucose-derived carbon to chromatin acetylation and transcriptional control of lipogenic capacity. Within this interface, nuclear sirtuins translate acetyl-CoA availability into transcriptional control of lipid synthesis. SIRT6 constrains de novo lipogenesis by repressing SREBP1/2-driven lipogenic gene programs through promoter-associated histone deacetylation, thereby coupling glycolytic carbon routing to chromatin-level restraint of lipid anabolic output [67, 68].

While nuclear sirtuins primarily constrain lipogenic transcription at the chromatin level, mitochondrial sirtuins can influence lipid-related cell fate decisions through metabolic priming. Among them, SIRT4 has been implicated in early adipogenic commitment. In experimental models, SIRT4 promotes adipocyte differentiation by enhancing branched-chain amino acid (BCAA) catabolism, thereby increasing leucine oxidation and facilitating PPARγ-driven transcriptional programs [69]. Reduced SIRT4 expression in multiple diabetic mouse models further suggests a potential link between SIRT4-mediated BCAA metabolism and adipose tissue dysfunction, although causal relationships remain to be fully elucidated [69]. This pro-adipogenic role of SIRT4 contrasts with the predominantly antilipogenic and chromatin-restrictive actions reported for nuclear sirtuins such as SIRT1 and SIRT6, which reflects an isoform-specific division of labour in control of adipogenesis within the sirtuin family.

Extending this chromatin-based framework, SIRT6 further integrates circadian and nutrient signals to fine-tune SREBP-dependent lipogenesis. SREBP1 orchestrates transcription of FASN, ACC1, SCD1 and other lipogenic genes, and its rhythmic recruitment to chromatin is coordinated by SIRT6 together with the core clock components CLOCK and BMAL1, thereby generating daily oscillations in hepatic fatty acid and cholesterol synthesis [70]. Loss of SIRT6 disrupts these lipid rhythms and is associated with fatty acid metabolic defects, indicating that SIRT6 does not merely repress lipogenesis but helps encode circadian timing into the chromatin accessibility of SREBP programmes, thereby stabilizing lipid homeostasis across feeding-fasting cycles.

Beyond temporal control, SIRT6 reinforces lipogenic restraint through coordinated regulation of the SREBP1 signalling axis. It attenuates SREBP1 expression and processing, promotes AMPK-dependent inhibitory phosphorylation of SREBP1, and is itself repressed by intragenic miR-33b/a embedded within SREBP loci, establishing a reciprocal feedback circuit that buffers lipogenic output across nutrient states [71]. In the liver, this restraint is further implemented through direct deacetylation of key lipogenic transcriptional regulators, including SREBP1, ChREBP and LXRα, and hepatocyte-specific Sirt6 deletion markedly exacerbates diet-induced steatosis [72–74]. Consistent with a signalling-chromatin coupling mechanism, SIRT6 also activates AMPKα and inhibits mTORC1 to repress SREBP1c and improve hepatic and pancreatic coordination of glucose and lipid metabolism during high-fat feeding or starvation. Nutritional and environmental cues converge on this axis: black tea theaflavins upregulate SIRT6, enhance AMPK activity and downregulate SREBP1 and its targets to correct dyslipidaemia in obese mice [75], whereas arsenic-induced lncRNA PU.1 AS relieves EZH2-mediated repression of Sirt6, increases SIRT6 expression and limits hepatocellular triglyceride accumulation [76].

SIRT6 couples glycolytic carbon flux to chromatin-mediated lipogenesis through ATP citrate lyase (ACLY), an enzyme that links cellular metabolism to histone acetylation. ACLY generates cytosolic and nuclear acetyl-CoA from citrate, and nuclear ACLY-derived acetyl-CoA fuels histone-acetyltransferase-dependent histone acetylation at growth-promoting loci [77, 78]. Thus, citrate export from glycolysis is translated into chromatin accessibility and activation of lipogenic gene programmes. By restricting nuclear ACLY accumulation, SIRT6 is proposed to lower nuclear acetyl-CoA availability and reduce histone acetylation at pro-invasive loci, consistent with suppression of adhesion and migration programmes and reduced invasiveness. Thus, SIRT6 does not merely repress downstream enzymes, but modulates the epigenetic substrate supply that determines the transcriptional permissiveness of lipogenic pathways. In parallel with this chromatin-level regulation, SIRT6 represses FASN and other lipogenic enzymes, and SIRT6-AMPK signalling downregulates SCD1 and improves hepatic lipid handling [77–80]. These downstream effects constitute the execution layer of SIRT6-dependent lipogenic restraint. Consistent with this model, dietary polyphenols and herbal extracts such as EGCG and Rhubarb tea activate a SIRT6-AMPK-SREBP1-FASN cascade, increase hepatic SIRT6 expression and reduce triglyceride and diglyceride accumulation in models of diet-induced metabolic dysfunction [79, 80]. These studies identify SIRT6 as a chromatin-based brake on SREBP- and ACLY-driven lipogenesis, linking glycolysis-derived acetyl-CoA supply to epigenetic control of lipid metabolic output.

Beyond fatty acid synthesis, SIRT6 also extends its chromatin-based control to cholesterol biosynthesis. It is recruited by FoxO3 to the Srebp2 promoter, where it deacetylates H3K9 and H3K56, compacts chromatin and represses Srebp2 transcription [81, 82]. Consistent with this mechanism, liver-specific Sirt6 deletion elevates cholesterol levels, whereas SIRT6 overexpression corrects diet-induced and genetically driven hypercholesterolaemia, supporting a SIRT6-FoxO3-SREBP2 axis as a key checkpoint in cholesterol homeostasis. In addition, SIRT6 deacetylates and stabilises FoxO3a, enhancing its nuclear activity in pathways that coordinate lipogenesis, FAO and oxidative stress. Under exercise conditions, this axis contributes to suppression of PCSK9 through epoxyeicosatrienoic acid (EET) signalling [83]. Through these FoxO-dependent circuits, SIRT6 integrates fatty acid and cholesterol regulation within a unified chromatin-based framework of lipid metabolic control.

While mitochondrial sirtuins such as SIRT4 influence adipogenic commitment, nuclear sirtuins, particularly SIRT6, impose epigenetic restraint on both fatty acid and cholesterol biosynthesis through coordinated regulation of ACLY, SREBP1/2 and FoxO-dependent circuits.

By limiting de novo lipogenesis and buffering transcriptional responses to nutrient excess, this framework shifts cellular metabolism toward balanced lipid utilisation, coordinating lipogenic suppression with fatty acid oxidation (FAO) and mitochondrial energy metabolism.

### Sirtuin control of fatty acid β-oxidation and oxidative metabolism

In addition to lipogenic control, metabolic homeostasis requires efficient fatty acid β-oxidation (FAO). FAO provides sustained ATP production during fasting, hypoxia, inflammatory stress, and its activation represents a coordinated, metabolic shift not a simple change in fuel preference. In this process, the sirtuin family acts as a compartmentalised NAD^+^-sensitive regulatory system. Nuclear sirtuins promote transcription of FAO-related genes, cytosolic sirtuins modulate lipid utilization, and mitochondrial sirtuins directly regulate β-oxidation enzymes and redox balance. Through these coordinated actions, sirtuins adjust FAO flux to match cellular energy demand and environmental stress.

At the nuclear level, SIRT1 acts as an NAD^+^-sensitive transcriptional regulator that promotes metabolic flexibility by shifting energy production away from glycolysis and toward fatty acid β-oxidation under stress. In colorectal carcinoma, nutrient deprivation and elevated ROS induce SIRT1 upregulation, triggering deacetylation and nuclear export of β-catenin, suppression of glycolytic throughput and a compensatory increase in FAO-dependent mitochondrial ATP production [84]. Conversely, smoking-associated metabolic stress reduces SIRT1 expression, impairs PPARα-CPT1a-driven FAO and leads to pathological lipid accumulation [85]. Beyond these stress-induced changes in expression, SIRT1 activity can be further amplified through NSD2-mediated methylation of the SIRT1 activator AROS, enhancing FAO capacity and contributing to tumour metabolic reprogramming [86]. These observations support SIRT1 as a nuclear switch that links environmental stress cues to transcriptional programmes sustaining FAO competence.

SIRT6 complements SIRT1 by establishing the chromatin environment that supports FAO activation. Through direct association with PPARα and its target promoters, SIRT6 deacetylates the co-activator NCOA2 at Lys780, thereby enhancing PPARα-driven transcription of FAO genes while simultaneously repressing SREBP-dependent lipogenesis [87]. This SIRT6-PPARα axis is supported by multiple in vivo models in which pharmacological or natural compounds increase SIRT6 expression, enhance hepatic FAO and improve metabolic homeostasis in a SIRT6-dependent manner [70, 88–91]. However, small-molecule SIRT6 activators such as MDL-800 paradoxically suppress PPARα and FAO through oxidative stress-driven JNK activation, independent of SIRT6 deacetylase activity, which reflects the paradoxical and pathway-specific complexity of targeting this pathway [92]. Together, SIRT1 and SIRT6 form a nuclear regulatory layer that shifts cellular metabolism away from glycolytic and lipogenic programmes toward sustained fatty acid oxidation.

Beyond its nuclear transcriptional roles, SIRT2 also regulates FAO in the cytosol through reversible deacetylation of key FAO enzymes, thereby expanding the functional scope of the sirtuin family. In diabetic cardiomyopathy, a stress-induced mitochondrial pool of SIRT2 emerges. In these settings, SIRT2 directly deacetylates CPT2, a key enzyme, and modulates β-oxidation capacity in a tissue- and stress-specific manner [93]. This mitochondrial relocalization represents a non-canonical regulatory mechanism in which SIRT2 extends beyond its classical cytosolic functions to directly influence mitochondrial fatty acid metabolism.

At the mitochondrial level, SIRT3 acts as a central regulator that coordinates β-oxidation capacity with structural and redox adaptations necessary for sustained oxidative metabolism. In human induced pluripotent stem cell-derived cardiomyocytes, SIRT3 preserves an adult-like oxidative phenotype by deacetylating the mitochondrial fusion OPA1. This action maintains mitochondrial network integrity, enhances respiratory capacity and promotes a metabolic shift toward fatty acid oxidation while limiting glycolytic dependence [94]. Beyond cardiomyocytes, adipocyte-specific overexpression of SIRT3 increases L-carnitine levels and reduces long-chain acylcarnitine accumulation. These changes facilitate efficient long-chain FAO and improve lipid metabolism, thereby enhancing insulin sensitivity [95].

SIRT5 adds an additional enzymatic layer to mitochondrial FAO regulation through its desuccinylase activity. In diabetic cardiomyopathy, SIRT5 deficiency results in hyper-succinylation of CPT2 at Lys424, impairing FAO efficiency and leading to fatty acylcarnitine accumulation and cardiac lipotoxicity [96]. In steroid-insensitive asthma, SIRT5 upregulation enhances FAO but aggravates mitochondrial oxidative stress. Conversely, pharmacological inhibition with the selective SIRT5 inhibitor MC3482 increases succinylation of FAO enzymes, reduces FAO flux and alleviates mitochondrial dysfunction and airway inflammation [97]. These findings indicate that SIRT5 fine-tunes FAO activity through reversible lysine succinylation, but its metabolic impact is strongly shaped by tissue type and substrate availability.

Beyond its transcriptional cooperation with PPARα, SIRT6 acts as a nuclear integrator linking FAO to cellular stress adaptation across metabolic tissues. In the liver, SIRT6 preserves mitochondrial integrity and limits lipotoxic injury, thereby restraining progression from steatosis to hepatocellular carcinoma [98–100]. Mechanistically, SIRT6 activation enhances hepatic FAO capacity and stabilises NRF2-dependent antioxidant responses, improving lipid homeostasis in fatty liver models [101–103]. In addition, SIRT6 modulates endoplasmic reticulum stress through regulation of XBP1s, further coordinating metabolic and stress signaling pathways [104, 105].

The protective metabolic functions of SIRT6 extend beyond the liver to the kidney and vasculature. In diabetic nephropathy and acute kidney injury, SIRT6 preserves mitochondrial integrity and limits lipid peroxidation, thereby restraining ferroptotic injury via modulation of Nrf2/GPX4 signalling and ACMSD suppression [106, 107]. SIRT6 also maintains podocyte and endothelial function, mitigating vascular injury [108, 109]. In macrophages, SIRT6 promotes lipophagy and cholesterol efflux, reducing foam cell formation [107, 110]. Moreover, myeloid-specific Sirt6 deletion implicates SIRT6 in systemic energy expenditure and adipose–immune crosstalk [111].

**Fig. 4:**
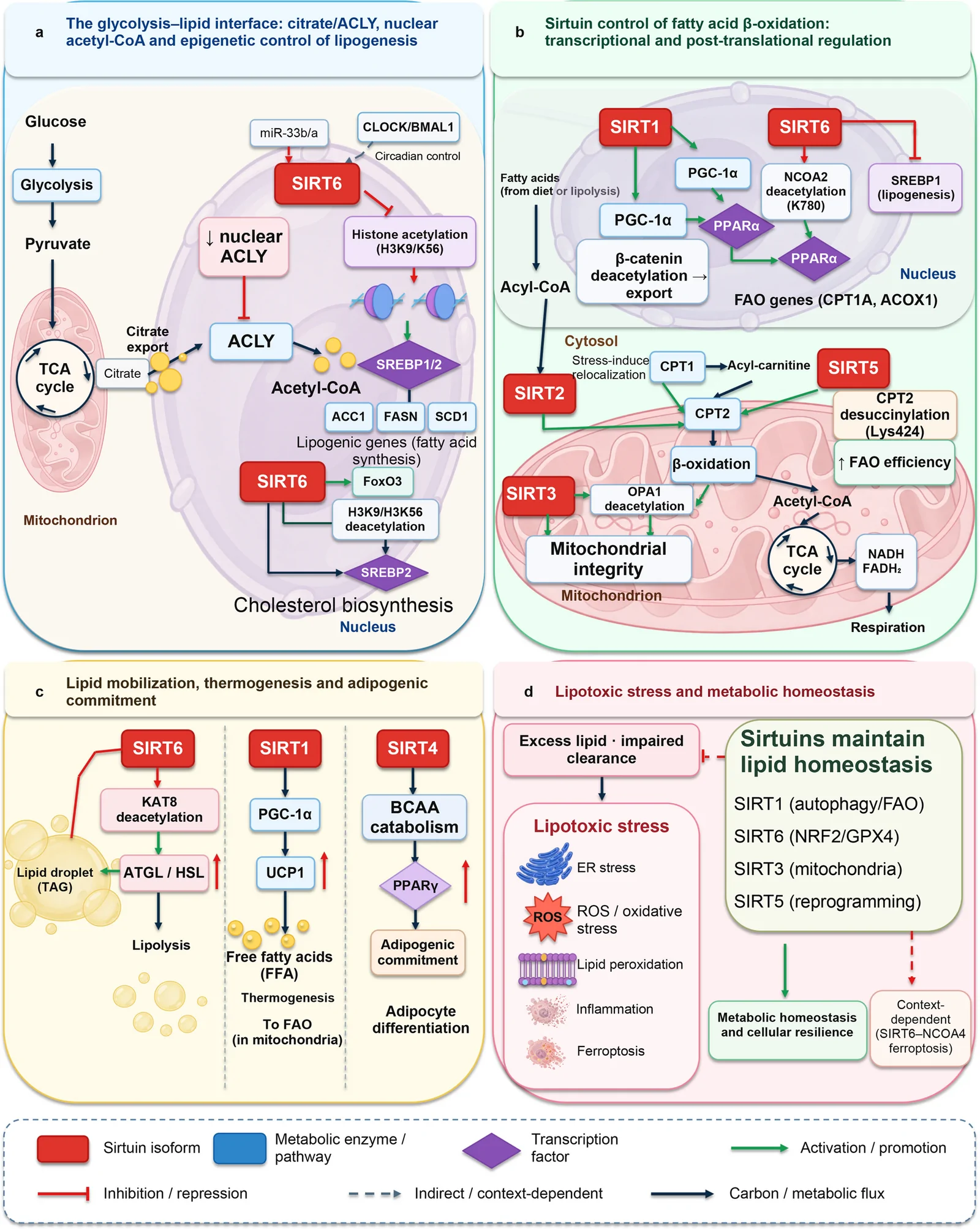
Compartmentalized sirtuin regulation of lipid metabolic homeostasis. **a** Glycolysis–lipid interface: SIRT6 couples glycolytic flux to lipogenesis by controlling nuclear acetyl-CoA availability and deacetylating histones and SREBP1/2 to restrain lipogenic gene expression. **b** Fatty acid β-oxidation: Nuclear SIRT1 and SIRT6 promote FAO gene transcription through PGC-1α/PPARα and NCOA2 deacetylation, whereas mitochondrial SIRT3, SIRT5 and SIRT2 regulate β-oxidation capacity via OPA1 deacetylation, CPT2 desuccinylation and stress-induced mitochondrial relocalization. **c** Lipid mobilization, thermogenesis and adipogenic commitment: SIRT6, SIRT1 and SIRT4 modulate lipolysis (ATGL/HSL), thermogenesis (PGC-1α–UCP1) and adipocyte differentiation. **d** Lipotoxic stress and metabolic homeostasis: Sirtuins restrain ROS, ER stress and ferroptosis to protect against lipotoxic stress, with context-dependent outcomes

FAO is thus governed by a spatially organized regulatory hierarchy spanning three subcellular compartments. In the nucleus, SIRT1 and SIRT6 promote the metabolic shift toward FAO by repressing glycolytic and lipogenic programmes while activating PPAR-dependent oxidative transcription. SIRT2 introduces a stress-responsive layer, extending sirtuin control to mitochondrial FAO enzymes through stress-induced cytoplasmic-to-mitochondrial relocalization. Within mitochondria, SIRT3 and SIRT5 fine-tune β-oxidation capacity by regulating mitochondrial structure, acylation status and redox balance. This compartmentalized sirtuin network positions FAO under integrated transcriptional and post-translational control, linking thermogenesis and energetic demands (Fig. 4).

### Mechanisms of sirtuin-mediated metabolic switching from lipogenesis to fatty acid oxidation

Activation of fatty acid oxidation requires prior mobilization of stored lipids. Lipolysis and lipid-droplet remodelling release fatty acids that fuel mitochondrial β-oxidation and thermogenic programmes in brown and beige adipocytes. These processes form a coordinated axis linking lipid mobilization, oxidative metabolism and heat production.

Within this axis, sirtuins function as NAD^+^-dependent regulators that integrate nutritional and hormonal cues to adjust lipid utilization and thermogenic capacity. SIRT1 promotes lipolysis and beige adipocyte formation through activation of PGC-1α-dependent transcriptional programmes [112]. Mitochondrial sirtuins such as SIRT3 may reinforce this response by sustaining oxidative metabolism and limiting reactive oxygen species. Although other isoforms are also implicated in adipose lipid handling, their contributions appear to depend on tissue and metabolic context.

Among nuclear isoforms, SIRT6 provides a direct link between chromatin regulation and lipid mobilization. SIRT6 enhances lipolysis partly through deacetylation of KAT8, which restores transcription of ATGL and HSL [113]. Similarly, SIRT6 promotes thermogenic gene expression by deacetylating FoxO1 and facilitating Ucp1 transcription via a proposed mTORC2–SIRT6–FoxO1 pathway [114]. Consistent with these findings, reduced SIRT6 and ATGL expression in human adipose tissue supports the translational relevance of this regulatory axis [115]. During intermittent fasting, SIRT6 is required for optimal white-to-brown adipose conversion, as Sirt6 deficiency suppresses p38 MAPK–ATF2 signalling and UCP1 expression [116].

These data identify SIRT6 as a nuclear coordinator of lipolysis and thermogenic reprogramming. In the broader family context, SIRT1 and mitochondrial sirtuins complement this function by maintaining PGC-1α-driven oxidative adaptation and mitochondrial integrity, illustrating a division of labour across subcellular compartments.

### Compartment-specific control of fatty acid β-oxidation and lipotoxic stress by sirtuins

Sustained fatty acid oxidation and lipid clearance are essential to prevent lipotoxic injury during chronic lipid excess. Cholesterol overload and accumulation of toxic lipid disrupt mitochondrial and endoplasmic reticulum function, promoting oxidative stress and driving inflammatory responses that contribute to the progression from steatosis to steatohepatitis.

Under these conditions, sirtuins regulate lipid turnover and mitochondrial oxidation to preserve tissue integrity. Terf2ip loss reduces Sirt1 transcription and suppresses AMPK signalling, impairing lipophagy and mitochondrial β-oxidation. Restoration of Terf2ip expression or pharmacological activation of SIRT1 reactivates lipid-clearing pathways and attenuates hepatic lipotoxic inflammation [117].

**Fig. 5:**
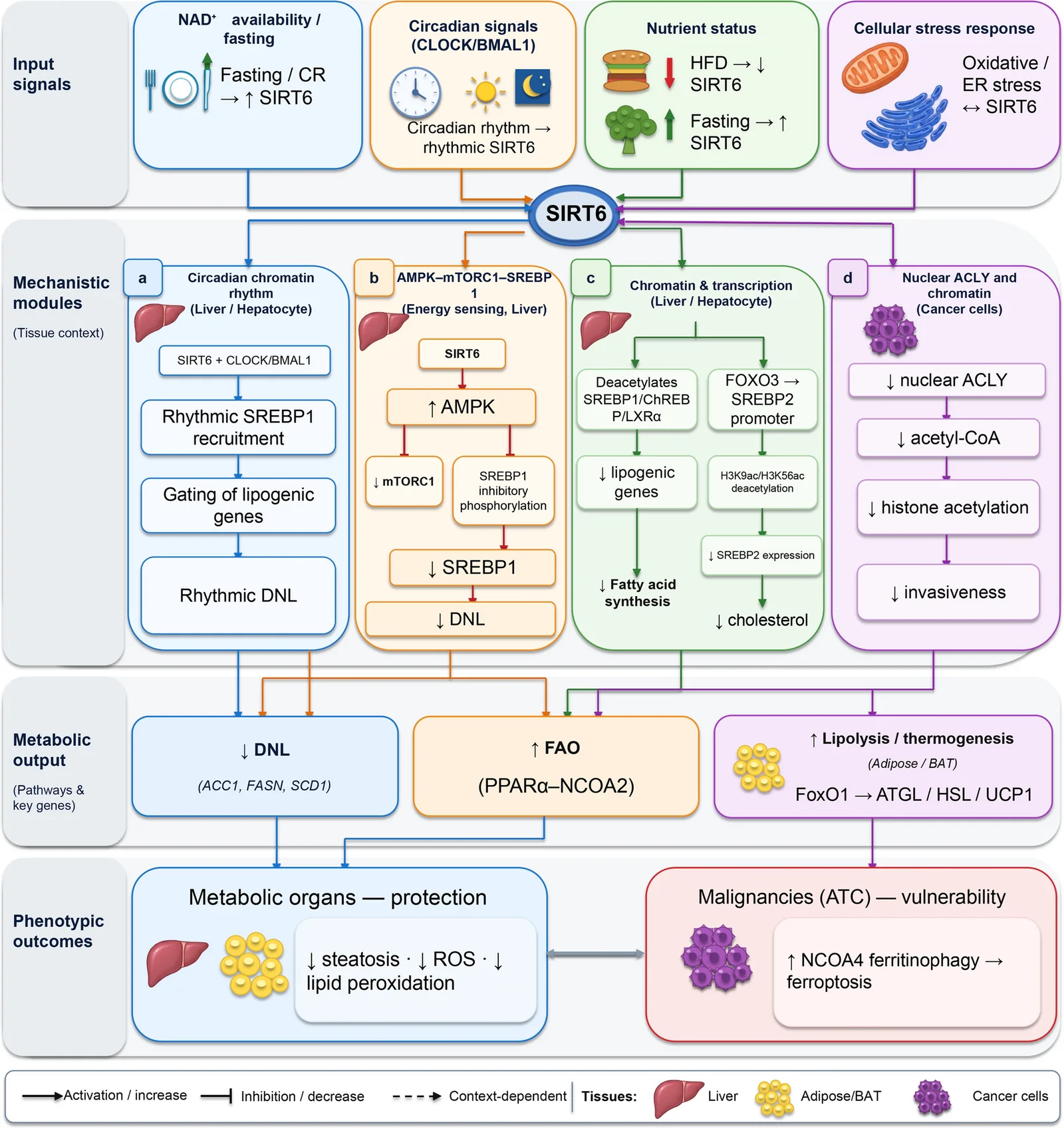
SIRT6 as an input-integrating epigenetic brake on lipogenesis and lipid-stress outcomes. Schematic overview of how SIRT6 integrates NAD^+^ availability, circadian CLOCK–BMAL1 cues, nutrient stress and oxidative/ER stress to regulate lipid metabolism across multiple tissues. Through four interconnected modules (circadian gating of SREBP1 chromatin recruitment, AMPK-mTORC1-mediated energy gating of lipogenesis, direct deacetylation of lipogenic transcription factors and chromatin marks, and regulation of nuclear acetyl-CoA supply via ACLY), SIRT6 restrains de novo lipogenesis while coordinating fatty acid oxidation, lipolysis and thermogenesis. These integrated actions promote metabolic protection in lipid-stressed tissues by limiting steatosis, oxidative injury and ferroptotic susceptibility, but can confer metabolic vulnerability in select malignancies, which highlights the context-specific outputs of SIRT6-dependent lipid regulation

SIRT6 also participates in limiting lipid-driven oxidative stress in metabolic organs. In high-fat diet-induced cardiac hypertrophy, Sirt6 deficiency increases myocardial lipid accumulation and reactive oxygen species production. Conversely, SIRT6 is associated with elevated ENDOG and SOD2 expression and reduced mitochondrial oxidative damage [118]. In the liver, SIRT6 engages a PGC-1α–ENDOG pathway linked to suppression of oxidative stress during high-fat diet-induced injury, and metformin may exert part of its hepatoprotective effect through this mechanism [118]. However, SIRT6-mediated oxidative regulation is tissue- and tumour-stage dependent. In certain tumour settings, SIRT6-driven iron mobilization and lipid-peroxidation pathways can enhance susceptibility to oxidative lipid damage. In anaplastic thyroid carcinoma, SIRT6 promotes ferroptotic vulnerability by inducing NCOA4-dependent ferritinophagy and increasing lipid peroxidation [119]. These findings reveal the disease- and tissue-specific nature of SIRT6-mediated oxidative lipid stress [118–120] (Fig. 5).

## Mechanisms of immune regulation by sirtuins

### Conceptual framework: immunometabolic mechanisms

Immune activation is inseparable from metabolic reprogramming: nutrient utilization, mitochondrial function and redox balance actively instruct effector differentiation, tolerance and tissue repair, not serving as passive by-products of inflammation. Metabolism and immunity thus operate as an integrated regulatory system, not parallel processes.

The sirtuin network extends its NAD^+^-sensing logic from metabolism into immunity: the same compartmentalised isoforms that govern glycolytic flux and mitochondrial quality control also shape inflammatory signalling, cytokine production, and immune cell fate decisions. Within this framework, sirtuins (SIRT1–SIRT7) function as NAD^+^-dependent sensors that translate cellular energetic status into immune outcomes. Because their enzymatic activity depends on NAD^+^ availability, they are uniquely positioned to couple metabolic flux to transcriptional control, stress adaptation and inflammatory signaling across stromal, innate and adaptive compartments.

Their actions are compartmentalized. Nuclear isoforms such as SIRT1 and SIRT6 remodel chromatin landscapes and shape cytokine programs and tolerance pathways. In contrast, cytosolic and mitochondrial members (SIRT2–SIRT5) recalibrate glycolysis, fatty acid oxidation, and organelle stress responses, thereby influencing inflammatory amplification and cell-death susceptibility. SIRT7 further links nucleolar stress to lymphocyte differentiation.

However, key questions remain unresolved: to what extent immunometabolic sirtuin effects are cell-type-specific versus conserved across immune lineages, and whether the seven sirtuins operate as independent checkpoints or as a coordinated network in which the composite NAD^+^/NADH ratio simultaneously tunes multiple isoforms to produce a unified immune output. The sirtuin family constitutes a mechanistic bridge between metabolic cues and inflammatory regulation. This conceptual integration provides the foundation in the sections that follow.

### Mechanisms in stromal and barrier compartments

Sirtuins regulate epithelial-stromal communication and barrier stability through at least two functionally distinct mechanisms: maintaining epithelial barrier integrity and restraining stromal fibroblast activation. In endometriosis, SIRT1 is proposed to promote epithelial plasticity by enabling endometrial epithelial cells to evade damage- or oncogene-induced senescence, thereby facilitating EMT-associated transcriptional programmes and invasive behaviour. Clinical lesions show increased stromal fibroblasts with reduced epithelial components, suggesting a remodelled niche that may further reinforce SIRT1-driven EMT through microenvironmental feedback. Nevertheless, this remains a correlative observation; whether the stromal expansion is a cause or consequence of epithelial SIRT1 activity has not been established [121]. Maintenance of barrier integrity involves both SIRT1-dependent epithelial plasticity and SIRT2-regulated junctional stability. In the gut, pharmacological inhibition of SIRT2 has been reported to alleviate colitis by stabilizing surface E-cadherin and strengthening adherens junctions. Genetic deletion of SIRT2 does not recapitulate this effect, and a PROTAC degrader targeting SIRT2 likewise fails to protect. The original study attributed this discrepancy to the fact that SIRT2 inhibitors suppress only some but not all SIRT2 activities, whereas complete protein ablation removes all catalytic and non-catalytic functions, emphasizing the therapeutic value of substrate-dependent inhibitors over complete SIRT2 elimination [122]. This divergence indicates that enzymatic inhibition and genetic deletion are not mechanistically equivalent, and reveals context- and substrate-dependent functions of sirtuins in barrier tissues.

Within stromal compartments, SIRT6 functions as a chromatin-based repressor of fibroblast activation. By limiting senescence-associated secretory phenotypes (SASP) and fibrogenic programs, SIRT6 helps maintain stromal homeostasis in multiple tissues [123–127]. In breast cancer, a subset of cancer-associated fibroblasts (CAFs) exhibits a senescent, metabolically reprogrammed myofibroblastic phenotype characterized by high TSPAN8 expression. In these cells, SIRT6 levels are markedly reduced, and its stability is controlled by a TSPAN8-MAPK11-RBBP6 phosphorylation pathway [123, 124]. Decreased SIRT6 correlates with enhanced protumour activity and increased secretion of SASP cytokines such as IL-6 and IL-8. Combined SIRT6 activation (MDL-800) and TSPAN8 inhibition reverses CAF-induced stemness and chemoresistance [123], providing functional rescue evidence supporting a causal role for SIRT6 loss in stromal tumour promotion.

Beyond tumour stroma, SIRT6 exerts antifibrotic and anti-inflammatory effects in multiple fibroblast-like populations. In rheumatoid arthritis, SIRT6 is downregulated in fibroblast-like synoviocytes. Restoring its expression suppresses MyD88–ERK signalling and reduces IL-6 and TNF production, thereby limiting synovial inflammation and tissue injury [125]. A similar pattern is observed in keloid fibroblasts and photoaged dermal fibroblasts. Reduced SIRT6 promotes fibroblast proliferation, collagen deposition and inflammatory cytokine release, whereas SIRT6 restoration attenuates these changes by restraining MAPK–ERK and NRF2–HO-1–NF-κB pathways [126, 127]. In hepatic stellate cells, SIRT6 directly deacetylates Smad3 to repress fibrogenic gene transcription, mitigating inflammation and fibrosis in non-alcoholic steatohepatitis [128]. These findings are consistent with a shared mechanism in which SIRT6 limits fibroblast activation and inflammatory signalling across tissues, thereby restraining pathological fibrosis. However, it remains unclear whether SIRT6 activity in distinct fibroblast populations is governed by shared upstream signals or tissue-specific regulatory inputs, and whether these mechanisms are causally linked across acute and chronic fibrotic settings.

Sirtuins operate at both sides of the stromal–epithelial interface: SIRT1 and SIRT2 modulate epithelial plasticity and barrier integrity, whereas SIRT6 functions as a broadly acting repressor of fibroblast and stellate cell activation. However, the functional crosstalk between these two compartments is largely unexplored; for instance, whether SIRT1-driven stromal remodelling in endometriosis feeds back to alter SIRT6-dependent fibroblast quiescence has not been tested. Furthermore, key mechanistic conclusions currently rely heavily on pharmacological or overexpression studies, with loss-of-function approaches sometimes yielding discordant results (as exemplified by SIRT2 in colitis). Resolving these discrepancies and achieving a genuine compartment-specific understanding will require genetic models that permit cell-type-selective deletion of individual sirtuins, combined with systematic profiling of the epithelial and stromal sirtuin interactomes across different tissues and disease states. Such efforts will be essential to transform the current descriptive knowledge into a predictive framework that distinguishes universal sirtuin functions from context-specific adaptations.

### Sirtuins as immunometabolic checkpoints in innate immunity

Sirtuins act as immunometabolic checkpoints in innate immunity by coupling NAD^+^-dependent sensing of metabolic and redox states to epigenetic and signalling outputs. In innate immune and barrier-associated cells, distinct SIRT isoforms exert cell-type- and stimulus-specific control over inflammatory activation, antiviral responses, and immune tolerance, yet the specific signals that dictate which sirtuin is engaged in a given context remain poorly defined. Broadly, nuclear sirtuins (SIRT1, SIRT6) regulate transcriptional and chromatin-based immune signalling, whereas cytosolic and mitochondrial sirtuins (SIRT2, SIRT3, SIRT5) shape metabolic checkpoints and organelle homeostasis; SIRT7 links nucleolar stress to immune differentiation, though its innate immune roles are just emerging.

SIRT1 is a prototypical nuclear regulator of antiviral innate immunity: it directly deacetylates IRF3 and IRF7, thereby restraining virus-induced type I (IFN-I) interferon signalling, and its inhibition or genetic deletion enhances IFN-I responses and antiviral defence [129]. Beyond antiviral responses, SIRT1 also contributes to antibacterial immunity. In murine pneumonia model, acute *Pseudomonas* infection induces a functional decline in SIRT1 protein, which correlates with reduced neutrophil recruitment and an immature, low-MPO phenotype, ultimately impairing bacterial clearance and worsening disease severity [130]. SIRT1 protein levels decline during infection even in mice with genetic overexpression, suggesting that therapeutic strategies may need to stabilize SIRT1 protein not merely enhancing its transcription. These findings position SIRT1 as a signalling-level regulator of both antiviral and antibacterial innate responses. However, the endogenous contribution of SIRT1 to immune homeostasis under physiological conditions, as opposed to acute infection models, is largely unexplored.

Sirtuin-mediated innate immune control extends beyond nuclear signal transduction to metabolic and organelle stress checkpoints, particularly in macrophages. This shift from nuclear to cytosolic regulation is exemplified by SIRT2.

SIRT2 directly couples glycolytic flux to phagocytic function. Under acute ethanol exposure, SIRT2 deacetylates the glycolytic enzyme PFKP at K394/395, reducing glycolytic flux and impairing Atg4B-dependent LC3 activation [131]. This suppression of LC3-associated phagocytosis compromises bacterial clearance in murine sepsis models [132]. This work illustrates how a cytosolic sirtuin can regulate innate immunity by linking glycolysis to non-canonical autophagy, bypassing classical interferon pathways. SIRT2 tunes cell-death thresholds within pathogen survival niches. During Brucella abortus infection, elevated SIRT2 in bovine placental trophoblasts promotes mitochondrial apoptosis; paradoxically, pharmacological SIRT2 inhibition further enhances apoptotic signalling while reducing intracellular Brucella survival. This paradoxical result suggests that SIRT2 restrains apoptosis at a level that favours pathogen persistence, and that selective intervention may disrupt this balance [131].

Recent evidence identifies SIRT2 as a cytosolic checkpoint on DNA sensing. It is currently the only sirtuin reported to directly suppress the cGAS–STING pathway through cytosolic condensate regulation [133]. Mechanistically, SIRT2 deacetylates G3BP1 at K257, K276, and K376, disrupting cGAS–G3BP1 condensates required for efficient DNA binding and cGAS activation. Pharmacological inhibition of SIRT2 with AGK2 enhances IFN responses and protects mice from HSV-1 infection, which reveals an isoform-specific antiviral mechanism that is distinct from the nuclear IRF regulation by SIRT1 and the mitochondrial metabolic checkpoints controlled by SIRT3 and SIRT5 [133].

SIRT3, a mitochondrial sirtuin, coordinates efferocytosis and inflammasome activation with organelle homeostasis in innate immune cells. In cardiac macrophages, SIRT3 promotes MerTK-dependent efferocytic clearance of apoptotic cardiomyocytes and resolution of inflammation; Sirt3 deficiency exacerbates angiotensin II-induced cardiac inflammation by impairing this clearance and disrupting mitochondrial iron homeostasis [134]. Mechanistically, SIRT3 deacetylates Frataxin (FXN) to limit mitochondrial iron accumulation and support phagocytic clearance. Independently, during Salmonella Typhimurium infection, SIRT3 deacetylates NLRC4, promoting NLRC4 activation, ASC oligomerization, and IL-1β production; Sirt3 deficiency impairs this inflammasome response [135]. These findings identify SIRT3 as a mitochondrial checkpoint that integrates iron metabolism, efferocytosis, and inflammasome signalling. However, whether the efferocytic and inflammasome functions are mediated by distinct SIRT3 substrates or converge on common downstream pathways remains to be determined.

SIRT5 provides an additional layer of metabolic control over innate inflammatory responses under oxidative and environmental stress. In eosinophilic asthma, SIRT5 overexpression in OVA-induced mouse models and HDM-stimulated airway epithelial cells suppresses eosinophilic inflammation by limiting ROS accumulation and activating the Nrf2–HO-1 antioxidant pathway [136]. Beyond the airway, SIRT5 also supports vascular homeostasis under systemic stress. In simulated microgravity, SIRT5 downregulation increases lysine succinylation of ERO1A at K396, promoting endothelial apoptosis and vascular dysfunction, whereas SIRT5 restoring expression reverses these effects [137]. These findings identify SIRT5 as a metabolic stress‑responsive regulator that preserves tissue integrity by constraining oxidative damage and cell death. However, current evidence relies heavily on overexpression and systemic stress models; the role of endogenous SIRT5 in basal innate immune homeostasis remains unclear.

Although much innate immunity research has centred on macrophages, SIRT6 plays a key role in preserving endothelial and epithelial barrier homeostasis under inflammatory and metabolic stress. In lung microvascular endothelial cells, SIRT6 overexpression or pharmacological activation (UBCS039) reduces LPS-induced VCAM1 expression and monocyte adhesion by inhibiting NF-κB signalling [138]. In diabetic foot ulcers, reduced SIRT6 expression correlates with impaired healing, restoring SIRT6 activates Nrf2 signalling, limits oxidative stress and promotes angiogenesis [139]. During dengue virus infection, SIRT6 suppresses excessive innate immune activation by interfering with NF-κB p65 DNA binding and attenuating RLR- and TLR3-driven cytokine production [140]. In the intestinal epithelium, SIRT6 maintains R-spondin-1 expression and protects against inflammation-induced apoptosis [141]. These findings position SIRT6 as a brake on barrier‑associated inflammatory activation and a promoter of tissue repair. However, most studies employed overexpression or pharmacological activation; the contribution of endogenous SIRT6 to barrier immune homeostasis under steady state remains to be rigorously tested.

Beyond barrier regulation, SIRT6 exerts stimulus- and disease-duration-dependent immunometabolic control in macrophage-related inflammation. In atherosclerosis, deletion of Sirt6 in bone marrow-derived cells increases MSR1 expression, enhances oxidised LDL uptake and promotes foam cell formation through derepression of c-Myc, a target normally restrained by SIRT6 [142]. During acute inflammatory stimulation with LPS, SIRT6 accumulates at the perinuclear endoplasmic reticulum and promotes TNF maturation via its demyristoylase activity, whereas pharmacological inhibition limits TNF release and alleviates sepsis and obesity-associated inflammation. In contrast, under chronic inflammatory conditions, SIRT6 supports resolution and tissue protection. It has been reported to enhance efferocytosis by facilitating miR-216/217 processing and activating the DEL-1–CD36 axis, although the underlying enzymatic activity responsible and the direct target remain to be defined [143]. In the kidney, proximal tubular Sirt6 deletion aggravates macrophage infiltration and fibrosis [144]; in adipose tissue, SIRT6 promotes IL-4-driven M2 polarisation, correlating with improved metabolic profiles in obesity [145]. SIRT6 emerges as a macrophage-centred regulator that balances lipid handling, cytokine release, and inflammatory resolution. However, it remains to be clarified whether the apparently opposing acute (pro-TNF) and chronic (pro-resolution) functions are mediated by distinct enzymatic activities, subcellular localizations, or cell-type-specific interactors.

The sirtuin family regulates innate immunity through a hierarchical architecture that mirrors its subcellular compartmentalization. SIRT1 acts at the nuclear signalling level by directly deacetylating IRF3 and IRF7 to tune antiviral and antibacterial transcriptional responses [130, 132]. SIRT2, SIRT3 and SIRT5 operate as cytosolic and mitochondrial metabolic-organelle checkpoints: SIRT2 couples glycolytic flux to phagocytic clearance and restrains cGAS–STING DNA sensing through condensate regulation [131, 134]; SIRT3 integrates iron homeostasis with efferocytosis and inflammasome activation [135, 136]; SIRT5 preserves tissue integrity by constraining ROS accumulation and aberrant succinylation under oxidative stress [137, 138]. SIRT6, uniquely, spans multiple subcellular compartments to integrate lipid metabolism, cytokine maturation [143, 144, 146], and barrier homeostasis [139, 140, 142, 145] in macrophages, endothelial cells [141, 147]. By contrast, the innate immune functions of SIRT4 and SIRT7 remain largely uncharacterized beyond emerging evidence for SIRT7 in nucleolar stress and inflammatory gene silencing. Several gaps in the current evidence limit a fully integrated view. First, for most isoforms, endogenous loss-of-function studies under steady-state conditions are lacking; the majority of conclusions derive from overexpression, pharmacological modulation, or acute challenge models, and whether basal sirtuin activity at physiological NAD^+^ levels exerts tonic immune control has not been tested. Second, it has not been established whether the seven sirtuins operate as independent checkpoints or as a coordinated NAD^+^-sensing network in which the composite NAD^+^/NADH ratio simultaneously tunes multiple isoforms to produce a unified immune output. Third, systematic substrate profiling under identical immune-stimulatory conditions (necessary to distinguish genuine isoform-specific functions from overlapping activities) has yet to be performed for the full family. A cross-cutting observation is that the same sirtuin can exert cell-type-dependent functional divergence that, in some contexts, produces opposing outcomes. SIRT6 provides a clear illustration. In adipose tissue macrophages, SIRT6 promotes IL-4-dependent M2 polarization that maintains systemic insulin sensitivity [146]; similarly, in mast cells, Sirt6 protects against fibroinflammation by suppressing galectin-3-mediated M1 macrophage polarization [146]. A more direct example of opposing outcomes emerges in the kidney, where proximal tubular Sirt6 restrains fibrogenesis through β-catenin deacetylation [145], yet in renal macrophages Sirt6-driven M1-to-M2 polarization can paradoxically exacerbate fibrosis when M2 macrophages persist during the AKI-to-CKD transition [147]. This cell-type-dependent duality is consistent with the checkpoint concept outlined above: the same sirtuin isoform gates inflammatory outcomes through distinct substrates in different cellular compartments. From a translational perspective, systemic SIRT6 activation could simultaneously produce beneficial and adverse effects in different tissues, which emphasizes the need for cell-type-restricted therapeutic strategies.

### Mechanisms of immune tolerance and adaptive programming

Sirtuins contribute to immune tolerance at several checkpoints that map onto the same compartmentalized architecture described for innate immunity. SIRT1 maintains tissue-specific immune privilege by repressing stress ligands such as MICA/B and ULBP3. Loss of SIRT1 derepresses these ligands, which in turn activate NF-κB/STAT3 signalling, induce CXCL9/10/11 production, and promote cytotoxic CD8^+^ T-cell infiltration. Restoration of SIRT1 activity suppresses these stress signals and re-establishes local immune tolerance, identifying SIRT1 as a regulator of tissue-specific immune restraint [148]. In contrast to the tolerance-supporting role of SIRT1, SIRT2 favours pathogenic Th17 polarization in systemic autoimmunity. In systemic lupus erythematosus, SIRT2 deacetylates p70S6K and c-Jun, sustaining mTORC1–HIF-1α–RORγt signalling, enhancing IL-17A production, and suppressing IL-2. Genetic or pharmacological inhibition of SIRT2 rebalances the Th17–IL-2 axis and alleviates disease manifestations in preclinical models, which highlights isoform-specific control of effector T-cell fate decisions [149].

SIRT3 links metabolic fitness to immune tolerance in both adaptive and innate compartments. In activated CD4 + T cells, SIRT3 deacetylates and stabilises PFKFB3, sustaining glycolytic ATP supply and reducing apoptosis; accordingly, SIRT3 loss worsens antigen-induced arthritis and can be functionally rescued by restoring PFKFB3 [150]. In macrophages exposed to metabolic stress, SIRT3 limits succinate dehydrogenase hyperacetylation, thereby restraining succinate accumulation and preventing succinate-driven epigenetic silencing of the tolerogenic factor KLF4 [151]. Together, these findings place SIRT3 as a metabolic stress-responsive regulator that preserves effector viability while protecting innate tolerance programs. At mucosal surfaces, SIRT5 can shift inflammation in the opposite direction. By supporting glutaminolysis-dependent M2 polarization, SIRT5 promotes type 2-skewed inflammation in eosinophilic chronic rhinosinusitis with nasal polyps; genetic deletion reduces disease severity, which shows that individual sirtuins can either reinforce or erode tolerance depending on tissue and metabolic state [152].

**Fig. 6:**
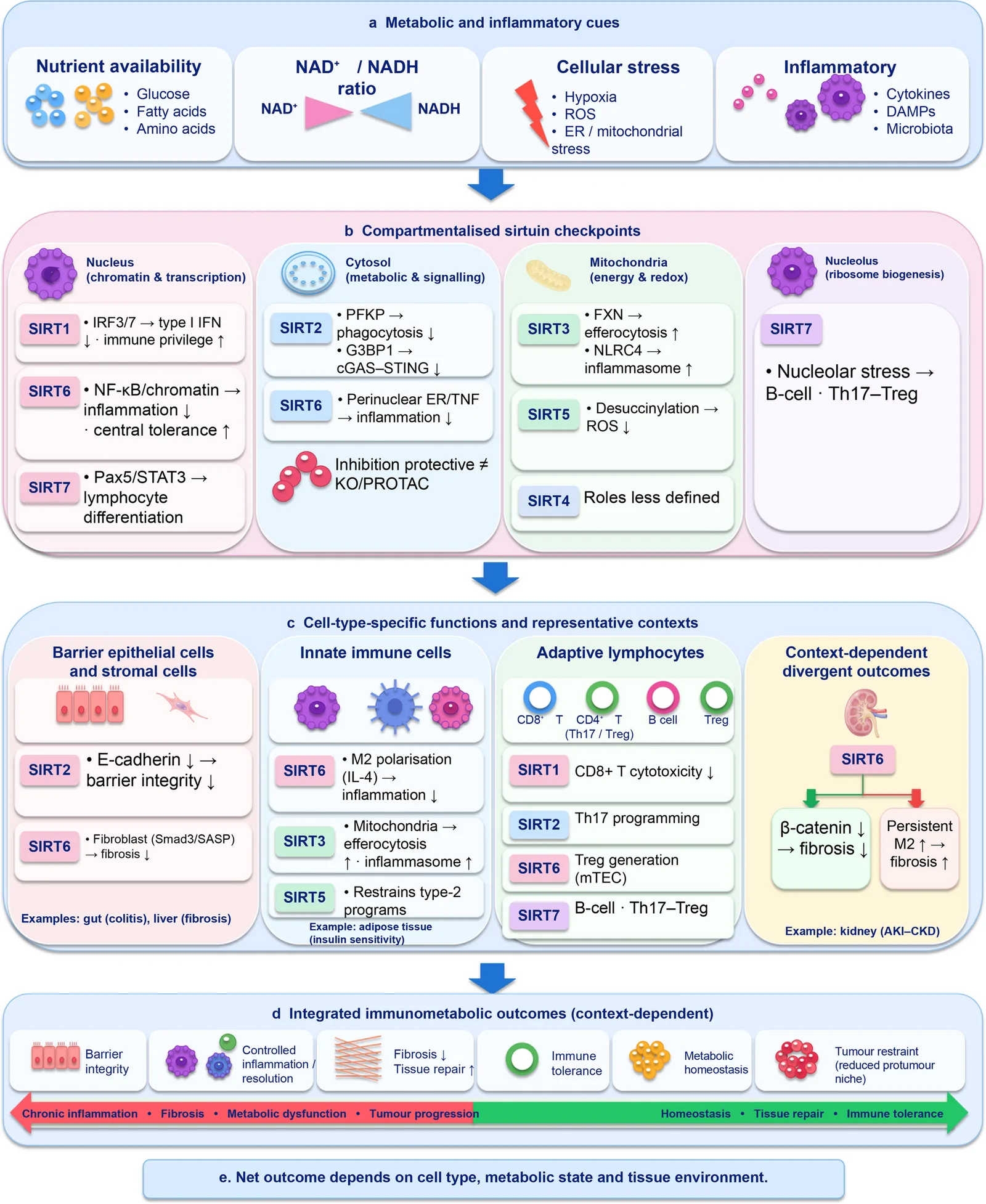
Organelle-stratified sirtuin regulation of immunometabolic disease progression. **a** Metabolic and inflammatory cues: nutrient availability, the NAD^+^/NADH ratio, and cellular and inflammatory stress provide the inputs that set sirtuin activity. **b** Compartmentalized sirtuin checkpoints: nuclear SIRT1, SIRT6 and SIRT7, cytosolic SIRT2, mitochondrial SIRT3 and SIRT5, and nucleolar SIRT7 impose compartment-specific control over immune signalling, chromatin and metabolism. **c** Cell-type-specific functions: sirtuins exert distinct roles in barrier epithelia and stroma, innate immune cells and adaptive lymphocytes, with context-dependent divergent outcomes. **d** Integrated immunometabolic outcomes: these activities converge on barrier integrity, controlled inflammation and resolution, tissue repair, immune tolerance and metabolic homeostasis. **e** Net outcome depends on cell type, metabolic state and tissue environment, uniting previously disparate autoimmune pathways and nominating sirtuin modulators as precision therapeutics for restoring tolerance without broad immunosuppression

SIRT7 contributes to immune tolerance by stabilizing lineage-defining transcription programs. In early B-cell development, SIRT7 deacetylates Pax5 at Lys198, maintaining its stability and supporting orderly B-lymphopoiesis; disruption of this axis impairs precursor differentiation and increases susceptibility to malignant transformation [153]. Beyond lineage commitment, SIRT7 also modulates peripheral T-cell balance. In immune thrombocytopenia, it desuccinylates STAT3 at Lys573, restoring the Th17–Treg equilibrium and limiting pathogenic inflammation [154]. Central tolerance is likewise shaped by sirtuin activity in non-haematopoietic compartments. In medullary thymic epithelial cells, SIRT6 supports proliferation, tissue-restricted antigen expression and regulatory T-cell generation. Conditional Sirt6 ablation disrupts these processes and results in spontaneous multi-organ autoimmunity, consistent with defective central tolerance [155]. These observations indicate that sirtuin-mediated tolerance operates at three interconnected levels: lineage stabilization (SIRT7–Pax5), peripheral effector balance (SIRT7–STAT3, SIRT2-Th17, SIRT3-KLF4) and thymic antigen-driven selection (SIRT6). This three-tier architecture is consistent with the compartmentalised NAD^+^-sensing model proposed in above: nuclear sirtuins (SIRT6, SIRT7) govern transcriptional programs of tolerance and lineage commitment, whereas mitochondrial sirtuins (SIRT3) couple metabolic fitness to tolerogenic signalling. However, several limitations temper a fully integrated picture. First, the existing data are heavily weighted toward adaptive tolerance; the role of sirtuins in peripheral tolerance mechanisms, particularly regulatory T-cell (Treg) stability and function, is largely unexplored despite the well-established dependence of Tregs on mitochondrial fatty acid oxidation, a process directly linked to SIRT3 activity. Second, for SIRT1 and SIRT7, evidence of tolerance function derives predominantly from overexpression or disease models; whether endogenous sirtuin activity at basal NAD^+^ levels contributes to tonic tolerance maintenance has not been tested. Third, a striking and as-yet-unexploited parallel with above is the bidirectional nature of sirtuin tolerance control: SIRT1, SIRT6 and SIRT7 enforce tolerance, whereas SIRT2 and, in certain contexts, SIRT5 erode it. This raises the question of whether the same NAD^+^ threshold that triggers innate inflammatory checkpoints simultaneously shifts the tolerance–immunity balance. The direction and magnitude of these effects depend on cell type, metabolic context and tissue environment (Fig. 6).

### Mechanistic regulation in the tumour immune microenvironment

The tumour immune microenvironment (TIME) is characterized by hypoxia, nutrient limitation and oxidative stress, conditions that reshape immune cell metabolism and function. Within this metabolically constrained setting, sirtuins act as NAD^+^-dependent sensors that couple energy status to chromatin regulation, signalling thresholds and cell fate decisions in tumour-infiltrating immune cells. This section examines how individual sirtuins modulate macrophage polarization, effector T-cell fitness, regulatory cell expansion and stromal–immune crosstalk in a compartment-specific manner.

SIRT1 displays compartment-specific effects in the TIME. In tumour-associated macrophages, oxidative stress disrupts extracellular SIRT1, leading to intracellular SIRT1 deficiency in M2-like TAMs. This state is associated with PD-L1 upregulation, CD8^+^ T-cell exhaustion and enhanced immune evasion [156]. In contrast, tumour-cell-intrinsic SIRT1 can reinforce immunosuppression through paracrine signalling. In colorectal cancer, SIRT1 increases CX3CL1 expression, promoting expansion of suppressive TNFRSF9^+^ Treg cells via CX3CR1–SATB1/BTG2 axis; CX3CR1 blockade enhances the efficacy of PD-1 inhibition in preclinical models [157]. Thus, these observations indicate that SIRT1 influences immune evasion through distinct mechanisms in macrophage and tumour compartments.

SIRT2 primarily regulates the metabolic fitness of cytotoxic lymphocytes in nutrient-limited tumours. In tumour-reactive CD8^+^ T cells, SIRT2 deacetylates glycolytic and mitochondrial enzymes, suppressing both glycolysis and oxidative phosphorylation. Genetic deletion or pharmacological inhibition restores proliferation and effector function, and elevated SIRT2 expression in tumour-infiltrating lymphocytes correlates with poor immunotherapy response [158]. SIRT2 also modulates tumour immune surveillance through additional mechanisms: SIRT2 inhibition induces MLH1 deficiency and enhances CD8^+^ T-cell infiltration and antitumour immunity in colorectal cancer models [159, 160], while its effects on NK cells are model-dependent, enhancing tumouricidal function in hepatocellular carcinoma but inhibiting NK-cell activity in melanoma [161, 162]. Together, these data position SIRT2 as a metabolic checkpoint that restrains adaptive and innate antitumour immunity.

Myeloid lipid metabolism is a major sirtuin-controlled route to immunosuppression in the TME. In tumour-associated macrophages, the SENP1–SIRT3 axis increases mitochondrial acetyl-CoA and supports cholesterol biosynthesis, which reinforces M2 polarization and is accompanied by reduced CD8^+^ T-cell abundance and function [163]. In bladder cancer, loss of SIRT4 is associated with impaired CD8^+^ T-cell chemotaxis and cytotoxicity together with enhanced tumour-cell proliferation, and higher SIRT4 expression correlates with a more favourable immunotherapy response [164]. In hepatocellular carcinoma, SIRT5 downregulation drives peroxisomal hypersuccinylation and excess bile acid production, promoting M2-like macrophage polarization through nuclear receptor signalling; bile acid sequestration with cholestyramine reverses this circuit and limits tumour growth in SIRT5-low models [165].

Beyond immune cell-intrinsic programmes, stromal sirtuins shape immunosuppressive niches. In breast cancer, TSPAN8-MAPK11-dependent proteasomal degradation reduces SIRT6 in a subset of myofibroblastic CAFs, activating SASP programmes with elevated IL-6 and IL-8 and increased therapy resistance; combined SIRT6 activation (MDL-800) and TSPAN8 blockade attenuates these protumour stromal features [123, 124, 166]. By contrast, in anaplastic thyroid carcinoma, tumour-cell-intrinsic SIRT6 promotes ferroptosis, which demonstrates that the impact of SIRT6 depends on the cellular compartment [119].

In summary, sirtuins shape the tumour immune microenvironment through compartment- and isoform-specific mechanisms. SIRT1 and SIRT5 influence macrophage and regulatory T-cell programmes via metabolic and extracellular signalling pathways, whereas SIRT2 and SIRT3 regulate the metabolic threshold of cytotoxic T and natural killer cells. SIRT4 links mitochondrial homeostasis to immune surveillance, and SIRT6 modulates stromal senescence and ferroptosis-associated immunogenicity. This compartment- and isoform-specific division of labour mirrors the architecture described for innate immunity and tolerance, reinforcing the concept that the sirtuin family operates as a distributed NAD^+^-sensing network whose immunological output is determined by which isoforms are engaged in which cellular compartments.

Rather than acting uniformly as pro- or anti-tumour factors, individual sirtuins adjust immune activation according to cellular context and metabolic constraints. A recurrent theme across innate immunity, tolerance and tumour immunology (as exemplified by the opposing effects of SIRT2 on NK cells in different tumour models [161, 162] and of SIRT6 in stromal versus tumour-cell compartments [120, 123]) is that the same sirtuin isoform can produce antithetical immunological outcomes depending on cell type, tissue environment and NAD^+^ availability. These mechanistic insights provide a rationale for selective therapeutic modulation, which will be discussed in the following section. The outcome of sirtuin targeting in the TIME is likely to depend on tumour type, immune composition and metabolic state, a consideration that applies equally to innate and adaptive immune contexts and underscores the need for context-guided rather than pan-sirtuin therapeutic strategies.

## Mechanistic roles in human diseases

### Metabolic and systemic diseases

When the distributed sirtuin regulatory network becomes dysregulated (whether through NAD^+^ depletion, isoform-specific loss, or aberrant acylation), its failure manifests across diverse disease states in a tissue- and context-specific manner. Metabolic syndrome and related disorders, including obesity, type 2 diabetes, metabolic dysfunction-associated steatotic liver disease and gestational diabetes, are characterized by chronic energy surplus, reduced metabolic flexibility and progressive NAD^+^ decline. These changes promote mitochondrial dysfunction, lipid accumulation and persistent low-grade inflammation, driving disease progression. As NAD^+^-dependent deacylases, sirtuins directly link cellular metabolic state to transcriptional, enzymatic and redox regulation [167, 168]. Within this context, different sirtuin isoforms contribute to metabolic control at distinct cellular levels. Nuclear SIRT1 and SIRT6 regulate gene expression programs that influence lipid and glucose metabolism; SIRT2 modulates cytosolic stress responses and metabolic enzyme activity; mitochondrial SIRT3–5 maintain oxidative metabolism and redox balance; and SIRT7 remains less well defined in systemic metabolic disease.

SIRT1 and SIRT6 play important roles in systemic metabolic protection. SIRT6 suppresses excessive glycolysis and lipid synthesis, while SIRT1 helps maintain insulin sensitivity by deacetylating key metabolic and stress-related proteins. Together, these nuclear sirtuins link reduced NAD^+^ levels to changes in gene expression and metabolic control [169, 170]. Although these findings establish SIRT1 and SIRT6 as central metabolic regulators, the direction of causality in human disease (whether sirtuin decline drives metabolic decompensation or reflects compensatory adaptation to chronic overnutrition) remains unresolved in most cases.

Mitochondrial sirtuins (SIRT3-5) provide additional metabolic support. SIRT3, the major mitochondrial deacetylase in metabolic tissues, is essential for maintaining mitochondrial function under metabolic stress. Reduced SIRT3 expression is associated with impaired oxidative metabolism and weakened antioxidant defense in several disease models, including diabetic osteoporosis [171, 172], periodontitis [173], hypoglycemia-induced astrocyte neurotoxicity [174] and renal fibrosis [171–176]. SIRT4 and SIRT5 also contribute to mitochondrial regulation, particularly in stress responses related to cell survival and metabolic stability.

SIRT2 has context-dependent and stage-dependent effects. Under hyperglycemia, it limits excessive β-cell expansion by deacetylating oxidative phosphorylation (OXPHOS) enzymes [177]. It also promotes mitochondrial biogenesis in trophoblasts through PGC-1α and suppresses TGFβ–SMAD2/3–mediated fibrosis in kidney disease. In these settings, SIRT2 appears to protect tissues by preventing excessive metabolic or stress responses.

Together, nuclear SIRT1/SIRT6 and mitochondrial SIRT3 represent key regulators of systemic metabolic stability, while SIRT2 and SIRT4/5 provide additional support in specific contexts. This helps explain why global NAD^+^ precursors such as NMN and NR show broad metabolic benefits in experimental models, whereas isoform-selective modulators may be needed for more targeted therapy.

Cardiovascular and renal diseases are closely linked to chronic metabolic stress. Long-term nutrient excess, oxidative stress and reduced NAD^+^ levels contribute to endothelial dysfunction, inflammation, cardiac remodelling and fibrosis. In these conditions, sirtuins regulate gene expression, protein modification and mitochondrial function. Nuclear SIRT1, SIRT6 and SIRT7 influence transcriptional control; cytosolic SIRT2 regulates protein acetylation; and mitochondrial SIRT3 maintains mitochondrial integrity [168].

SIRT1 and SIRT6 play important protective roles in renal and vascular disease. In diabetic nephropathy, gut-derived indole-3-propionic acid stabilizes SIRT1 by preventing phosphorylation-dependent ubiquitination; this restores PGC-1α-mediated mitochondrial biogenesis and reduces ROS-mediated glomerular injury [178]. SIRT1 also protects against toxin-induced acute kidney injury by maintaining PINK1/Parkin mitophagy [179]. In lupus-prone mice, it reduces renal inflammation by limiting NLRP3 inflammasome activation through NF-κB and ROS/TRPM2-Ca^2+^ signalling pathways [180]. SIRT6 also exerts complementary protective effects. It helps maintain mitochondrial dynamics, suppresses lipid accumulation and oxidative stress programs, and reduces vascular calcification and foam cell formation. These protective functions are often reduced in disease conditions.

SIRT2 and SIRT3 provide key post-translational and mitochondrial support. In angiotensin II-driven hypertension, SIRT2 deacetylates Septin4-K174 in podocytes, reducing PARP1/caspase-3 mediated apoptosis [181]. It also inhibits TGF-β-induced SMAD2/3 activation and limits tubular fibrosis in chronic kidney disease [182]. SIRT3 improves mitochondrial function in macrophages, decreases lactate-driven NLRP3 activation, and reduces perivascular adipose fibrosis [183]. SIRT7 protects against hyperglycemic memory in diabetic nephropathy. High glucose lowers SIRT7 expression, leading to persistent ELK1-dependent DAPK3 transcription and ongoing endothelial inflammation even after glucose returns to normal. Overexpression of SIRT7 improves renal function and reduces inflammation in preclinical models [184]. Overall, SIRT1 and SIRT6 act as main transcriptional and epigenetic regulators against cardio-renal metabolic injury. Their effects are supported by SIRT2 and SIRT3, which maintain protein stability and mitochondrial function, and by SIRT7, which helps reverse metabolic memory. This layered mechanism suggests that NAD^+^ precursors and isoform-selective modulators may help slow the progression from metabolic disorder to organ damage. The limitations of current evidence should be acknowledged: most mechanistic conclusions derive from gain-of-function or acute knockout models, and whether basal sirtuin activity at physiological NAD^+^ levels exerts tonic metabolic control in humans remains untested. Neurodegenerative disorders, in which metabolic failure and neuroinflammation converge, are discussed later [185].

SIRT1 directly regulates pancreatic β-cell function and insulin secretion. In pancreatic β cells, Sirt1 represses the mitochondrial uncoupling protein UCP2, an action that raises ATP levels and thereby couples glucose metabolism to insulin secretion; Sirt1 deficiency or inhibition impairs glucose-stimulated insulin secretion [186]. This establishes SIRT1 as a positive regulator of β-cell secretory capacity, positioning its loss as a potential contributor to β-cell dysfunction in type 2 diabetes. β-cell senescence provides a second, disease-relevant layer of sirtuin regulation. The cholesterol oxidation product 7-ketocholesterol (7-KC) accelerates pancreatic β-cell senescence by inhibiting the SIRT1/CDK4–Rb–E2F1 signalling pathway, thereby reducing insulin secretion and regenerative capacity, a mechanism linking lipid stress to β-cell aging in type 2 diabetes [187]. These findings reveal opposing inputs on the β-cell SIRT1 axis: physiological SIRT1 activity supports insulin secretion, whereas lipid-induced loss of SIRT1 signalling drives senescence, illustrating how metabolic context determines whether sirtuin activity protects or compromises β-cell function. Sirtuins are key regulators of adipose tissue remodelling in obesity, governing adipogenesis, beige adipocyte conversion and inflammation [188]. SIRT6 in particular has multiple roles in adipose tissue inflammation, where it modulates the inflammatory program of adipocytes and adipose-resident immune cells, with its loss or downregulation linked to exacerbated adipose inflammation in obesity [189]. Beyond adipocyte-intrinsic regulation, endothelial sirtuins contribute to whole-body metabolic homeostasis. Endothelial SIRT3 deficiency predisposes brown adipose tissue to whitening in diet-induced obesity: both global and brown-adipose-tissue-endothelium-specific Sirt3 knockout accelerate diet-induced weight gain, accompanied by glucose intolerance, insulin resistance and brown adipose tissue whitening [190]. This positions endothelial SIRT3 as a guardian of brown adipose tissue thermogenic capacity, linking vascular sirtuin signalling to systemic energy balance. In the liver, mitochondrial sirtuins protect against steatosis. Irisin, a skeletal-muscle-derived myokine, alleviates hepatic steatosis by activating the autophagic SIRT3 pathway, thereby improving hepatic lipid homeostasis in high-fat-diet-induced metabolic dysfunction-associated steatotic liver disease (MASLD) [191]. Together with the established roles of SIRT1/SIRT6 in hepatic lipid metabolism, this reinforces a model in which mitochondrial sirtuins (SIRT3 in particular) mediate autophagy-dependent lipid clearance that opposes steatosis progression. Across β cells, adipose tissue and liver, sirtuins exert tissue-specific and context-dependent effects: SIRT1 supports β-cell insulin secretion yet its loss also permits senescence, while SIRT3 protects both brown adipose tissue thermogenesis and hepatic lipid clearance. A notable gap is that the relative contribution of individual sirtuins to human metabolic disease (versus their well-documented roles in rodent models) is poorly quantified, and whether sirtuin-directed strategies can achieve tissue-selective metabolic benefit without systemic NAD^+^ modulation remains an open translational question.

### Cancer and therapy resistance

Cancer cells use sirtuin-dependent metabolic and epigenetic regulation to survive stress and therapy. The roles of the seven mammalian sirtuins are highly tumour-type- and treatment-dependent. SIRT6, SIRT3, and SIRT4 often act as tumour suppressors, whereas SIRT7 and frequently SIRT5 promote tumour progression. SIRT1 and SIRT2 display dual roles depending on the setting.

SIRT6 is one of the most consistent tumour-suppressive sirtuins in many cancer types. It inhibits glycolysis and acetyl-CoA-driven anabolic pathway, maintains genome stability, and limits stromal senescence. Loss of SIRT6 weakens these protective effects, while SIRT6 activators (MDL-800/811) can restore them in several models. SIRT1 shows strong tissue-type dependence. It promotes ferroptosis in glioma [192] and reduces pro-glycolytic H3K18 lactylation in gastric cancer [193], however, it can also contribute to therapy resistance, including taxol resistance through polyploid giant cancer cells [194], 5-FU resistance via p53/miR-101 [195] and escape from doxorubicin-induced senescence by restoring autophagy [196].

SIRT2 similarly displays stage- and cell-type-dependent polarity. It can be oncogenic in early neoplasia through effects on mitotic fidelity and EMT programmes (SMC1A mitotic fidelity [197]; Snail/EMT [198]), yet it can acquire tumour-suppressive roles in other settings through DNA repair or pathway decoupling (BER via OGG1 [199]; AKR1C1-STAT3 decoupling [200]). This bidirectionality can be interpreted as reprogramming of sirtuin dependencies across tumour evolution rather than as contradiction.

Mitochondrial SIRT3 and SIRT4 generally limit tumorigenesis by maintaining oxidative metabolism and imposing metabolic checkpoints. By contrast, SIRT5 often acts as an oncogenic vulnerability in solid tumours and leukaemia, where it sustains respiratory capacity and redox-supporting pathways, promotes resistance to ferroptosis, and facilitates immune evasion. SIRT7 promotes PD-L1 expression and is linked to resistance to sorafenib, gemcitabine and radiotherapy. It also accelerates PTEN degradation, supports Fascin-mediated metastasis, and dampens endoplasmic reticulum stress across multiple tumor types, including HCC, PDAC, endometrial cancer, ESCC, and melanoma [16].

The sirtuin family contributes a compartmentalized regulatory network in cancer, with distinct isoforms playing opposing roles. Nuclear SIRT6 and SIRT1 function as a metabolic-epigenetic brake, mitochondrial SIRT3 and SIRT4 act as tumour-suppressive checkpoints, SIRT5 and SIRT7 are frequently co-opted as oncogenic drivers, and SIRT2 displays bifunctional stage-dependent switching. This isoform specificity clarifies why non-selective NAD^+^ boosters yield inconsistent outcomes, whereas isoform-selective SIRT2/5/7 inhibitors, SIRT6 activators and SIRT3/4-preserving approaches are advancing as precision combination therapies. During cancer metabolic rewiring, sirtuins enforce a functional stratification: SIRT1 and SIRT6 restrain glucose-driven anabolic metabolism, SIRT3 and SIRT4 promote oxidative restraint, SIRT5 sustains respiration-linked redox networks, and SIRT2 can amplify acetyl-CoA- and proline-dependent biosynthesis. SIRT6 stands out as a consistent suppressor of tumour metabolic reprogramming, broadly curbing glycolytic and acetyl-CoA-linked anabolic drift. In contrast, SIRT2 can serve as an oncogenic accelerator in specific settings. In oesophageal squamous cell carcinoma, SIRT2 activates ATP-citrate lyase to expand cytosolic acetyl-CoA pools and promote lipogenesis; SIRT2 inhibition suppresses tumour growth [201]. In colorectal cancer, SIRT2 deacetylates IDH1 at K224 to enhance α-ketoglutarate-dependent redox homeostasis, with IDH1 hyperacetylation correlating with metastasis and poor survival [202]. In hepatocellular carcinoma, SIRT2 deacetylates mitochondrial P5CS to fuel proline-linked respiration and tumor expansion [203]. SIRT5 sustains multiple oncogenic circuits by desuccinylating and stabilising MTHFD2 in breast cancer, boosting one-carbon metabolism-derived NADPH and facilitating chemoresistance [204].

Sirtuins establish a functionally stratified network in cancer metabolism. While SIRT6, and contextually SIRT1, SIRT3, and SIRT4, typically restrain anabolic flux and enforce tumor-suppressive checkpoints, SIRT2 and SIRT5 are often co-opted to drive oncogenic pathways that enhance acetyl-CoA availability, proline metabolism, and redox support. This functional divergence clarifies the inconsistent outcomes of non-selective NAD^+^ augmentation and rationalizes the development of isoform-selective strategies, such as SIRT2/5 inhibition or SIRT6 activation, to target specific metabolic vulnerabilities. The role of any individual sirtuin is ultimately defined by tumor type, stage, and metabolic dependencies, which reflects the necessity for biomarker-guided therapeutic application. However, for most of these tumour-type-specific observations, whether sirtuin dysregulation is a driver of malignant progression or a consequence of tumour-driven metabolic reprogramming remains unresolved.

### Neurodegenerative disorders

Neurodegenerative disorders and CNS are characterized by the convergence of metabolic dysfunction, loss of protein homeostasis, and chronic inflammation, which collectively undermine neuronal and glial resilience. Within this pathological landscape, sirtuins function as NAD^+^-dependent regulators that integrate genomic, metabolic, and inflammatory responses. A compartmentalized division of labour emerges: nuclear sirtuins (SIRT1, SIRT6) govern transcriptional programs critical for long-term cell fate, while cytosolic SIRT2 and mitochondrial SIRT3 regulate axonal integrity, bioenergetics, and redox balance. Disease manifestations often reflect which cellular compartment is compromised first: nuclear instability perpetuates neuroinflammation, whereas mitochondrial failure limits the energy required for synaptic repair.

Within this framework, SIRT6 serves as a central protective factor in CNS homeostasis, with SIRT1 and SIRT3 providing contextual support via neuroimmune and mitochondrial mechanisms, respectively. For instance, in models of optic nerve injury, microglial SIRT1 deacetylates TSC2 to restrain mTORC1 activity, limiting excessive synaptic pruning and preserving retinal ganglion cell function [185]. In neuropathic pain, the ketone body β-hydroxybutyrate exerts anti-inflammatory effects by engaging SIRT3 to restore mitochondrial membrane potential and suppress microglial ROS in the spinal cord [205].

Building on this compartmentalized division of labour, the specific mechanisms through which sirtuins influence neurodegeneration can be organized along three routes: tau pathology, mitochondrial quality control and neuronal DNA repair, with NAD^+^ availability as the common thread. SIRT1 is the most consistently implicated sirtuin in tau biology, and it regulates tau through multiple distinct mechanisms. A mitophagy-based pathway links the autophagy-initiating kinase ULK1 to inhibition of pathogenic tau acetylation through the ULK1–NAD^+^/SIRT1 axis, suggesting that mitophagy restricts early tau pathology via SIRT1-dependent deacetylation [206]. SIRT1 also controls tau O-GlcNAcylation indirectly: it deacetylates CREB, inhibiting CREB phosphorylation and thereby regulating OGT-dependent tau modification, a pathway relevant to the reduced tau O-GlcNAcylation observed in Alzheimer's disease brain [207]. Through a third route, Sirt1 enhances tau exon 10 inclusion and improves spatial memory in Htau mice, linking SIRT1 activity to tau isoform balance and cognitive function [208]. In the mitochondrial compartment, SIRT3 and SIRT6 preserve neuronal bioenergetics. SIRT3-mediated deacetylation of succinate dehydrogenase subunit A (SDHA) rescues mitochondrial bioenergetics and contributes to neuroprotection in rotenone-induced Parkinson's disease models [209]. SIRT6, which has been implicated in DNA repair and has critical roles in brain development and neurodegeneration, is a key regulator of mitochondrial function in the brain, as shown by integrated transcriptomic and metabolomic analyses [210]. The NAD^+^–sirtuin axis also has direct therapeutic relevance in neurodegeneration. NAD^+^ supplementation normalizes key Alzheimer's features and DNA damage responses in an AD mouse model carrying a DNA-repair deficiency, providing in vivo evidence that restoring NAD^+^ availability counteracts neuronal DNA damage [211]. In Parkinson's disease, sirtuins are increasingly recognized as regulators of mitochondrial dysfunction, oxidative stress and α-synuclein pathology that drive dopaminergic neurodegeneration [212]. Sirtuins thus form a convergent node linking NAD^+^ metabolism, tau post-translational modification and mitochondrial quality control in neurodegeneration. The field remains limited by the dominance of mouse models over human data, and whether the protective effects observed for SIRT1/SIRT3/SIRT6 can be achieved with isoform-selective or NAD^+^-based interventions without the mixed outcomes seen with global NAD^+^ elevation remains unresolved.

### Sirtuins in cardiovascular disease

Sirtuins protect the cardiovascular system through compartment-specific actions in the endothelium, the myocardium and the cardiac mitochondrial network. In the endothelium, SIRT1 maintains vascular function against metabolic stress. Chronic SIRT1 supplementation in diabetic mice improves endothelial function by suppressing oxidative stress, positioning SIRT1 as a protective factor against diabetic vascular injury [213]. This endothelial role aligns with the broader function of SIRT1 as a metabolic-stress sensor that couples NAD^+^ availability to vascular homeostasis [214].

In the myocardium, SIRT6 opposes aging-related cardiac decline. Aging is associated with reduced Sirt6 activity together with cardiac hypertrophy and fibrosis; aged hearts show increased mitochondrial DNA damage, shortened telomeres and accumulation of 8-oxo-dG adducts, and Sirt6 mitigates aging-induced cardiomyocyte senescence and cardiac hypertrophy [215]. SIRT7, the nucleolar sirtuin that regulates RNA polymerase I transcription, contributes to myocardial tissue repair by maintaining transforming growth factor-β signalling [216]. In hypertensive hearts, SIRT7 expression falls while cuproptosis rises (decreased FDX1, increased HSP70), and restoring SIRT7 ameliorates myocardial remodelling and cardiac dysfunction through modulation of YAP–ATP7A signalling [217, 218].

In the cardiac mitochondria, SIRT3 is the central regulator. The SIRT3 activator 2-APQC alleviates myocardial hypertrophy and fibrosis by regulating mitochondrial homeostasis, supporting SIRT3 as a therapeutic target in cardiac remodelling [219]. This builds on a long-standing literature establishing mitochondrial SIRT3 as a determinant of cardiac energy metabolism and heart disease [220, 221]. Sirtuins span the full cardiovascular axis: SIRT1 at the endothelium, SIRT6 and SIRT7 in the myocardium, and SIRT3 in the mitochondrial compartment. Sirtuins also sit at the centre of cardiac ageing and fibrosis, where their protective roles make them candidate targets for age-related cardiac remodelling [222, 223]. Whether isoform-specific modulation can achieve cardiovascular benefit without the systemic effects of global NAD^+^ manipulation remains an open translational question.

In myocardial ischemia–reperfusion injury, a complication of reperfusion therapy for acute myocardial infarction, the sirtuin family provides protection against the reactive-oxygen burst, mitochondrial dysfunction and programmed cell death that drive cardiomyocyte loss [224, 225].

### Sirtuins in stem cell regulation and cellular senescence

Sirtuins occupy a central position at the interface between metabolic state, stem cell fate and cellular senescence. Because stem cell exhaustion and cellular senescence are two hallmarks of aging [226, 227], the sirtuin family provides a direct mechanistic link between nutrient sensing and the maintenance of regenerative capacity across tissues. In embryonic stem cells (ESCs), SIRT1 preserves pluripotency by restraining p53 activity. Silencing of the pluripotency factor Oct4 in human ESCs leads to activation of the tumour suppressor p53 and subsequent differentiation, an effect that depends on SIRT1: Oct4 depletion reduces SIRT1 expression, which in turn increases acetylation of p53 at lysine 120 and 164 and derepresses p53-dependent differentiation programs. Ectopic SIRT1 expression in Oct4-silenced cells prevents p53 activation and maintains the undifferentiated state [228]. SIRT1 thus functions as a downstream effector of Oct4, coupling the pluripotency network to the p53 stress axis.

In adult somatic stem cells, SIRT6 maintains stem cell homeostasis through epigenetic restraint of Wnt signalling. In haematopoietic stem cells (HSCs), SIRT6 interacts with LEF1 and deacetylates histone H3 at lysine 56 to suppress transcription of Wnt target genes, a mechanism that preserves HSC quiescence and self-renewal capacity; deletion of Sirt6 impairs HSC quiescence and drives premature exhaustion [229]. A parallel protective role has been documented in human bone marrow mesenchymal stem cells (hBM-MSCs), where knockdown of SIRT6 enables senescence, as evidenced by increased senescence-associated β-galactosidase activity, elevated p16 expression, and reduced proliferative and migratory capacity [230]. These findings position SIRT6 as a general anti-aging and stemness-preserving factor across multiple stem cell compartments.

The nucleolar sirtuin SIRT7 guards genome integrity and opposes cellular senescence by maintaining ribosomal DNA (rDNA) stability. SIRT7 deficiency compromises rDNA integrity and permits senescence induced by rDNA instability [231]. Consistent with this, SIRT7 has been implicated in promoting ribosomal RNA expression, aiding DNA damage repair and regulating chromatin compaction, functions that collectively link nucleolar homeostasis to the aging process [232]. At the family level, sirtuins integrate metabolic cues with the stemness–differentiation balance, with distinct isoforms governing pluripotency, lineage specification and adult stem cell maintenance in a compartment-specific manner [233]. Together, these observations support a model in which nuclear sirtuins (SIRT1, SIRT6, SIRT7) function as guardians of stem cell function and barriers against senescence: SIRT1 through the p53 axis, SIRT6 through epigenetic restraint of Wnt signalling, and SIRT7 through rDNA stability. However, several questions remain unresolved: whether the opposing effects of individual sirtuins on self-renewal versus differentiation reflect distinct substrate specificity or divergent metabolic contexts has not been systematically addressed, and the role of mitochondrial sirtuins (SIRT3–SIRT5) in stem cell metabolism and aging is comparatively understudied.

### Sirtuins in ageing and lifespan regulation

The sirtuin family was discovered through genetic studies linking Sir2 to replicative lifespan extension in yeast under caloric restriction, and aging has remained central to sirtuin biology ever since. In mammals, sirtuins regulate lifespan-associated processes through their shared requirement for NAD^+^, a coenzyme that declines with age and thereby couples metabolic state to the activity of the entire sirtuin network. NAD^+^ is an essential cofactor for sirtuins and poly(ADP-ribose) polymerases (PARPs), functioning both as a redox coenzyme and as a signalling molecule that controls NAD^+^-sensing enzymes [234]. NAD^+^ levels decline in multiple tissues during aging, and this decline is now understood to be driven in part by the NADase CD38, whose expression increases with age. CD38 metabolizes the NAD^+^ precursor NMN, and its age-related upregulation dictates NAD^+^ decline and mitochondrial dysfunction through a SIRT3-dependent mechanism; NAD^+^ levels and mitochondrial function are preserved in old CD38-knockout mice [235].

The NAD^+^–sirtuin axis is a central mediator of caloric restriction, the most robust longevity intervention in model organisms. Because sirtuins are NAD^+^-dependent, caloric restriction is proposed to activate sirtuins through elevated NAD^+^ availability, and pharmacological strategies to raise NAD^+^, including the precursors nicotinamide mononucleotide (NMN) and nicotinamide riboside (NR), have shown therapeutic potential in vivo across metabolic and age-related contexts [236]. The NAD^+^–sirtuin connection therefore links nutrient availability to organismal aging through a conserved, druggable pathway [237]. Sirtuins also mediate the pancreatic effects of caloric restriction, regulating β-cell function, metabolism and longevity [238].

Among the seven mammalian sirtuins, SIRT1 and SIRT6 are the most consistently implicated in aging-related diseases, functioning in metabolic adaptation, genome maintenance and inflammation, and their modulation has been proposed as a therapeutic strategy across cardiovascular, neurodegenerative and metabolic disorders of aging [239]. Sex-specific effects are emerging: hepatocyte-specific Sirt6 knockout accelerates female-specific aging through SULT1E1-mediated estrogen depletion [240]. A recent advance has identified an endogenous mechanism of sirtuin activation. Lithocholic acid (LCA), a bile acid that accumulates in mammals during caloric restriction, binds the protein TULP3 to activate sirtuins and AMPK, thereby slowing aging in animal models [241]. This finding extends the NAD^+^–sirtuin–longevity framework by identifying a caloric-restriction-derived metabolite that directly engages the sirtuin network, suggesting that the anti-aging effects of caloric restriction are mediated by both NAD^+^ availability and specific endogenous activators.

These findings establish the sirtuin family as a conserved nexus linking NAD^+^ metabolism, caloric restriction and the aging process. However, several questions remain unresolved: whether NAD^+^ decline is a cause or a consequence of aging has not been definitively established, and the extent to which NAD^+^-boosting interventions translate from rodent models to human aging remains uncertain given heterogeneous clinical outcomes. The identification of endogenous activators such as LCA raises the further question of whether physiological sirtuin activation, rather than pharmacological NAD^+^ elevation, represents a more tractable route to modulating human healthspan.

## Therapeutic targeting of sirtuins

The distributed, compartmentalized architecture of the sirtuin network presents both opportunities and obstacles for therapeutic intervention: isoform selectivity, compartment-specific drug exposure, and the risk of disrupting network-level coordination must all be navigated. This section evaluates current strategies against this biological complexity.

### Direct pharmacological modulation

Direct pharmacological modulation refers to small molecules that bind sirtuin proteins and alter catalytic activity through allosteric or competitive mechanisms. In contrast to indirect NAD^+^-based approaches, these strategies aim to achieve isoform selectivity at the level of enzyme engagement. However, selectivity is multidimensional, involving isoform preference, substrate bias, and compartment-specific exposure. SIRT1 has been among the most intensively explored sirtuin isoforms and serves as a useful paradigm for both the promise and limitations of direct sirtuin targeting. Early enthusiasm was driven by the discovery that resveratrol [242], a naturally occurring polyphenol, functions as an allosteric activator of SIRT1 [243]. Resveratrol enhances SIRT1-mediated deacetylation and engages the SIRT1-AMPK-PGC-1α axis, improving mitochondrial function, oxidative stress resistance and metabolic homeostasis across numerous preclinical models. However, clinical translation has been challenging. In a Phase I trial of micronized resveratrol (SRT501) in patients with hepatic metastases, 5 g/day produced dose-limiting gastrointestinal intolerance (nausea, vomiting, diarrhea) [244]; at ≤ 1 g/day, resveratrol is generally well tolerated across multiple disease settings. Although modest improvements in inflammatory and metabolic biomarkers have been reported in some trials, disease-modifying effects have been weak or absent. Concerns about renal toxicity derive from preclinical rodent studies at suprapharmacological doses [245] and have not been prominent in human data. The Sirtris Pharmaceuticals program, which advanced SRT501 and second-generation SIRT1 activators (SRT1720, SRT2104) into Phase II trials, was ultimately discontinued after SRT2104 failed to meet primary efficacy endpoints in chronic obstructive pulmonary disease and inflammatory disease [246].

A conceptual advance has recently emerged with the development of NAD^+^-independent SIRT1 activators based on an (E)−2’-demethylsulfonic acid scaffold [247]. Unlike classical STACs, these compounds directly participate as acetyl acceptors in the deacetylation reaction, enabling SIRT1 activation under NAD^+^-depleted conditions characteristic of ageing and metabolic disease. Although highly selective and efficacious in preclinical models, their pharmacokinetic optimization and translational relevance remain to be established. Two decades of SIRT1-directed drug discovery illustrate an important lesson: chemical tractability alone is insufficient to overcome the biological complexity of sirtuin regulation in vivo.

Beyond SIRT1, increasing efforts have focused on targeting other sirtuin family members, revealing pronounced isoform-specific opportunities and limitations. SIRT2 inhibitors, including AGK2, AK-7, display biochemical selectivity and central nervous system penetration [248], enabling extensive preclinical exploration in neurodegenerative disease, cancer and inflammatory models. However, no SIRT2-directed agents have yet progressed to clinical testing, reflecting ongoing uncertainties regarding therapeutic windows and tissue-specific effects. Recent identification of S-ketamine as a stereospecific, NAD^+^-independent SIRT2 modulator highlights alternative regulatory strategies but also underscores the early stage of translational development [249].

SIRT3, the primary mitochondrial deacetylase, is a promising therapeutic node among sirtuins. Natural activators such as honokiol, polydatin [250] and melatonin [251] enhance mitochondrial antioxidant capacity and bioenergetic efficiency, while structure-guided efforts have yielded synthetic allosteric activators with improved potency and pharmacokinetics. A notable example is SKLB-11A [252], which activates SIRT3 through a distinct conformational mechanism and confers robust cardioprotection in preclinical models. Despite these advances, SIRT3-directed strategies remain confined to preclinical stages.

SIRT5 stands out as the isoform with relatively mature chemical toolbox. Highly selective substrate-competitive inhibitors, including ε-N-thioglutaryl-lysine derivatives and pyrazolone-based compounds, have enabled detailed dissection of SIRT5-dependent metabolic pathways [253]. However, the lack of clearly defined clinical indications and limited in vivo validation have thus far constrained translational progress. By contrast, SIRT6 is a compelling nuclear target, with allosteric activators such as MDL-800 [254], MDL-801 [254] and MDL-811 [255] showing strong efficacy in models of cardiometabolic, inflammatory and ischemic disease. An orally available SIRT6-modulating compound (IMU-856) has entered early-phase clinical testing, marking a potential step forward for isoform-selective sirtuin therapeutics [256].

By contrast, SIRT7 inhibition is supported by more explicit chemical evidence, although still at an early translational stage. Structure-based modelling and virtual screening have yielded candidate inhibitors with preferential affinity for SIRT7, proposed as starting points for liver cancer therapy. Complementary approaches using cyclic tripeptide inhibitors demonstrate low-micromolar inhibition of human SIRT7, establishing proof-of-principle pharmacophores but with inherent peptide-associated limitations [257]. In disease models, SIRT7 inhibition has also been achieved using bioactive natural products, such as Oroxylin A, to reprogramme hepatic stellate cell fate in liver fibrosis [258]. Nonetheless, the field remains constrained by limited isoform selectivity profiling, insufficient target-engagement validation, and a lack of standardized pharmacodynamic biomarkers, which underscores the early translational status of SIRT7-directed therapeutics. Together, these efforts highlight that progress in isoform-selective sirtuin pharmacology is constrained less by chemical feasibility than by the need for validated biomarkers, tissue-specific target engagement and disease-contextualized indication selection. Direct pharmacological and chemical tools targeting individual sirtuins are summarized in Table 1.

**Table 1:** Direct modulators and chemical tools targeting members of the sirtuin family

**SIRT family member**	**Regulatory factor**	**Modality/mechanism**	**Effect on SIRT**	**Clinical status**
SIRT1	Resveratrol [242–245]	Allosteric activator; links SIRT1 to AMPK–PGC-1α signalling	↑	Phase I–II clinical trials (NCT01354977); heterogeneous results; well tolerated at ≤ 1 g/d, higher doses (~5 g/d) associated with adverse effects including renal injury, contributing to trial discontinuation in some settings
SIRT1	SRT1720; SRT2104 [246]	Second-generation SIRT1-activating compounds (STACs; binds SIRT1 N‑terminal regulatory domain	↑	SRT1720: Preclinical (metabolic and neurodegenerative models) SRT2104: Phase II completed (T2DM, psoriasis; NCT01154101); no late‑stage development
SIRT1	((E)−2′-demethylsulfonic acid scaffold) [247]	NAD^+^‑independent activator; acts as acetyl acceptor	↑	Preclinical; validated under NAD^+^‑depleted conditions
SIRT2	AGK2; AK-7 [248, 259, 260]	Selective SIRT2 inhibitors with central nervous system penetrance	↓	Preclinical (neurodegenerative, cancer, inflammatory models)
SIRT2	S-ketamine [249]	Stereospecific, NAD^+^‑independent SIRT2 modulator; enhances SIRT2–p65 interaction	↑	Preclinical; identified via repurposing for depression
SIRT3	Honokiol; Polydatin; Melatonin [250, 251, 261]	Natural product SIRT3 activators; enhance mitochondrial antioxidant capacity	↑	Preclinical models
SIRT3	SKLB-11A [252]	Synthetic allosteric SIRT3 activator with a distinct conformational mechanism	↑	Preclinical models
SIRT5	ε-N-thioglutaryl-lysine derivatives; pyrazolone-based compounds [253]	Substrate-competitive SIRT5 inhibitors	↓	Preclinical; chemical probes for SIRT5
SIRT6	MDL-800; MDL-801; MDL-811 [254, 255]	Allosteric SIRT6 activators	↑	Preclinical (cardiometabolic, inflammatory, ischemic models)
SIRT6	IMU-856 [256]	Orally available SIRT6-modulating compound	↑	Phase I completed (coeliac disease; ACTRN12620000901909)
SIRT7	Virtual-screening-derived candidate inhibitors [262]	Structure-based SIRT7 inhibitors	↓	Preclinical; proposed for liver cancer
SIRT7	Cyclic tripeptide inhibitors [257]	Low-micromolar SIRT7-selective inhibitors	↓	Preclinical; proof-of-principle pharmacophores
SIRT7	Oroxylin A [258]	Natural SIRT7 inhibitor; induces HSC senescence	↓	Preclinical (anti-fibrotic in liver fibrosis)
SIRT2	SirReal-based PROTACs [263–265]	Cereblon-recruiting degraders enabling selective SIRT2 protein degradation	↓ (protein degradation)	Most mature examples of sirtuin-directed PROTAC strategies
SIRT6	SIRT6 degraders (PROTAC technology) [266]	Potent degraders suppress hepatocellular carcinoma growth	↓ (protein degradation)	Hepatocellular carcinoma models

Symbol footnotes: ↑, increased activity:↓, decreased activity

### Indirect strategies

In contrast to direct small-molecule modulators that bind sirtuins at the catalytic or allosteric level, indirect strategies alter sirtuin activity by reshaping the metabolic or signalling environments in which these enzymes operate. Given that all seven mammalian sirtuins are obligate NAD^+^-dependent deacylases, interventions that influence NAD^+^ availability, redox balance, nutrient sensing or transcriptional control can modulate sirtuin function across a wide range of tissues and disease states. Such approaches often lack isoform selectivity but may exert broader physiological impact by engaging the family-level regulatory logic described in earlier sections of this review.

Restoration of intracellular NAD^+^ levels represents the most extensively explored indirect approach. Precursors such as nicotinamide riboside (NR) and nicotinamide mononucleotide (NMN) increase cellular NAD^+^ pools and have demonstrated metabolic and cardiovascular benefits in experimental models. However, NAD^+^ elevation does not activate a single sirtuin in isolation. Instead, it enhances the activity of multiple compartmentalized sirtuins in a manner determined by tissue distribution, subcellular NAD^+^ pools and metabolic demand. As discussed above, nuclear, cytosolic and mitochondrial NAD^+^ pools are only partially equilibrated; therefore, global NAD^+^ boosting may preferentially engage certain isoforms depending on disease context.

This principle is illustrated in studies often described as “SIRT7 activation.” In most cases, SIRT7 is not directly targeted by a selective agonist. Rather, NAD^+^ precursor supplementation or metabolic reprogramming increases SIRT7 activity as a downstream consequence of improved NAD^+^ availability or altered transcriptional regulation. For example, nicotinamide riboside supplementation phenocopies Sirt7 overexpression in pulmonary hypertension models, while hypoxia-induced NAD^+^ production via PYCR1 enhances Sirt7 deacetylase activity within ELK4-dependent programmes [267]. In other contexts, increased SIRT7 expression, such as during ketogenic diet exposure or GLSP-mediated signalling, has been interpreted as functional activation [268, 269]. These findings indicate that most in vivo “SIRT7 activation” reflects metabolic licensing rather than direct catalytic stimulation.

Beyond NAD^+^ precursors, pharmacological agents that alter cellular energy balance can indirectly regulate sirtuin activity. Metformin is a prototypical example. By inhibiting mitochondrial complex I, metformin lowers ATP levels, activates AMPK and suppresses mTORC1 signalling. This shift in energy state enhances the AMPK–SIRT1 axis and modulates downstream pathways including PGC-1α, lipid metabolism and inflammatory signalling [270, 271]. The clinical benefits of metformin cannot be attributed solely to SIRT1 activation; rather, SIRT1 participates as one component of a broader metabolic network.

This systems-level engagement is conceptually consistent with the compartmentalized regulatory framework established above. Energy-sensing interventions alter glycolytic flux, fatty acid oxidation and mitochondrial redox state, thereby reshaping the acylation landscape on which sirtuins act. In this context, sirtuins function less as primary drug targets and more as interpreters of metabolic reconfiguration. Indirect and systems-level regulatory mechanisms are outlined in Table 2.

**Table 2:** Indirect and systems-level mechanisms regulating sirtuin activity in physiological and disease contexts

SIRT family member	Regulatory factor	Regulation type/mechanism	Effect on SIRT	Disease or experimental context
SIRT1	Metformin [270, 271]	Indirect engagement of the AMPK–SIRT1 axis through cellular energy balance; suppresses of mTORC1–SREBP signalling	↑ (indirect)	Cardiometabolic benefits supported by large clinical datasets
SIRT7	Nicotinamide riboside (NR) [272]	NAD^+^ precursor; described as activating Sirt7 in vivo via increased NAD^+^ availability	↑ (indirect)	Pulmonary hypertension models
SIRT7	Hypoxia-induced NAD^+^ production via PYCR1 [267]	Enhanced NAD^+^ availability increases Sirt7 deacetylase activity within an ELK4-dependent transcriptional programme	↑ (indirect)	Hypoxia-associated tumour growth
SIRT7	β-hydroxybutyrate-mediated promoter acetylation [268]	Promotes SIRT7 promoter acetylation → increased SIRT7 expression	↑ (expression upregulation)	Ketogenic diet exposure
SIRT7	GLSP-driven enhancement of Sirt7–Keap1–Nrf2 signalling [269]	Transcriptional upregulation of SIRT7; enhances SIRT7–Keap1–Nrf2 signalling	↑ (signalling enhancement)	Vascular ageing models

Symbol footnotes: ↑, increased activity: ↓, decreased activity

### Emerging therapeutic modalities

Proteolysis-targeting chimeras (PROTACs) are a versatile modality to modulate sirtuin function by inducing selective protein degradation rather than enzymatic inhibition, offering a complementary strategy to conventional small-molecule inhibitors. To date, the most mature examples are centred on SIRT2, where sirtuin-rearranging ligands (SirReals) have been repurposed into PROTAC architectures capable of driving efficient and isoform-selective SIRT2 degradation via cereblon recruitment [263, 264]. These degraders demonstrated that removal of the SIRT2 protein can yield biological outcomes distinct from catalytic inhibition, including differential effects on microtubule acetylation and cellular morphology [265]. Subsequent studies further extended this concept by developing next-generation SIRT2-targeted PROTACs with enhanced antiproliferative activity in cancer models, supporting the therapeutic promise of sirtuin degradation strategies [122, 273]. Beyond SIRT2, SIRT6 has also been targeted using PROTAC technology. One such degrader, SZU-B6, induced near-complete SIRT6 degradation, impaired DNA damage repair and enhanced radiosensitization in hepatocellular carcinoma cell lines, and exhibited synergistic antitumor activity with sorafenib or irradiation in xenograft models [266]. These findings highlight the feasibility of extending protein-degradation approaches to other nuclear sirtuins implicated in cancer and metabolism. More broadly, comprehensive reviews of HDAC- and sirtuin-directed PROTACs underscore that epigenetic enzymes may be well suited for degradation-based pharmacology, owing to their scaffolded protein–protein interactions and scaffold-dependent non-enzymatic functions [274, 275].

While direct PROTACs against SIRT7 have not yet been reported, several mechanistic studies emphasize that selective elimination of individual sirtuins can produce phenotypes not achievable by inhibition alone, providing a conceptual framework for future SIRT7 degrader development. The growing chemical biology toolbox for sirtuins (including activity-based probes to assess functional engagement and degradation outcomes [276]) is expected to facilitate this transition. Current evidence positions PROTAC-mediated sirtuin degradation as an advancing strategy, with SIRT2 and SIRT6 serving as precedents and SIRT7 representing a plausible next direction for targeted protein degradation approaches.

Current therapeutic efforts targeting sirtuins illustrate both the tractability and the limitations of this enzyme family as drug targets. While small-molecule modulators, NAD^+^ precursors and emerging degradation strategies have demonstrated preclinical efficacy and acceptable safety profiles, their clinical performance has been heterogeneous and frequently dependent on patient selection and disease stage. These outcomes point to the need for improved isoform selectivity, clearer pharmacodynamic biomarkers and a more nuanced understanding of how disease-specific metabolic states shape sirtuin function in vivo. Rather than representing a failure of the sirtuin concept, these challenges highlight the transitional nature of the field and set the stage for a broader, family-wide reassessment of sirtuin biology and therapeutic strategy. Key challenges for PROTAC-based sirtuin modulation include achieving tissue-selective degradation, minimizing off-target proteostasis perturbation and establishing pharmacodynamic biomarkers that distinguish degradation from inhibition.

A recent synthesis of context-dependent sirtuin pharmacology in cancer highlights metabolic-epigenetic switches and biomarker stratification as the basis for precision therapeutic targeting [277].

A central principle emerging from this analysis is that therapeutic selectivity must be defined at the level of subcellular compartment and isoform, not merely at the level of the sirtuin family. Sirtuin function is compartmentalized, so interventions that raise NAD^+^ globally (such as NAD^+^ precursors) necessarily engage all seven isoforms across every compartment. This broad engagement likely underlies their heterogeneous clinical outcomes. Direct, isoform-selective modulators and PROTAC-mediated degradation restrict the intervention to a single isoform, matching the therapeutic strategy to the compartment-specific disease mechanism. A second principle is that the direction of intervention, whether activation, inhibition, or degradation, should be matched to disease context. The same sirtuin can be protective in one tissue and pathogenic in another, so a fixed agonist or inhibitor is unlikely to be broadly useful. Degradation, by contrast, removes the target entirely and can recapitulate phenotypes that inhibition alone cannot achieve, as illustrated by SIRT2-directed PROTACs. These principles carry three implications for developing more selective sirtuin-targeted interventions. First, prioritize isoform-selective over family-wide modulation; second, match the modality (activation, inhibition, degradation) to the tissue- and disease-specific role of the target isoform; and third, incorporate pharmacodynamic biomarkers that report isoform-specific target engagement rather than global NAD^+^ status.

## Conclusions and future perspectives

Sirtuins have evolved as a family of NAD^+^-dependent deacylases that collectively function as a distributed metabolic-sensing network, rather than as isolated enzymatic regulators. Across subcellular compartments, SIRT1–SIRT7 translate fluctuations in nutrient availability, redox balance and cellular stress into coordinated transcriptional, epigenetic and post-translational responses. This compartmentalized organization enables sirtuins to regulate core processes such as glycolysis, fatty acid oxidation, mitochondrial quality control, inflammation and immune tolerance with a high degree of context sensitivity. Viewing sirtuins through a family-wide lens therefore helps reconcile their apparent pleiotropy and provides a conceptual framework in which metabolic state, spatial localization and substrate accessibility jointly shape biological outcomes.

A recurring theme emerging from this review is that many seemingly contradictory roles attributed to individual sirtuins reflect differences in metabolic context, tissue identity and temporal dynamics rather than intrinsic functional inconsistency. The same sirtuin can exert protective or deleterious effects depending on NAD^+^ pool availability, disease stage and cellular lineage. This tissue- and disease-stage dependence is particularly evident in cancer, inflammation and metabolic disease, where sirtuin-mediated regulation can either restrain maladaptive metabolic reprogramming or be co-opted to support pathological states. Recognizing sirtuins as context-sensitive modulators, rather than fixed oncoproteins or tumour suppressors, is therefore important for accurate interpretation of experimental data and for rational therapeutic targeting.

Despite substantial progress, important gaps remain in our understanding of sirtuin biology. First, the extent to which different sirtuin isoforms functionally cooperate or compensate for one another across subcellular compartments is poorly defined. Second, the mechanisms governing compartment-specific NAD^+^ pool dynamics and their selective impact on individual sirtuins are still incompletely understood. Third, non-canonical functions of sirtuins, including scaffold roles and non-enzymatic activities revealed by protein degradation approaches, may be underappreciated. Finally, several family members, most notably SIRT4 and SIRT7, remain relatively understudied in immune regulation and human disease, representing promising opportunities for future investigation.

From a translational perspective, this review highlights why early attempts to therapeutically target sirtuins have yielded mixed clinical outcomes. Strategies focused on single isoform activation or inhibition often fail to account for the interconnected, family-level regulatory logic of sirtuin signalling. Emerging approaches offer new opportunities to overcome these limitations, including isoform-selective modulators, NAD^+^-independent activators, PROTAC-mediated degradation and combination therapies integrating metabolic and immune interventions. Successful clinical translation is likely to require both improved chemical selectivity and robust pharmacodynamic biomarkers and patient stratification based on metabolic and disease context.

In conclusion, the sirtuin family represents a key regulatory axis at the intersection of metabolism, epigenetics and immune homeostasis. By framing sirtuins as a coordinated, compartmentalised network rather than as individual drug targets, this review provides a unifying conceptual foundation for interpreting the expanding literature and for guiding future therapeutic development. Advances in understanding sirtuin cross-talk, NAD^+^ compartmentalization and isoform-specific vulnerabilities have the potential to transform sirtuin biology from a field characterized by complexity and controversy into one driven by greater mechanistic clarity and translational precision. Realizing this potential will depend on integrating mechanistic insights with rigorous in vivo validation and carefully designed clinical studies.

## Data Availability

Not applicable.

## References

[1] Gottlieb S, Esposito RE. A new role for a yeast transcriptional silencer gene, SIR2, in regulation of recombination in ribosomal DNA. Cell. 1989;56:771–6. 10.1016/0092-8674(89)90681-8. 2647300

[2] Johnson LM, Kayne PS, Kahn ES, Grunstein M. Genetic evidence for an interaction between SIR3 and histone H4 in the repression of the silent mating loci in Saccharomyces cerevisiae. Proc Natl Acad Sci U S A. 1990;87:6286–90. 10.1073/pnas.87.16.6286. PMC54518 2201024

[3] Imai S, Armstrong CM, Kaeberlein M, Guarente L. Transcriptional silencing and longevity protein Sir2 is an NAD-dependent histone deacetylase. Nature. 2000;403:795–800. 10.1038/35001622. 10693811

[4] Baur JA, Pearson KJ, Price NL, Jamieson HA, Lerin C, Kalra A, et al. Resveratrol improves health and survival of mice on a high-calorie diet. Nature. 2006;444:337–42. 10.1038/nature05354. PMC4990206 17086191

[5] Cantó C, Houtkooper RH, Pirinen E, Youn DY, Oosterveer MH, Cen Y, et al. The NAD(+) precursor nicotinamide riboside enhances oxidative metabolism and protects against high-fat diet-induced obesity. Cell Metab. 2012;15:838–47. 10.1016/j.cmet.2012.04.022. PMC3616313 22682224

[6] Chua KF, Mostoslavsky R, Lombard DB, Pang WW, Saito S, Franco S, et al. Mammalian SIRT1 limits replicative life span in response to chronic genotoxic stress. Cell Metab. 2005;2:67–76. 10.1016/j.cmet.2005.06.007. 16054100

[7] Vaziri H, Dessain SK, Eaton EN, Imai SI, Frye RA, Pandita TK, et al. hSIR2(SIRT1) functions as an NAD-dependent p53 deacetylase. Cell. 2001;107:149–59. 10.1016/s0092-8674(01)00527-x. 11672523

[8] Brunet A, Sweeney LB, Sturgill JF, Chua KF, Greer PL, Lin Y, et al. Stress-dependent regulation of FOXO transcription factors by the SIRT1 deacetylase. Science. 2004;303:2011–5. 10.1126/science.1094637. 14976264

[9] Rodgers JT, Lerin C, Haas W, Gygi SP, Spiegelman BM, Puigserver P. Nutrient control of glucose homeostasis through a complex of PGC-1alpha and SIRT1. Nature. 2005;434:113–8. 10.1038/nature03354. 15744310

[10] Zhao Q, Jing Y, Jiang X, Zhang X, Liu F, Huang H, et al. SIRT5 safeguards against primate skeletal muscle ageing via desuccinylation of TBK1. Nat Metab. 2025;7:556–73. 10.1038/s42255-025-01235-8. 40087407

[11] Wang H, Chen Y, Wang Y, Tao E, Tan J, Liu Q, et al. Sirtuin5 protects colorectal cancer from DNA damage by keeping nucleotide availability. Nat Commun. 2022;13:6121. 10.1038/s41467-022-33903-8. PMC9576705 36253417

[12] Feldman JL, Baeza J, Denu JM. Activation of the protein deacetylase SIRT6 by long-chain fatty acids and widespread deacylation by mammalian sirtuins. J Biol Chem. 2013;288:31350–6. 10.1074/jbc.C113.511261. PMC3829447 24052263

[13] Cen Y, Sauve AA. Transition state of ADP-ribosylation of acetyllysine catalyzed by Archaeoglobus fulgidus Sir2 determined by kinetic isotope effects and computational approaches. J Am Chem Soc. 2010;132:12286–98. 10.1021/ja910342d. PMC2952425 20718419

[14] Hirschey MD, Shimazu T, Goetzman E, Jing E, Schwer B, Lombard DB, et al. SIRT3 regulates mitochondrial fatty-acid oxidation by reversible enzyme deacetylation. Nature. 2010;464:121–5. 10.1038/nature08778. PMC2841477 20203611

[15] Crampton A, Chang F, Pappas DL, Frisch RL, Weinreich M. An ARS element inhibits DNA replication through a SIR2-dependent mechanism. Mol Cell. 2008;30:156–66. 10.1016/j.molcel.2008.02.019. 18439895

[16] Yi X, Wang H, Yang Y, Wang H, Zhang H, Guo S, et al. SIRT7 orchestrates melanoma progression by simultaneously promoting cell survival and immune evasion via UPR activation. Signal Transduct Target Ther. 2023;8:107. 10.1038/s41392-023-01314-w. PMC10015075 36918544

[17] Mao Z, Hine C, Tian X, Van Meter M, Au M, Vaidya A, et al. SIRT6 promotes DNA repair under stress by activating PARP1. Science. 2011;332:1443–6. 10.1126/science.1202723. PMC5472447 21680843

[18] Wu Q, Zhang T, Chen H, Yu X, Lv J, Liu Y, et al. The sirtuin family in health and disease. Signal Transduct Target Ther. 2022;7:402. 10.1038/s41392-022-01257-8. PMC9797940 36581622

[19] Navas LE, Carnero A. NAD+ metabolism, stemness, the immune response, and cancer. Signal Transduct Target Ther. 2021;6:2. 10.1038/s41392-020-00354-w. PMC7775471 33384409

[20] Anderson RM, Bitterman KJ, Wood JG, Medvedik O, Sinclair DA. Nicotinamide and PNC1 govern lifespan extension by calorie restriction in Saccharomyces cerevisiae. Nature. 2003;423:181–5. 10.1038/nature01578. PMC4802858 12736687

[21] Chen F, Xu W, Tang M, Tian Y, Shu Y, He X, et al. hnRNPA2B1 deacetylation by SIRT6 restrains local transcription and safeguards genome stability. Cell Death Differ. 2025;32:382–96. 10.1038/s41418-024-01412-4. PMC11893882 39511404

[22] Chen S, Blank MF, Iyer A, Huang B, Wang L, Grummt I, et al. SIRT7-dependent deacetylation of the U3–55k protein controls pre-rRNA processing. Nat Commun. 2016;7:10734. 10.1038/ncomms10734. PMC4754350 26867678

[23] Jin L, Geng L, Ying L, Shu L, Ye K, Yang R, et al. FGF21-Sirtuin 3 Axis Confers the Protective Effects of Exercise Against Diabetic Cardiomyopathy by Governing Mitochondrial Integrity. Circulation. 2022;146:1537–57. 10.1161/CIRCULATIONAHA.122.059631. 36134579

[24] Jeong SM, Xiao C, Finley LWS, Lahusen T, Souza AL, Pierce K, et al. SIRT4 has tumor-suppressive activity and regulates the cellular metabolic response to DNA damage by inhibiting mitochondrial glutamine metabolism. Cancer Cell. 2013;23:450–63. 10.1016/j.ccr.2013.02.024. PMC3650305 23562301

[25] Du J, Zhou Y, Su X, Yu JJ, Khan S, Jiang H, et al. Sirt5 is a NAD-dependent protein lysine demalonylase and desuccinylase. Science. 2011;334:806–9. 10.1126/science.1207861. PMC3217313 22076378

[26] Park S, Ozden O, Liu G, Song HY, Zhu Y, Yan Y, et al. SIRT2-mediated deacetylation and tetramerization of pyruvate kinase directs glycolysis and tumor growth. Cancer Res. 2016;76:3802–12. 10.1158/0008-5472.CAN-15-2498. PMC4930699 27197174

[27] Baur JA, Ungvari Z, Minor RK, Le Couteur DG, de Cabo R. Are sirtuins viable targets for improving healthspan and lifespan. Nat Rev Drug Discov. 2012;11:443–61. 10.1038/nrd3738. PMC4684642 22653216

[28] Nikiforov A, Kulikova V, Ziegler M. The human NAD metabolome: Functions, metabolism and compartmentalization. Crit Rev Biochem Mol Biol. 2015;50:284–97. 10.3109/10409238.2015.1028612. PMC4673589 25837229

[29] Aguilar-Arnal L, Ranjit S, Stringari C, Orozco-Solis R, Gratton E, Sassone-Corsi P. Spatial dynamics of SIRT1 and the subnuclear distribution of NADH species. Proc Natl Acad Sci U S A. 2016;113:12715–20. 10.1073/pnas.1609227113. PMC5111721 27791113

[30] Tang C, Livingston MJ, Liu Z, Dong Z. Autophagy in kidney homeostasis and disease. Nat Rev Nephrol. 2020;16:489–508. 10.1038/s41581-020-0309-2. PMC7868042 32704047

[31] Grootaert MOJ, Bennett MR. Sirtuins in atherosclerosis: guardians of healthspan and therapeutic targets. Nat Rev Cardiol. 2022;19:668–83. 10.1038/s41569-022-00685-x. 35354967

[32] Wang Y, Wang H, Xu J, Tan J, Fu L, Wang J, et al. Sirtuin5 contributes to colorectal carcinogenesis by enhancing glutaminolysis in a deglutarylation-dependent manner. Nat Commun. 2018;9:545. 10.1038/s41467-018-02951-4. PMC5803207 29416026

[33] Haigis MC, Mostoslavsky R, Haigis KM, Fahie K, Christodoulou DC, Murphy AJ, et al. SIRT4 inhibits glutamate dehydrogenase and opposes the effects of calorie restriction in pancreatic beta cells. Cell. 2006;126:941–54. 10.1016/j.cell.2006.06.057. 16959573

[34] Verdin E. NAD⁺ in aging, metabolism, and neurodegeneration. Science. 2015;350:1208–13. 10.1126/science.aac4854. 26785480

[35] Mendes KL, de Farias LD, Santos SHS. Nuclear sirtuins and inflammatory signaling pathways. Cytokine Growth Factor Rev. 2017;38:98–105. 10.1016/j.cytogfr.2017.11.001. 29132743

[36] Gomes P, Outeiro TF, Cavadas C. Emerging role of sirtuin 2 in the regulation of mammalian metabolism. Trends Pharmacol Sci. 2015;36:756–68. 10.1016/j.tips.2015.08.001. 26538315

[37] Longo VD, Kennedy BK. Sirtuins in aging and age-related disease. Cell. 2006;126:257–68. 10.1016/j.cell.2006.07.002. 16873059

[38] Morigi M, Perico L, Benigni A. Sirtuins in Renal Health and Disease. J Am Soc Nephrol. 2018;29:1799–809. 10.1681/ASN.2017111218. PMC6050939 29712732

[39] Cambronne XA, Stewart ML, Kim D, Jones-Brunette AM, Morgan RK, Farrens DL, et al. Biosensor reveals multiple sources for mitochondrial NAD⁺. Science. 2016;352:1474–7. 10.1126/science.aad5168. PMC6530784 27313049

[40] Nogueiras R, Habegger KM, Chaudhary N, Finan B, Banks AS, Dietrich MO, et al. Sirtuin 1 and sirtuin 3: physiological modulators of metabolism. Physiol Rev. 2012;92:1479–514. 10.1152/physrev.00022.2011. PMC3746174 22811431

[41] Gong Y, Tang N, Liu P, Sun Y, Lu S, Liu W, et al. Newcastle disease virus degrades SIRT3 via PINK1-PRKN-dependent mitophagy to reprogram energy metabolism in infected cells. Autophagy. 2022;18:1503–21. 10.1080/15548627.2021.1990515. PMC9298456 34720029

[42] Wang M, Lin H. Understanding the Function of Mammalian Sirtuins and Protein Lysine Acylation. Annu Rev Biochem. 2021;90:245–85. 10.1146/annurev-biochem-082520-125411. 33848425

[43] Zu Y, Liu L, Lee MYK, Xu C, Liang Y, Man RY, et al. SIRT1 promotes proliferation and prevents senescence through targeting LKB1 in primary porcine aortic endothelial cells. Circ Res. 2010;106:1384–93. 10.1161/CIRCRESAHA.109.215483. 20203304

[44] Fu G, Chen S, Liang L, Li X, Tang P, Rao X, et al. SIRT1 inhibitors mitigate radiation-induced GI syndrome by enhancing intestinal-stem-cell survival. Cancer Lett. 2021;501:20–30. 10.1016/j.canlet.2020.12.034. 33359449

[45] Fiorentino F, Fabbrizi E, Mai A, Rotili D. Activation and inhibition of sirtuins: From bench to bedside. Med Res Rev. 2025;45:484–560. 10.1002/med.22076. PMC11796339 39215785

[46] Tatone C, Di Emidio G, Barbonetti A, Carta G, Luciano AM, Falone S, et al. Sirtuins in gamete biology and reproductive physiology: emerging roles and therapeutic potential in female and male infertility. Hum Reprod Update. 2018;24:267–89. 10.1093/humupd/dmy003. 29447380

[47] Zhu L, Huang M, He L, Rong Z. Sirt1 attenuates necrotizing enterocolitis via Hif-1α deacetylation-mediated suppression of Bnip3-Dependent mitophagy. Free Radic Biol Med. 2025;241:150–60. 10.1016/j.freeradbiomed.2025.09.020. 40947034

[48] Huang L, Sun H, Song F, Cao Z, Jiang X, Zhang L, et al. SIRT6 overexpression inhibits cementogenesis by suppressing glucose transporter 1. J Cell Physiol. 2019;234:4005–14. 10.1002/jcp.27213. 30256390

[49] Hou KL, Lin SK, Chao LH, Hsiang-Hua Lai E, Chang CC, Shun CT, et al. Sirtuin 6 suppresses hypoxia-induced inflammatory response in human osteoblasts via inhibition of reactive oxygen species production and glycolysis-A therapeutic implication in inflammatory bone resorption. BioFactors. 2017;43:170–80. 10.1002/biof.1320. 27534902

[50] Bhardwaj A, Das S. SIRT6 deacetylates PKM2 to suppress its nuclear localization and oncogenic functions. Proc Natl Acad Sci U S A. 2016;113:E538-547. 10.1073/pnas.1520045113. PMC4747762 26787900

[51] Lu H, Lin J, Xu C, Sun M, Zuo K, Zhang X, et al. Cyclosporine modulates neutrophil functions via the SIRT6-HIF-1α-glycolysis axis to alleviate severe ulcerative colitis. Clin Transl Med. 2021;11:e334. 10.1002/ctm2.334. PMC7882115 33634990

[52] Seo K, Park J, Heo J, Jing K, Han J, Min K, et al. SIRT2 regulates tumour hypoxia response by promoting HIF-1α hydroxylation. Oncogene. 2015;34:1354–62. 10.1038/onc.2014.76. 24681946

[53] Dikalova AE, Pandey A, Xiao L, Arslanbaeva L, Sidorova T, Lopez MG, et al. Mitochondrial deacetylase Sirt3 reduces vascular dysfunction and hypertension while Sirt3 depletion in essential hypertension is linked to vascular inflammation and oxidative stress. Circ Res. 2020;126:439–52. 10.1161/CIRCRESAHA.119.315767. PMC7035170 31852393

[54] Barroso E, Rodríguez-Rodríguez R, Zarei M, Pizarro-Degado J, Planavila A, Palomer X, et al. SIRT3 deficiency exacerbates fatty liver by attenuating the HIF1α-LIPIN 1 pathway and increasing CD36 through Nrf2. Cell Commun Signal. 2020;18:147. 10.1186/s12964-020-00640-8. PMC7488148 32912335

[55] Zhang X, Di Y, Wang Y, Qin J, Ye L, Wen X, et al. SIRT5-mediated desuccinylation of PPA2 enhances HIF-1alpha-dependent adaptation to hypoxic stress and colorectal cancer metastasis. EMBO J. 2025;44:2514–40. 10.1038/s44318-025-00416-1. PMC12048626 40164945

[56] Lian X, Dai M, Xu X, Zhao H, Li S, Liu X, et al. Ailanthone targets mitophagy-loaded SIRT4 to facilitate the deacetylation of HIF1α, triggering oxidative stress for gastric carcinoma treatment. Phytomedicine. 2025;148:157283. 10.1016/j.phymed.2025.157283. 41086675

[57] Niu Y, Xiang M, Yang W, Fang Y, Qian H, Sun Y. NAD+/SIRT1 pathway regulates glycolysis to promote oxaliplatin resistance in colorectal cancer. World J Gastroenterol. 2025;31:100785. 10.3748/wjg.v31.i11.100785. PMC11924001 40124268

[58] Zhang M, Zhang Z, Lou Q, Zhang X, Yin F, Yin Y, et al. SIRT1/P53 pathway is involved in the arsenic induced aerobic glycolysis in hepatocytes L-02 cells. Environ Sci Pollut Res Int. 2023;30:73799–811. 10.1007/s11356-023-27570-5. 37195614

[59] You Q, Wang J, Yu Y, Li F, Meng L, Chen M, et al. The histone deacetylase SIRT6 promotes glycolysis through the HIF-1α/HK2 signaling axis and induces erlotinib resistance in non-small cell lung cancer. Apoptosis. 2022;27:883–98. 10.1007/s10495-022-01751-y. PMC9617843 35915188

[60] Gupta P, Sheikh T, Sen E. SIRT6 regulated nucleosomal occupancy affects Hexokinase 2 expression. Exp Cell Res. 2017;357:98–106. 10.1016/j.yexcr.2017.05.005. 28478957

[61] Wang X, Zhao X, Zheng X, Peng X, Chen J, Wang Y, et al. Sirt6 loss activates Got1 and facilitates cleft palate through abnormal activating glycolysis. Cell Death Dis. 2025;16:159. 10.1038/s41419-025-07465-8. PMC11885815 40050262

[62] Kwon OK, Bang IH, Choi SY, Jeon JM, Na A, Gao Y, et al. LDHA Desuccinylase Sirtuin 5 as A Novel Cancer Metastatic Stimulator in Aggressive Prostate Cancer. Genomics Proteomics Bioinformatics. 2023;21:177–89. 10.1016/j.gpb.2022.02.004. PMC10372916 35278714

[63] Yang H, Zhu R, Zhao X, Liu L, Zhou Z, Zhao L, et al. Sirtuin-mediated deacetylation of hnRNP A1 suppresses glycolysis and growth in hepatocellular carcinoma. Oncogene. 2019;38:4915–31. 10.1038/s41388-019-0764-z. 30858544

[64] Lian B, Zhang J, Yin X, Wang J, Li L, Ju Q, et al. SIRT1 improves lactate homeostasis in the brain to alleviate parkinsonism via deacetylation and inhibition of PKM2. Cell Rep Med. 2024;5:101684. 10.1016/j.xcrm.2024.101684. PMC11384727 39128469

[65] Guo J, Nie J, Li D, Zhang H, Zhao T, Zhang S, et al. The role of NAD-dependent deacetylase sirtuin-2 in liver metabolic stress through regulating pyruvate kinase M2 ubiquitination. J Transl Med. 2024;22:656. 10.1186/s12967-024-05435-w. PMC11247741 39004743

[66] Hao L, Park J, Jang H, Bae EJ, Park B. Inhibiting Protein Kinase Activity of Pyruvate Kinase M2 by SIRT2 Deacetylase Attenuates Psoriasis. J Invest Dermatol. 2021;141:355-363.e6. 10.1016/j.jid.2020.06.024. 32679047

[67] Zadoorian A, Du X, Yang H. Lipid droplet biogenesis and functions in health and disease. Nat Rev Endocrinol. 2023;19:443–59. 10.1038/s41574-023-00845-0. PMC10204695 37221402

[68] Wei W, Qin B, Wen W, Zhang B, Luo H, Wang Y, et al. FBXW7β loss-of-function enhances FASN-mediated lipogenesis and promotes colorectal cancer growth. Signal Transduct Target Ther. 2023;8:187. 10.1038/s41392-023-01405-8. PMC10195794 37202390

[69] Zaganjor E, Yoon H, Spinelli JB, Nunn ER, Laurent G, Keskinidis P, et al. SIRT4 is an early regulator of branched-chain amino acid catabolism that promotes adipogenesis. Cell Rep. 2021;36:109345. 10.1016/j.celrep.2021.109345. PMC8320302 34260923

[70] Masri S, Rigor P, Cervantes M, Ceglia N, Sebastian C, Xiao C, et al. Partitioning circadian transcription by SIRT6 leads to segregated control of cellular metabolism. Cell. 2014;158:659–72. 10.1016/j.cell.2014.06.050. PMC5472354 25083875

[71] Elhanati S, Kanfi Y, Varvak A, Roichman A, Carmel-Gross I, Barth S, et al. Multiple regulatory layers of SREBP1/2 by SIRT6. Cell Rep. 2013;4:905–12. 10.1016/j.celrep.2013.08.006. 24012758

[72] Zhu C, Huang M, Kim HG, Chowdhury K, Gao J, Liu S, et al. SIRT6 controls hepatic lipogenesis by suppressing LXR, ChREBP, and SREBP1. Biochim Biophys Acta Mol Basis Dis. 2021;1867:166249. 10.1016/j.bbadis.2021.166249. PMC8488016 34425214

[73] Bian C, Zhang H, Gao J, Wang Y, Li J, Guo D, et al. SIRT6 regulates SREBP1c-induced glucolipid metabolism in liver and pancreas via the AMPKα-mTORC1 pathway. Lab Invest. 2022;102:474–84. 10.1038/s41374-021-00715-1. 34923569

[74] Ka SO, Bang IH, Bae EJ, Park BH. Hepatocyte-specific sirtuin 6 deletion predisposes to nonalcoholic steatohepatitis by up-regulation of Bach1, an Nrf2 repressor. FASEB J. 2017;31:3999–4010. 10.1096/fj.201700098RR. 28536120

[75] Cai X, Liu Z, Dong X, Wang Y, Zhu L, Li M, et al. Hypoglycemic and lipid lowering effects of theaflavins in high-fat diet-induced obese mice. Food Funct. 2021;12:9922–31. 10.1039/d1fo01966j. 34492673

[76] Dong Z, Li C, Yin C, Xu M, Liu S, Gao M. LncRNA PU.1 AS regulates arsenic-induced lipid metabolism through EZH2/Sirt6/SREBP-1c pathway. J Environ Sci (China). 2019;85:138–46. 10.1016/j.jes.2019.05.019. 31471020

[77] Bradshaw PC. Acetyl-CoA Metabolism and Histone Acetylation in the Regulation of Aging and Lifespan. Antioxidants (Basel). 2021;10:572. 10.3390/antiox10040572. PMC8068152 33917812

[78] Zheng W, Tasselli L, Li TM, Chua KF. Mammalian SIRT6 Represses Invasive Cancer Cell Phenotypes through ATP Citrate Lyase (ACLY)-Dependent Histone Acetylation. Genes (Basel). 2021;12:1460. 10.3390/genes12091460. PMC8466468 34573442

[79] Wu G, Cheng H, Guo H, Li Z, Li D, Xie Z. Tea polyphenol EGCG ameliorates obesity-related complications by regulating lipidomic pathway in leptin receptor knockout rats. J Nutr Biochem. 2023;118:109349. 10.1016/j.jnutbio.2023.109349. 37085056

[80] Wu G, Sun X, Cheng H, Xu S, Li D, Xie Z. Large Yellow Tea Extract Ameliorates Metabolic Syndrome by Suppressing Lipogenesis through SIRT6/SREBP1 Pathway and Modulating Microbiota in Leptin Receptor Knockout Rats. Foods. 2022;11:1638. 10.3390/foods11111638. PMC9180543 35681388

[81] Tao R, Xiong X, DePinho RA, Deng CX, Dong XC. Hepatic SREBP-2 and cholesterol biosynthesis are regulated by FoxO3 and Sirt6. J Lipid Res. 2013;54:2745–53. 10.1194/jlr.M039339. PMC3770087 23881913

[82] Stöhr R, Mavilio M, Marino A, Casagrande V, Kappel B, Möllmann J, et al. ITCH modulates SIRT6 and SREBP2 to influence lipid metabolism and atherosclerosis in ApoE null mice. Sci Rep. 2015;5:9023. 10.1038/srep09023. PMC4361881 25777360

[83] Hu J, Lei H, Chen J, Liu L, Gui Y, Sun K, et al. Aerobic exercise alleviates statin-induced PCSK9 upregulation by increasing epoxyeicosatrienoic acid levels through the FoxO3a-Sirt6 axis. J Sport Health Sci. 2025;14:101007. 10.1016/j.jshs.2024.101007. PMC11863326 39510317

[84] Wei Z, Xia J, Li J, Cai J, Shan J, Zhang C, et al. SIRT1 promotes glucolipid metabolic conversion to facilitate tumor development in colorectal carcinoma. Int J Biol Sci. 2023;19:1925–40. 10.7150/ijbs.76704. PMC10092765 37063423

[85] Zhang Y, Li T, Pan M, Wang W, Huang W, Yuan Y, et al. SIRT1 prevents cigarette smoking-induced lung fibroblasts activation by regulating mitochondrial oxidative stress and lipid metabolism. J Transl Med. 2022;20:222. 10.1186/s12967-022-03408-5. PMC9107262 35568871

[86] Li X, Song D, Chen Y, Huang C, Liu A, Wu Q, et al. NSD2 methylates AROS to promote SIRT1 activation and regulates fatty acid metabolism-mediated cancer radiotherapy. Cell Rep. 2023;42:113126. 10.1016/j.celrep.2023.113126. 37756162

[87] Naiman S, Huynh FK, Gil R, Glick Y, Shahar Y, Touitou N, et al. SIRT6 Promotes Hepatic Beta-Oxidation via Activation of PPARα. Cell Rep. 2019;29:4127-4143.e8. 10.1016/j.celrep.2019.11.067. PMC7165364 31851938

[88] Yang Z, Yu Y, Sun N, Zhou L, Zhang D, Chen H, et al. Ginsenosides Rc, as a novel SIRT6 activator, protects mice against high fat diet induced NAFLD. J Ginseng Res. 2023;47:376–84. 10.1016/j.jgr.2020.07.005. PMC10214131 37252281

[89] Cui T, Xiao X, Pan Z, Tang K, Zhong Y, Chen Y, et al. Harnessing the Therapeutic Potential of Ginsenoside Rd for Activating SIRT6 in Treating a Mouse Model of Nonalcoholic Fatty Liver Disease. ACS Omega. 2023;8:29735–45. 10.1021/acsomega.3c04122. PMC10433470 37599957

[90] Fan S, Chen W, Li Y, Guo K, Tang H, Ye J, et al. Qige Decoction attenuated non-alcoholic fatty liver disease through regulating SIRT6-PPARα-mediated fatty acid oxidation. Phytomedicine. 2025;138:156395. 10.1016/j.phymed.2025.156395. 39855055

[91] Chen HX, Yang F, He XQ, Li T, Sun YZ, Song JP, et al. Garcinia Biflavonoid 1 Improves Lipid Metabolism in HepG2 Cells via Regulating PPARα. Molecules. 2022;27:1978. 10.3390/molecules27061978. PMC8950208 35335339

[92] Kim Y, Lim H, Cho YE, Hwang S. The SIRT6 Activator MDL-800 Inhibits PPARα and Fatty acid Oxidation-Related Gene Expression in Hepatocytes. Biomol Ther (Seoul). 2025;33:438–46. 10.4062/biomolther.2024.251. PMC12059364 40199556

[93] Guo Y, Zhang Z, Wen Z, Kang X, Wang D, Zhang L, et al. Mitochondrial SIRT2-mediated CPT2 deacetylation prevents diabetic cardiomyopathy by impeding cardiac fatty acid oxidation. Int J Biol Sci. 2025;21:725–44. 10.7150/ijbs.102834. PMC11705638 39781464

[94] Wang R, Xu H, Tan B, Yi Q, Sun Y, Xiang H, et al. SIRT3 promotes metabolic maturation of human iPSC-derived cardiomyocytes via OPA1-controlled mitochondrial dynamics. Free Radic Biol Med. 2023;195:270–82. 10.1016/j.freeradbiomed.2022.12.101. 36596388

[95] Chen J, Zhou F, Zhang L, Lou R, Zhang C, Wan J, et al. Adipocyte-expressed SIRT3 manipulates carnitine pool to orchestrate metabolic reprogramming and polarization of macrophages. Cell Death Dis. 2025;16:381. 10.1038/s41419-025-07699-6. PMC12078679 40368890

[96] Wu M, Tan J, Cao Z, Cai Y, Huang Z, Chen Z, et al. Sirt5 improves cardiomyocytes fatty acid metabolism and ameliorates cardiac lipotoxicity in diabetic cardiomyopathy via CPT2 de-succinylation. Redox Biol. 2024;73:103184. 10.1016/j.redox.2024.103184. PMC11091707 38718533

[97] Wu J, Wang Y, Zhang Q, Lan Y, Chen M, Wang J, et al. SIRT5 regulates fatty acid oxidation and mitochondrial oxidative stress to exacerbate airway inflammation in experimental asthma. Cell Signal. 2025;136:112149. 10.1016/j.cellsig.2025.112149. 40975508

[98] Kim HG, Huang M, Xin Y, Zhang Y, Zhang X, Wang G, et al. The epigenetic regulator SIRT6 protects the liver from alcohol-induced tissue injury by reducing oxidative stress in mice. J Hepatol. 2019;71:960–9. 10.1016/j.jhep.2019.06.019. PMC6801027 31295533

[99] Li L, Zeng J, Zhang X, Feng Y, Lei JH, Xu X, et al. Sirt6 ablation in the liver causes fatty liver that increases cancer risky by upregulating Serpina12. EMBO Rep. 2024;25:1361–86. 10.1038/s44319-024-00071-3. PMC10933290 38332150

[100] Zou D, Liu L, Zeng Y, Wang H, Dai D, Xu M. LncRNA MEG3 up-regulates SIRT6 by ubiquitinating EZH2 and alleviates nonalcoholic fatty liver disease. Cell Death Discov. 2022;8:103. 10.1038/s41420-022-00889-7. PMC8901640 35256601

[101] Pan Z, Guo J, Tang K, Chen Y, Gong X, Chen Y, et al. Ginsenoside Rc Modulates SIRT6-NRF2 Interaction to Alleviate Alcoholic Liver Disease. J Agric Food Chem. 2022;70:14220–34. 10.1021/acs.jafc.2c06146. 36300841

[102] Jiang Y, Zhang J, Liu W, Qian X, Zhuang X, Hu C. Scutellariae Radix and Coptidis Rhizoma improve NAFLD via regulation of SIRT6/ACSL5 pathway and SCD1. J Ethnopharmacol. 2025;348:119834. 10.1016/j.jep.2025.119834. 40254111

[103] Li S, Yuan Z. More than just a histone deacetylase: cytoplasmic SIRT6 facilitates fatty acid oxidation through ACSL5 deacetylation. Acta Biochim Biophys Sin (Shanghai). 2023;55:525–7. 10.3724/abbs.2023030. PMC10160229 36880305

[104] Bang IH, Kwon OK, Hao L, Park D, Chung MJ, Oh BC, et al. Deacetylation of XBP1s by sirtuin 6 confers resistance to ER stress-induced hepatic steatosis. Exp Mol Med. 2019;51:1–11. 10.1038/s12276-019-0309-0. PMC6802632 31541078

[105] Yang Z, Li S, Zhao C, Zhao Z, Tan J, Zhang L, et al. X-Box binding protein 1 downregulates SIRT6 to promote injury in pancreatic ductal epithelial cells. Immun Inflamm Dis. 2024;12:e1301. 10.1002/iid3.1301. PMC11225082 38967361

[106] Du L, Guo C, Zeng S, Yu K, Liu M, Li Y. Sirt6 overexpression relieves ferroptosis and delays the progression of diabetic nephropathy via Nrf2/GPX4 pathway. Ren Fail. 2024;46:2377785. 10.1080/0886022X.2024.2377785. PMC11293269 39082470

[107] Hong J, Mei C, Abbas Raza SH, Khan R, Cheng G, Zan L. SIRT6 cooperates with SIRT5 to regulate bovine preadipocyte differentiation and lipid metabolism via the AMPKα signaling pathway. Arch Biochem Biophys. 2020;681:108260. 10.1016/j.abb.2020.108260. 31926163

[108] Chen Z, Liang W, Hu J, Zhu Z, Feng J, Ma Y, et al. Sirt6 deficiency contributes to mitochondrial fission and oxidative damage in podocytes via ROCK1-Drp1 signalling pathway. Cell Prolif. 2022;55:e13296. 10.1111/cpr.13296. PMC9528772 35842903

[109] Zhao Y, Jia X, Yang X, Bai X, Lu Y, Zhu L, et al. Deacetylation of Caveolin-1 by Sirt6 induces autophagy and retards high glucose-stimulated LDL transcytosis and atherosclerosis formation. Metabolism. 2022;131:155162. 10.1016/j.metabol.2022.155162. 35167876

[110] He J, Zhang G, Pang Q, Yu C, Xiong J, Zhu J, et al. SIRT6 reduces macrophage foam cell formation by inducing autophagy and cholesterol efflux under ox-LDL condition. FEBS J. 2017;284:1324–37. 10.1111/febs.14055. 28296196

[111] Wang W, Liang J, Zhang Y, Wang J, Miao X, Chang Y, et al. Myeloid sirtuin 6 deficiency causes obesity in mice by inducing norepinephrine degradation to limit thermogenic tissue function. Sci Signal. 2025;18:eadl6441. 10.1126/scisignal.adl6441. 40067908

[112] Meng Y, Guo D, Lin L, Zhao H, Xu W, Luo S, et al. Glycolytic enzyme PFKL governs lipolysis by promoting lipid droplet-mitochondria tethering to enhance β-oxidation and tumor cell proliferation. Nat Metab. 2024;6:1092–107. 10.1038/s42255-024-01047-2. 38773347

[113] Qiu B, Li S, Li M, Wang S, Mu G, Chen K, et al. KAT8 acetylation-controlled lipolysis affects the invasive and migratory potential of colorectal cancer cells. Cell Death Dis. 2023;14:164. 10.1038/s41419-023-05582-w. PMC9970984 36849520

[114] Jung SM, Hung CM, Hildebrand SR, Sanchez-Gurmaches J, Martinez-Pastor B, Gengatharan JM, et al. Non-canonical mTORC2 Signaling Regulates Brown Adipocyte Lipid Catabolism through SIRT6-FoxO1. Mol Cell. 2019;75:807-822.e8. 10.1016/j.molcel.2019.07.023. PMC7388077 31442424

[115] Kuang J, Zhang Y, Liu Q, Shen J, Pu S, Cheng S, et al. Fat-Specific Sirt6 Ablation Sensitizes Mice to High-Fat Diet-Induced Obesity and Insulin Resistance by Inhibiting Lipolysis. Diabetes. 2017;66:1159–71. 10.2337/db16-1225. 28250020

[116] Wu D, Bang IH, Park BH, Bae EJ. Loss of Sirt6 in adipocytes impairs the ability of adipose tissue to adapt to intermittent fasting. Exp Mol Med. 2021;53:1298–306. 10.1038/s12276-021-00664-1. PMC8492715 34493807

[117] Wang Y, Liu S, Ni M, Chen Y, Chen R, Wang J, et al. Terf2ip deficiency accelerates non-alcoholic steatohepatitis through regulating lipophagy and fatty acid oxidation via Sirt1/AMPK pathway. Free Radic Biol Med. 2024;220:78–91. 10.1016/j.freeradbiomed.2024.04.238. 38697492

[118] Gao S, Yang Q, Liu Z, Kong W, Chen J, Li X, et al. Metformin alleviates HFD-induced oxidative stress in hepatocyte via activating SIRT6/PGC-1α/ENDOG signaling. Clin Sci (Lond). 2022;136:1711–30. 10.1042/CS20220242. 36315407

[119] Yang Z, Huang R, Wang Y, Guan Q, Li D, Wu Y, et al. SIRT6 drives sensitivity to ferroptosis in anaplastic thyroid cancer through NCOA4-dependent autophagy. Am J Cancer Res. 2023;13:464–74. PMC9989618 36895980

[120] Gao S, Yang Q, Peng Y, Kong W, Liu Z, Li Z, et al. SIRT6 regulates obesity-induced oxidative stress via ENDOG/SOD2 signaling in the heart. Cell Biol Toxicol. 2023;39:1489–507. 10.1007/s10565-022-09735-z. 35798905

[121] Wang M, Wu Y, He Y, Liu J, Chen Y, Huang J, et al. SIRT1 upregulation promotes epithelial-mesenchymal transition by inducing senescence escape in endometriosis. Sci Rep. 2022;12:12302. 10.1038/s41598-022-16629-x. PMC9296487 35853978

[122] Hou D, Yu T, Lu X, Hong JY, Yang M, Zi Y, et al. Sirt2 inhibition improves gut epithelial barrier integrity and protects mice from colitis. Proc Natl Acad Sci U S A. 2024;121:e2319833121. 10.1073/pnas.2319833121. PMC11066986 38648480

[123] Ma C, Yang C, Peng A, Sun T, Ji X, Mi J, et al. Pan-cancer spatially resolved single-cell analysis reveals the crosstalk between cancer-associated fibroblasts and tumor microenvironment. Mol Cancer. 2023;22:170. 10.1186/s12943-023-01876-x. PMC10571470 37833788

[124] Niu N, Shen X, Wang Z, Chen Y, Weng Y, Yu F, et al. Tumor cell-intrinsic epigenetic dysregulation shapes cancer-associated fibroblasts heterogeneity to metabolically support pancreatic cancer. Cancer Cell. 2024;42:869-884.e9. 10.1016/j.ccell.2024.03.005. 38579725

[125] Yu X, Jin Z, Raza F, Zhang P, Wu J, Ren M, et al. SIRT6 Reduces Rheumatoid Arthritis Injury by Inhibiting MyD88-ERK Signaling Pathway. Front Biosci (Landmark Ed). 2024;29:5. 10.31083/j.fbl2901005. 38287796

[126] Wang T, Qin Y, Qiao J, Liu Y, Wang L, Zhang X. Overexpression of SIRT6 regulates NRF2/HO-1 and NF-κB signaling pathways to alleviate UVA-induced photoaging in skin fibroblasts. J Photochem Photobiol B. 2023;249:112801. 10.1016/j.jphotobiol.2023.112801. 37897855

[127] Zhou T, Chen Y, Wang C, Huang Z, Tan Z, Ma Y. SIRT6 inhibits the proliferation and collagen synthesis of keloid fibroblasts through MAPK/ERK pathway. Discov Med. 2024;36:1430–40. 10.24976/Discov.Med.202436186.133. 39054714

[128] Zhong X, Huang M, Kim HG, Zhang Y, Chowdhury K, Cai W, et al. SIRT6 Protects Against Liver Fibrosis by Deacetylation and Suppression of SMAD3 in Hepatic Stellate Cells. Cell Mol Gastroenterol Hepatol. 2020;10:341–64. 10.1016/j.jcmgh.2020.04.005. PMC7327931 32305562

[129] Yu S, Yu H, Geng S, Tang R, Zhang A, Zhang Y, et al. Deacetylase SIRT1 modulates antiviral innate immunity and autoimmune diseases. Int J Biol Macromol. 2025;329:147873. 10.1016/j.ijbiomac.2025.147873. 41005403

[130] Labiner HE, Sas KM, Hoying J, Sepeda JA, Wolf N, Perez EC, et al. SIRT1 downregulation in pneumonia is associated with an immature neutrophil response and increased disease severity. J Trauma Acute Care Surg. 2024;96:557–65. 10.1097/TA.0000000000004212. 37962211

[131] Zhang M, Qi L, Li J, Yuan N, Zhai Y, Hao M, et al. SIRT2 inhibition enhances mitochondrial apoptosis in Brucella-infected bovine placental trophoblast cells. Vet Res. 2025;56:97. 10.1186/s13567-025-01518-8. PMC12049057 40317067

[132] Gandhirajan A, Roychowdhury S, Kibler C, Cross E, Abraham S, Bellar A, et al. SIRT2-PFKP interaction dysregulates phagocytosis in macrophages with acute ethanol-exposure. Front Immunol. 2022;13:1079962. 10.3389/fimmu.2022.1079962. PMC9972587 36865524

[133] Li Y, Bie J, Song C, Li Y, Zhang T, Li H, et al. SIRT2 negatively regulates the cGAS-STING pathway by deacetylating G3BP1. EMBO Rep. 2023;24:e57500. 10.15252/embr.202357500. PMC10702829 37870259

[134] Gao J, Huang C, Kong L, Zhou W, Sun M, Wei T, et al. SIRT3 Regulates Clearance of Apoptotic Cardiomyocytes by Deacetylating Frataxin. Circ Res. 2023;133:631–47. 10.1161/CIRCRESAHA.123.323160. PMC10498872 37646156

[135] Guan C, Huang X, Yue J, Xiang H, Shaheen S, Jiang Z, et al. SIRT3-mediated deacetylation of NLRC4 promotes inflammasome activation. Theranostics. 2021;11:3981–95. 10.7150/thno.55573. PMC7914345 33664876

[136] Xie Y, He Y, Liang J, Liu J, Ke C, Mo X, et al. SIRT5 Alleviated Eosinophilic Asthma Through ROS Inhibition and Nrf2/HO-1 Activation. Inflammation. 2025;48:3169–79. 10.1007/s10753-025-02257-w. PMC12596428 39946006

[137] Pan Y, Zhang Q, Li C, Li X, Li S, Wang Y, et al. SIRT5 Alleviates Apoptosis of Vascular Endothelial Cells Under Simulated Microgravity via Desuccinylation of ERO1A. Int J Mol Sci. 2025;26:2908. 10.3390/ijms26072908. PMC11988372 40243486

[138] Wang J, Luo J, Rotili D, Mai A, Steegborn C, Xu S, et al. SIRT6 Protects Against Lipopolysaccharide-Induced Inflammation in Human Pulmonary Lung Microvascular Endothelial Cells. Inflammation. 2024;47:323–32. 10.1007/s10753-023-01911-5. 37819455

[139] Wei L, Kang M, Zhang G, Meng Y, Qin H. SIRT6 Overexpression Enhances Diabetic Foot Ulcer Healing via Nrf2 Pathway Activation. Inflammation. 2025;48:3858–77. 10.1007/s10753-025-02297-2. PMC12722270 40199836

[140] Li P, Jin Y, Qi F, Wu F, Luo S, Cheng Y, et al. SIRT6 Acts as a Negative Regulator in Dengue Virus-Induced Inflammatory Response by Targeting the DNA Binding Domain of NF-κB p65. Front Cell Infect Microbiol. 2018;8:113. 10.3389/fcimb.2018.00113. PMC5900784 29686974

[141] Liu F, Bu HF, Geng H, De Plaen IG, Gao C, Wang P, et al. Sirtuin-6 preserves R-spondin-1 expression and increases resistance of intestinal epithelium to injury in mice. Mol Med. 2017;23:272–84. 10.2119/molmed.2017.00085. PMC5654826 29387864

[142] Arsiwala T, Pahla J, van Tits LJ, Bisceglie L, Gaul DS, Costantino S, et al. Sirt6 deletion in bone marrow-derived cells increases atherosclerosis - Central role of macrophage scavenger receptor 1. J Mol Cell Cardiol. 2020;139:24–32. 10.1016/j.yjmcc.2020.01.002. 31972266

[143] Bresque M, Cal K, Pérez-Torrado V, Colman L, Rodríguez-Duarte J, Vilaseca C, et al. SIRT6 stabilization and cytoplasmic localization in macrophages regulates acute and chronic inflammation in mice. J Biol Chem. 2022;298:101711. 10.1016/j.jbc.2022.101711. PMC8913316 35150745

[144] Jin J, Li W, Wang T, Park BH, Park SK, Kang KP. Loss of Proximal Tubular Sirtuin 6 Aggravates Unilateral Ureteral Obstruction-Induced Tubulointerstitial Inflammation and Fibrosis by Regulation of β-Catenin Acetylation. Cells. 2022;11:1477. 10.3390/cells11091477. PMC9100256 35563783

[145] Song MY, Kim SH, Ryoo GH, Kim MK, Cha HN, Park SY, et al. Adipose sirtuin 6 drives macrophage polarization toward M2 through IL-4 production and maintains systemic insulin sensitivity in mice and humans. Exp Mol Med. 2019;51:1–10. 10.1038/s12276-019-0256-9. PMC6529411 31113929

[146] Song MY, Jeon YG, Do Yang J, Moon YJ, Kim JB, Bae EJ, et al. Sirt6 deficiency in mast cells promotes adipose fibroinflammation in obesity through galectin-3 signaling. Nat Commun. 2026;17:57. 10.1038/s41467-025-66040-z. PMC12775440 41495031

[147] Gao X, Liu X, Han Z, Liao H, Li R. Friend or foe? The role of SIRT6 on macrophage polarized to M2 subtype in acute kidney injury to chronic kidney disease. Ren Fail. 2025;47:2482121. 10.1080/0886022X.2025.2482121. PMC12016254 40260529

[148] Hao L, Nam KH, Lee GJ, Kim D, Shin JM, Lee Y, et al. SIRT1 downregulation provokes immune-inflammatory responses in hair follicle outer root sheath cells and may contribute to development of alopecia areata. J Dermatol Sci. 2023;111:2–9. 10.1016/j.jdermsci.2023.05.005. 37268558

[149] Hisada R, Yoshida N, Umeda M, Burbano C, Bhargava R, Scherlinger M, et al. The deacetylase SIRT2 contributes to autoimmune disease pathogenesis by modulating IL-17A and IL-2 transcription. Cell Mol Immunol. 2022;19:738–50. 10.1038/s41423-022-00874-1. PMC9151795 35523941

[150] Wang TT, Han T, Xiao X, Guo D, Sun X, Liu Y, et al. SIRT3 deficiency reduces PFKFB3-driven T-cell glycolysis and promotes arthritic inflammation. Sci China Life Sci. 2025;68:1755–69. 10.1007/s11427-024-2823-2. 40029452

[151] Zhou Q, Wang Y, Lu Z, Wang B, Li L, You M, et al. Mitochondrial dysfunction caused by SIRT3 inhibition drives proinflammatory macrophage polarization in obesity. Obesity (Silver Spring). 2023;31:1050–63. 10.1002/oby.23707. 36894333

[152] Cha X, Zou Q, Li F, Wang T, Wang S, Cai B, et al. SIRT5 exacerbates eosinophilic chronic rhinosinusitis by promoting polarization of M2 macrophage. J Allergy Clin Immunol. 2024;154:644–56. 10.1016/j.jaci.2024.04.028. 38761998

[153] Gamez-Garcia A, Espinosa-Alcantud M, Bueno-Costa A, Alari-Pahissa E, Marazuela-Duque A, Thackray JK, et al. A SIRT7-dependent acetylation switch regulates early B cell differentiation and lineage commitment through Pax5. Nat Immunol. 2024;25:2308–19. 10.1038/s41590-024-01995-7. PMC11588656 39424985

[154] Ge J, Zhang X, Tang F, Liu Y. SIRT7 ameliorates Th17/Treg imbalance by desuccinylation of STAT3 to improve immune thrombocytopenia. Clin Transl Immunology. 2025;14:e70048. 10.1002/cti2.70048. PMC12263510 40672112

[155] Zhang Q, Liang Z, Zhang J, Lei T, Dong X, Su H, et al. Sirt6 Regulates the Development of Medullary Thymic Epithelial Cells and Contributes to the Establishment of Central Immune Tolerance. Front Cell Dev Biol. 2021;9:655552. 10.3389/fcell.2021.655552. PMC8044826 33869219

[156] Wang Z, Guo W, Zhang X, Wei Y, Zhang W, Du N, et al. Tumor microenvironment-associated oxidative stress impairs SIRT1 secretion to suppress anti-tumor immune response. Cell Rep. 2025;44:115679. 10.1016/j.celrep.2025.115679. 40343797

[157] Zi R, Zhao X, Liu L, Wang Y, Zhang R, Bian Z, et al. Metabolic-Immune Suppression Mediated by the SIRT1-CX3CL1 Axis Induces Functional Enhancement of Regulatory T Cells in Colorectal Carcinoma. Adv Sci (Weinh). 2025;12:e2404734. 10.1002/advs.202404734. PMC12061293 39783838

[158] Hamaidi I, Zhang L, Kim N, Wang M, Iclozan C, Fang B, et al. Sirt2 Inhibition Enhances Metabolic Fitness and Effector Functions of Tumor-Reactive T Cells. Cell Metab. 2020;32:420-436.e12. 10.1016/j.cmet.2020.07.008. PMC7484212 32768387

[159] Gao Q, Yang L, Ye S, Mai M, Liu Y, Jiang X, et al. Targeting SIRT2 induces MLH1 deficiency and boosts antitumor immunity in preclinical colorectal cancer models. Sci Transl Med. 2025;17:eadv0766. 10.1126/scitranslmed.adv0766. 40668890

[160] Cai N, Cheng K, Ma Y, Liu S, Tao R, Li Y, et al. Targeting MMP9 in CTNNB1 mutant hepatocellular carcinoma restores CD8+ T cell-mediated antitumour immunity and improves anti-PD-1 efficacy. Gut. 2024;73:985–99. 10.1136/gutjnl-2023-331342. PMC11103337 38123979

[161] Chen M, Xu M, Zhu C, Wang H, Zhao Q, Zhou F. Sirtuin2 enhances the tumoricidal function of liver natural killer cells in a mouse hepatocellular carcinoma model. Cancer Immunol Immunother. 2019;68:961–71. 10.1007/s00262-019-02337-5. PMC11028107 30955067

[162] Zhang M, Acklin S, Gillenwater J, Du W, Patra M, Yu H, et al. SIRT2 promotes murine melanoma progression through natural killer cell inhibition. Sci Rep. 2021;11:12988. 10.1038/s41598-021-92445-z. PMC8217567 34155309

[163] Wang T, Cao Y, Zheng Q, Tu J, Zhou W, He J, et al. SENP1-Sirt3 Signaling Controls Mitochondrial Protein Acetylation and Metabolism. Mol Cell. 2019;75:823-834.e5. 10.1016/j.molcel.2019.06.008. 31302001

[164] Lv J, Zhang Y, Wu Q, Jiang P, Lin Y. Inhibition of SIRT4 promotes bladder cancer progression and immune escape via attenuating CD8+ T cells function. Int Immunopharmacol. 2025;147:113906. 10.1016/j.intimp.2024.113906. 39756164

[165] Sun R, Zhang Z, Bao R, Guo X, Gu Y, Yang W, et al. Loss of SIRT5 promotes bile acid-induced immunosuppressive microenvironment and hepatocarcinogenesis. J Hepatol. 2022;77:453–66. 10.1016/j.jhep.2022.02.030. 35292350

[166] Fan G, Yu B, Tang L, Zhu R, Chen J, Zhu Y, et al. TSPAN8(+) myofibroblastic cancer-associated fibroblasts promote chemoresistance in patients with breast cancer. Sci Transl Med. 2024;16:eadj5705. 10.1126/scitranslmed.adj5705. 38569015

[167] Okabe K, Yaku K, Tobe K, Nakagawa T. Implications of altered NAD metabolism in metabolic disorders. J Biomed Sci. 2019;26:34. 10.1186/s12929-019-0527-8. PMC6511662 31078136

[168] Yoshino J, Baur JA, Imai S. NAD+ Intermediates: The Biology and Therapeutic Potential of NMN and NR. Cell Metab. 2018;27:513–28. 10.1016/j.cmet.2017.11.002. PMC5842119 29249689

[169] Kim H, Xiao C, Wang R, Lahusen T, Xu X, Vassilopoulos A, et al. Hepatic-specific disruption of SIRT6 in mice results in fatty liver formation due to enhanced glycolysis and triglyceride synthesis. Cell Metab. 2010;12:224–36. 10.1016/j.cmet.2010.06.009. PMC2935915 20816089

[170] Wojciak G, Szymura J, Szygula Z, Gradek J, Wiecek M. The Effect of Repeated Whole-Body Cryotherapy on Sirt1 and Sirt3 Concentrations and Oxidative Status in Older and Young Men Performing Different Levels of Physical Activity. Antioxidants (Basel). 2020;10:37. 10.3390/antiox10010037. PMC7823702 33396247

[171] Lee AY, Christensen SM, Duong N, Tran Q, Xiong HM, Huang J, et al. Sirt3 Pharmacologically Promotes Insulin Sensitivity through PI3/AKT/mTOR and Their Downstream Pathway in Adipocytes. Int J Mol Sci. 2022;23:3740. 10.3390/ijms23073740. PMC8998733 35409099

[172] Xian Y, Liu B, Shen T, Yang L, Peng R, Shen H, et al. Enhanced SIRT3 expression restores mitochondrial quality control mechanism to reverse osteogenic impairment in type 2 diabetes mellitus. Bone Res. 2025;13:30. 10.1038/s41413-024-00399-5. PMC11873136 40025004

[173] Tang H, Zhou Y, Ma L, Ye Y, Xiao Q, Tang J, et al. SIRT3 alleviates mitochondrial dysfunction and senescence in diabetes-associated periodontitis by deacetylating LRPPRC. Free Radic Biol Med. 2025;227:407–19. 10.1016/j.freeradbiomed.2024.11.033. 39557134

[174] Gao R, Chen Z, Wu Y, Chen R, Zheng W, Qi L, et al. SIRT3 alleviates mitochondrial dysfunction induced by recurrent low glucose and improves the supportive function of astrocytes to neurons. Free Radic Biol Med. 2022;193:405–20. 10.1016/j.freeradbiomed.2022.10.313. 36306990

[175] Zhang Y, Liu Y, Hou M, Xia X, Liu J, Xu Y, et al. Reprogramming of Mitochondrial Respiratory Chain Complex by Targeting SIRT3-COX4I2 Axis Attenuates Osteoarthritis Progression. Adv Sci (Weinh). 2023;10:e2206144. 10.1002/advs.202206144. PMC10074136 36683245

[176] Zhang Y, Wen P, Luo J, Ding H, Cao H, He W, et al. Sirtuin 3 regulates mitochondrial protein acetylation and metabolism in tubular epithelial cells during renal fibrosis. Cell Death Dis. 2021;12:847. 10.1038/s41419-021-04134-4. PMC8437958 34518519

[177] Wortham M, Ramms B, Zeng C, Benthuysen JR, Sai S, Pollow DP, et al. The protein deacetylase SIRT2 exerts metabolic control over adaptive β cell proliferation. J Clin Invest. 2025;135:e187020. 10.1172/JCI187020. PMC12483614 40762838

[178] Fasina OB, Li L, Chen D, Yi M, Xiang L, Qi J. Tetradecyl 2,3-dihydroxybenzoate improves cognitive function in AD mice by modulating autophagy and inflammation through IPA and Hsc70 targeting. Int J Mol Sci. 2024;25:11719. 10.3390/ijms252111719. PMC11547019 39519271

[179] Zhao N, Xia J, Xu B. Physical exercise may exert its therapeutic influence on Alzheimer’s disease through the reversal of mitochondrial dysfunction via SIRT1-FOXO1/3-PINK1-Parkin-mediated mitophagy. J Sport Health Sci. 2021;10:1–3. 10.1016/j.jshs.2020.08.009. PMC7856556 32861777

[180] Tian J, Huang T, Chen J, Wang J, Chang S, Xu H, et al. SIRT1 slows the progression of lupus nephritis by regulating the NLRP3 inflammasome through ROS/TRPM2/Ca2+ channel. Clin Exp Med. 2023;23:3465–78. 10.1007/s10238-023-01093-2. 37261640

[181] Zhang Y, Zhang N, Zou Y, Song C, Cao K, Wu B, et al. Deacetylation of Septin4 by SIRT2 (silent mating type information regulation 2 homolog-2) mitigates damaging of hypertensive nephropathy. Circ Res. 2023;132:601–24. 10.1161/CIRCRESAHA.122.321591. PMC9977266 36786216

[182] Yang S, Yang G, Wang X, Xiang J, Kang L, Liang Z. SIRT2 alleviated renal fibrosis by deacetylating SMAD2 and SMAD3 in renal tubular epithelial cells. Cell Death Dis. 2023;14:646. 10.1038/s41419-023-06169-1. PMC10542381 37777567

[183] Wei T, Gao J, Huang C, Song B, Sun M, Shen W. SIRT3 (Sirtuin-3) Prevents Ang II (Angiotensin II)-Induced Macrophage Metabolic Switch Improving Perivascular Adipose Tissue Function. Arterioscler Thromb Vasc Biol. 2021;41:714–730. 10.1161/ATVBAHA.120.315337. 33327751

[184] Li X, Liu J, Lu L, Huang T, Hou W, Wang F, et al. Sirt7 associates with ELK1 to participate in hyperglycemia memory and diabetic nephropathy via modulation of DAPK3 expression and endothelial inflammation. Transl Res. 2022;247:99–116. 10.1016/j.trsl.2022.04.005. 35470010

[185] Yao K, Mou Q, Lou X, Ye M, Zhao B, Hu Y, et al. Microglial SIRT1 activation attenuates synapse loss in retinal inner plexiform layer via mTORC1 inhibition. J Neuroinflammation. 2023;20:202. 10.1186/s12974-023-02886-8. PMC10481494 37670386

[186] Bordone L, Motta MC, Picard F, Robinson A, Jhala US, Apfeld J, et al. Sirt1 regulates insulin secretion by repressing UCP2 in pancreatic beta cells. PLoS Biol. 2006;4:e31. 10.1371/journal.pbio.0040031. PMC1318478 16366736

[187] Zhu Q, Wang W, Wu N, He S, Lin X, Zhang W, et al. 7-Ketocholesterol accelerates pancreatic β-cell senescence by inhibiting the SIRT1/CDK4-Rb-E2F1 signaling pathway. Islets. 2023;15:2219105. 10.1080/19382014.2023.2219105. PMC10240968 37265106

[188] Chen J, Lou R, Zhou F, Li D, Peng C, Lin L. Sirtuins: Key players in obesity-associated adipose tissue remodeling. Front Immunol. 2022;13:1068986. 10.3389/fimmu.2022.1068986. PMC9730827 36505468

[189] Bae EJ, Park BH. Multiple Roles of Sirtuin 6 in Adipose Tissue Inflammation. Diabetes Metab J. 2023;47:164–72. 10.4093/dmj.2022.0270. PMC10040615 36631993

[190] Zhou Q, Lu Z, Wang B, Wang Y, Li L, You M, et al. Endothelial SIRT3 deficiency predisposes brown adipose tissue to whitening in diet-induced obesity. Int J Biol Sci. 2025;21:3444–60. 10.7150/ijbs.110741. PMC12160546 40520025

[191] Zhao Y, Li J, Ma A, Wang Z, Ni Y, Wu D, et al. Irisin alleviates hepatic steatosis by activating the autophagic SIRT3 pathway. Chin Med J (Engl). 2026;139:443–56. 10.1097/CM9.0000000000003427. PMC12875703 39965865

[192] Chen X, Wang Z, Li C, Zhang Z, Lu S, Wang X, et al. SIRT1 activated by AROS sensitizes glioma cells to ferroptosis via induction of NAD+ depletion-dependent activation of ATF3. Redox Biol. 2024;69:103030. 10.1016/j.redox.2024.103030. PMC10791567 38181705

[193] Tsukihara S, Akiyama Y, Shimada S, Hatano M, Igarashi Y, Taniai T, et al. Delactylase effects of SIRT1 on a positive feedback loop involving the H19-glycolysis-histone lactylation in gastric cancer. Oncogene. 2025;44:724–38. 10.1038/s41388-024-03243-6. 39658647

[194] Xu H, Zeng S, Wang Y, Yang T, Wang M, Li X, et al. Cytoplasmic SIRT1 promotes paclitaxel resistance in ovarian carcinoma through increased formation and survival of polyploid giant cancer cells. J Pathol. 2023;261:210–26. 10.1002/path.6167. 37565313

[195] Wang X, Jiang Y, Ye W, Shao C, Xie J, Li X. SIRT1 promotes the progression and chemoresistance of colorectal cancer through the p53/miR-101/KPNA3 axis. Cancer Biol Ther. 2023;24:2235770. 10.1080/15384047.2023.2235770. PMC10431729 37575080

[196] Wu G, Yao W, Cheng L, Wang X, Chen T. SIRT1 rescues autophagic flux via PI3K/AKT/mTOR inactivation to suppress DOX-induced senescence in MCF-7 cells. Exp Cell Res. 2025;452:114754. 10.1016/j.yexcr.2025.114754. 40962168

[197] Yi F, Zhang Y, Wang Z, Wang Z, Li Z, Zhou T, et al. The deacetylation-phosphorylation regulation of SIRT2-SMC1A axis as a mechanism of antimitotic catastrophe in early tumorigenesis. Sci Adv. 2021;7:eabe5518. 10.1126/sciadv.abe5518. PMC7904255 33627431

[198] Tian Y, Liu R, Hou X, Gao Z, Liu X, Zhang W. SIRT2 promotes the viability, invasion and metastasis of osteosarcoma cells by inhibiting the degradation of Snail. Cell Death Dis. 2022;13:935. 10.1038/s41419-022-05388-2. PMC9640536 36344502

[199] Geng A, Sun J, Tang H, Yu Y, Wang X, Zhang J, et al. SIRT2 promotes base excision repair by transcriptionally activating OGG1 in an ATM/ATR-dependent manner. Nucleic Acids Res. 2024;52:5107–20. 10.1093/nar/gkae190. PMC11109957 38554113

[200] Zhu H, Hu Y, Zeng C, Chang L, Ge F, Wang W, et al. The SIRT2-mediated deacetylation of AKR1C1 is required for suppressing its pro-metastasis function in Non-Small Cell Lung Cancer. Theranostics. 2020;10:2188–200. 10.7150/thno.39151. PMC7019158 32104503

[201] Zhang X, Xu Y, Li S, Qin Y, Zhu G, Zhang Q, et al. SIRT2-mediated deacetylation of ACLY promotes the progression of oesophageal squamous cell carcinoma. J Cell Mol Med. 2024;28:e18129. 10.1111/jcmm.18129. PMC10906381 38426936

[202] Wang B, Ye Y, Yang X, Liu B, Wang Z, Chen S, et al. SIRT2-dependent IDH1 deacetylation inhibits colorectal cancer and liver metastases. EMBO Rep. 2020;21:e48183. 10.15252/embr.201948183. PMC7132198 32141187

[203] Geng X, Li M, Zhang L, Cai Y, Chen X, Mu X, et al. P5CS deacetylation mediated by SIRT2 facilitates tumor growth by enhancing mitochondrial respiration in hepatocellular carcinoma. Oncogene. 2025;44:2746–61. 10.1038/s41388-025-03456-3. 40425834

[204] Zhang X, Gao Y, Zhang Y, Zhang Y, Chen Y, Pei X, et al. SIRT5-mediated desuccinylation of MTHFD2 enhances chemoresistance in breast cancer cells by reducing therapy-induced senescence. Commun Biol. 2025;8:1485. 10.1038/s42003-025-08878-z. PMC12537947 41116031

[205] Deng M, Fang Y, Chu Y, Ding R, Fan X, Wei H, et al. SIRT3 is required for the protective function of ketogenic diet on neural inflammation and neuropathic pain. Int J Biol Sci. 2025;21:3011–29. 10.7150/ijbs.110921. PMC12080379 40384870

[206] Pan JP, Zhang J, Wang PJ, Chen G, Fang EF. Mitophagy mitigates tau acetylation via the ULK1-NAD+/SIRT1 axis in Alzheimer's disease. Autophagy. 2026;1–3. 10.1080/15548627.2026.2689031. 42323822

[207] Lu S, Yin X, Wang J, Gu Q, Huang Q, Jin N, et al. SIRT1 regulates O-GlcNAcylation of tau through OGT. Aging (Albany NY). 2020;12:7042–55. 10.18632/aging.103062. PMC7202539 32310828

[208] Qian S, Gu J, Dai W, Jin N, Chu D, Huang Q, et al. Sirt1 enhances tau exon 10 inclusion and improves spatial memory of Htau mice. Aging (Albany NY). 2018;10:2498–510. 10.18632/aging.101564. PMC6188499 30243024

[209] Shen Y, Wang X, Nan N, Fu X, Zeng R, Yang Y, et al. SIRT3-Mediated Deacetylation of SDHA Rescues Mitochondrial Bioenergetics Contributing to Neuroprotection in Rotenone-Induced PD Models. Mol Neurobiol. 2024;61:4402–20. 10.1007/s12035-023-03830-w. 38087172

[210] Smirnov D, Eremenko E, Stein D, Kaluski S, Jasinska W, Cosentino C, et al. SIRT6 is a key regulator of mitochondrial function in the brain. Cell Death Dis. 2023;14:35. 10.1038/s41419-022-05542-w. PMC9849342 36653345

[211] Hou Y, Lautrup S, Cordonnier S, Wang Y, Croteau DL, Zavala E, et al. NAD+ supplementation normalizes key Alzheimer’s features and DNA damage responses in a new AD mouse model with introduced DNA repair deficiency. Proc Natl Acad Sci U S A. 2018;115:E1876–85. 10.1073/pnas.1718819115. PMC5828618 29432159

[212] Kumari S, Singh H, Vaidya S, Taliyan R. Targeting Sirtuin Signaling in Parkinson's Disease and Neurodegeneration: Molecular Insights and Translational Potential. Mol Neurobiol. 2026;63(1):775. 10.1007/s12035-026-06062-w. 42467143

[213] Yang K, Velagapudi S, Akhmedov A, Kraler S, Lapikova-Bryhinska T, Schmiady MO, et al. Chronic SIRT1 supplementation in diabetic mice improves endothelial function by suppressing oxidative stress. Cardiovasc Res. 2023;119:2190–201. 10.1093/cvr/cvad102. PMC10578911 37401647

[214] Ding YN, Wang HY, Chen XF, Tang X, Chen HZ. Roles of Sirtuins in Cardiovascular Diseases: Mechanisms and Therapeutics. Circ Res. 2025;136:524–50. 10.1161/CIRCRESAHA.124.325440. 40014680

[215] Pillai VB, Samant S, Hund S, Gupta M, Gupta MP. The nuclear sirtuin SIRT6 protects the heart from developing aging-associated myocyte senescence and cardiac hypertrophy. Aging (Albany NY). 2021;13:12334–58. 10.18632/aging.203027. PMC8148452 33934090

[216] Araki S, Izumiya Y, Rokutanda T, Ianni A, Hanatani S, Kimura Y, et al. Sirt7 Contributes to Myocardial Tissue Repair by Maintaining Transforming Growth Factor-β Signaling Pathway. Circulation. 2015;132:1081–93. 10.1161/CIRCULATIONAHA.114.014821. 26202810

[217] Chen YF, Qi RQ, Song JW, Wang SY, Dong ZJ, Chen YH, et al. Sirtuin 7 ameliorates cuproptosis, myocardial remodeling and heart dysfunction in hypertension through the modulation of YAP/ATP7A signaling. Apoptosis. 2024;29:2161–82. 10.1007/s10495-024-02021-9. 39394530

[218] Liu S, Deshmukh V, Meng F, Wang Y, Morikawa Y, Steimle JD, et al. Microtubules Sequester Acetylated YAP in the Cytoplasm and Inhibit Heart Regeneration. Circulation. 2025;151:59–75. 10.1161/CIRCULATIONAHA.123.067646. PMC11671299 39185559

[219] Peng F, Liao M, Jin W, Liu W, Li Z, Fan Z, et al. 2-APQC, a small-molecule activator of Sirtuin-3 (SIRT3), alleviates myocardial hypertrophy and fibrosis by regulating mitochondrial homeostasis. Signal Transduct Target Ther. 2024;9:133. 10.1038/s41392-024-01816-1. PMC11094072 38744811

[220] Pillai VB, Sundaresan NR, Jeevanandam V, Gupta MP. Mitochondrial SIRT3 and heart disease. Cardiovasc Res. 2010;88:250–6. 10.1093/cvr/cvq250. PMC2952535 20685942

[221] Jheng JR, Bai Y, Noda K, Huot JR, Cook T, Fisher A, et al. Skeletal Muscle SIRT3 Deficiency Contributes to Pulmonary Vascular Remodeling in Pulmonary Hypertension Due to Heart Failure With Preserved Ejection Fraction. Circulation. 2024;150:867–83. 10.1161/CIRCULATIONAHA.124.068624. PMC11384544 38804138

[222] Grzeczka A, Graczyk S, Gong X, Gröschel J, Spethmann S, Kordowitzki P. Aging hearts, fibrotic fears: The sirtuin connection. Biomed Pharmacother. 2025;193:118882. 10.1016/j.biopha.2025.118882. 41349250

[223] Zhang Y, Wang X, Li XK, Lv SJ, Wang HP, Liu Y, et al. Sirtuin 2 deficiency aggravates ageing-induced vascular remodelling in humans and mice. Eur Heart J. 2023;44:2746–59. 10.1093/eurheartj/ehad381. PMC10393077 37377116

[224] Xue T, Wang S, Zhu X, Luo J, Zhu R, Li H, et al. Protective mechanisms of Sirtuin Family in myocardial ischemia-reperfusion injury and translational therapeutic perspectives. Mol Biol Rep. 2026;53(1):1389. 10.1007/s11033-026-12521-3. 42584557

[225] Wu M, Tan J, Zhou W, Zhang Z, Gao Q, Cai Y, et al. SIRT5 Ameliorates Cardiac Fibrosis via PCK2 Desuccinylation-Mediated Metabolic Reprogramming in Cardiac Fibroblasts. Circulation. 2026;154:243–64. 10.1161/CIRCULATIONAHA.125.077756. 42021758

[226] López-Otín C, Blasco MA, Partridge L, Serrano M, Kroemer G. The hallmarks of aging. Cell. 2013;153:1194–217. 10.1016/j.cell.2013.05.039. PMC3836174 23746838

[227] López-Otín C, Blasco MA, Partridge L, Serrano M, Kroemer G. Hallmarks of aging: An expanding universe. Cell. 2023;186:243–78. 10.1016/j.cell.2022.11.001. 36599349

[228] Zhang ZN, Chung SK, Xu Z, Xu Y. Oct4 maintains the pluripotency of human embryonic stem cells by inactivating p53 through Sirt1-mediated deacetylation. Stem Cells. 2014;32:157–65. 10.1002/stem.1532. PMC3947311 24038750

[229] Wang H, Diao D, Shi Z, Zhu X, Gao Y, Gao S, et al. SIRT6 Controls Hematopoietic Stem Cell Homeostasis through Epigenetic Regulation of Wnt Signaling. Cell Stem Cell. 2016;18:495–507. 10.1016/j.stem.2016.03.005. 27058938

[230] Zhai XY, Yan P, Zhang J, Song HF, Yin WJ, Gong H, et al. Knockdown of SIRT6 Enables Human Bone Marrow Mesenchymal Stem Cell Senescence. Rejuvenation Res. 2016;19:373–84. 10.1089/rej.2015.1770. 26654351

[231] Paredes S, Angulo-Ibanez M, Tasselli L, Carlson SM, Zheng W, Li TM, et al. The epigenetic regulator SIRT7 guards against mammalian cellular senescence induced by ribosomal DNA instability. J Biol Chem. 2018;293:11242–50. 10.1074/jbc.AC118.003325. PMC6052228 29728458

[232] Lagunas-Rangel FA. SIRT7 in the aging process. Cell Mol Life Sci. 2022;79:297. 10.1007/s00018-022-04342-x. PMC9117384 35585284

[233] Correia M, Perestrelo T, Rodrigues AS, Ribeiro MF, Pereira SL, Sousa MI, et al. Sirtuins in metabolism, stemness and differentiation. Biochim Biophys Acta Gen Subj. 2017;1861:3444–55. 10.1016/j.bbagen.2016.09.008. 27614149

[234] Katsyuba E, Romani M, Hofer D, Auwerx J. NAD+ homeostasis in health and disease. Nat Metab. 2020;2:9–31. 10.1038/s42255-019-0161-5. 32694684

[235] Camacho-Pereira J, Tarragó MG, Chini C, Nin V, Escande C, Warner GM, et al. CD38 Dictates Age-Related NAD Decline and Mitochondrial Dysfunction through an SIRT3-Dependent Mechanism. Cell Metab. 2016;23:1127–39. 10.1016/j.cmet.2016.05.006. PMC4911708 27304511

[236] Rajman L, Chwalek K, Sinclair DA. Therapeutic Potential of NAD-Boosting Molecules: The In Vivo Evidence. Cell Metab. 2018;27:529–47. 10.1016/j.cmet.2018.02.011. PMC6342515 29514064

[237] Imai S, Guarente L. NAD+ and sirtuins in aging and disease. Trends Cell Biol. 2014;24:464–71. 10.1016/j.tcb.2014.04.002. PMC4112140 24786309

[238] Zgutka K, Mikołajek-Bedner W, Szumilas K, Tarnowski M. Sirtuins as Molecular Mediators of Caloric Restriction in the Pancreas: Implications for β-Cell Function, Metabolism, and Longevity. Int J Mol Sci. 2026;27:6922. 10.3390/ijms27156922. PMC13467452 42589577

[239] You Y, Liang W. SIRT1 and SIRT6: the role in aging-related diseases. Biochimica et Biophysica Acta (BBA). 2023;1869:166815. 10.1016/j.bbadis.2023.166815. 37499928

[240] Liu Y, Zhang Z, Wang T, Yang X, Zhang Q, Liu X, et al. Hepatic SIRT6 Deficiency Accelerates Female-Specific Aging Through SULT1E1-Mediated Estrogen Depletion. Int J Mol Sci. 2026;27:7039. 10.3390/ijms27157039. PMC13467427 42589691

[241] Qu Q, Chen Y, Wang Y, Wang W, Long S, Yang HY, et al. Lithocholic acid binds TULP3 to activate sirtuins and AMPK to slow down ageing. Nature. 2025;643:201–9. 10.1038/s41586-024-08348-2. PMC12222023 39695235

[242] Patel KR, Scott E, Brown VA, Gescher AJ, Steward WP, Brown K. Clinical trials of resveratrol. Ann N Y Acad Sci. 2011;1215:161–9. 10.1111/j.1749-6632.2010.05853.x. 21261655

[243] Lagouge M, Argmann C, Gerhart-Hines Z, Meziane H, Lerin C, Daussin F, et al. Resveratrol improves mitochondrial function and protects against metabolic disease by activating SIRT1 and PGC-1alpha. Cell. 2006;127:1109–22. 10.1016/j.cell.2006.11.013. 17112576

[244] Price NL, Gomes AP, Ling AJY, Duarte FV, Martin-Montalvo A, North BJ, et al. SIRT1 is required for AMPK activation and the beneficial effects of resveratrol on mitochondrial function. Cell Metab. 2012;15:675–90. 10.1016/j.cmet.2012.04.003. PMC3545644 22560220

[245] Crowell JA, Korytko PJ, Morrissey RL, Booth TD, Levine BS. Resveratrol-associated renal toxicity. Toxicol Sci. 2004;82:614–9. 10.1093/toxsci/kfh263. 15329443

[246] Chao C, Huang C, Cheng J, Chiou C, Lee I, Yang Y, et al. SRT1720 as an SIRT1 activator for alleviating paraquat-induced models of Parkinson’s disease. Redox Biol. 2022;58:102534. 10.1016/j.redox.2022.102534. PMC9663539 36379180

[247] Hubbard BP, Gomes AP, Dai H, Li J, Case AW, Considine T, et al. Evidence for a common mechanism of SIRT1 regulation by allosteric activators. Science. 2013;339:1216–9. 10.1126/science.1231097. PMC3799917 23471411

[248] Outeiro TF, Kontopoulos E, Altmann SM, Kufareva I, Strathearn KE, Amore AM, et al. Sirtuin 2 inhibitors rescue alpha-synuclein-mediated toxicity in models of Parkinson’s disease. Science. 2007;317:516–9. 10.1126/science.1143780. 17588900

[249] Lin C, Zhou X, Li M, Zhang C, Zhai H, Li H, et al. S-ketamine Alleviates Neuroinflammation and Attenuates Lipopolysaccharide-Induced Depression Via Targeting SIRT2. Adv Sci (Weinh). 2025;12:e2416481. 10.1002/advs.202416481. PMC12199433 40171943

[250] Mao A, Fu Y, Zhang M, Wu J, Wu M. Phenotypic screening identified polydatin alleviating cartilage degeneration by modulating SIRT3-dependent mitochondrial dysfunction. Phytomedicine. 2025;144:156948. 10.1016/j.phymed.2025.156948. 40505483

[251] Deng Z, He M, Hu H, Zhang W, Zhang Y, Ge Y, et al. Melatonin attenuates sepsis-induced acute kidney injury by promoting mitophagy through SIRT3-mediated TFAM deacetylation. Autophagy. 2024;20:151–65. 10.1080/15548627.2023.2252265. PMC10761103 37651673

[252] Zhang D, Zhang J, Wu C, Xiao Y, Ji L, Hu J, et al. Unraveling Small Molecule-Mediated Sirtuin 3 Activation at a Distinct Binding Site for Cardioprotective Therapies. ACS Cent Sci. 2025;11:704–18. 10.1021/acscentsci.5c00023. PMC12123458 40454347

[253] Deng J, Liu Z, Zhu K, Cui G, Liu L, Yan Y, et al. New ε-N-thioglutaryl-lysine derivatives as SIRT5 inhibitors: Chemical synthesis, kinetic and crystallographic studies. Bioorg Chem. 2023;135:106487. 10.1016/j.bioorg.2023.106487. 36996510

[254] Lulu TB, Manikandan DB, Pevzner Y, Sklarz M, Eisdorfer SA, Awad S, et al. Pharmacological activation of SIRT6 suppresses progression of head and neck and esophageal squamous cell carcinoma by modulation of cellular metabolism and protein translation. Cell Death Dis. 2025;16:727. 10.1038/s41419-025-07959-5. PMC12533074 41102169

[255] He T, Shang J, Gao C, Guan X, Chen Y, Zhu L, et al. A novel SIRT6 activator ameliorates neuroinflammation and ischemic brain injury via EZH2/FOXC1 axis. Acta Pharm Sin B. 2021;11:708–26. 10.1016/j.apsb.2020.11.002. PMC7982432 33777677

[256] Daveson A, Stubbs R, Polasek TM, Isola J, Anderson R, Tye-Din JA, et al. Safety, clinical activity, pharmacodynamics, and pharmacokinetics of IMU-856, a SIRT6 modulator, in coeliac disease: a first-in-human, randomised, double-blind, placebo-controlled, phase 1 trial. Lancet Gastroenterol Hepatol. 2025;10:44–54. 10.1016/S2468-1253(24)00248-6. 39521016

[257] Li S, Wu B, Zheng W. Cyclic tripeptide-based potent human SIRT7 inhibitors. Bioorg Med Chem Lett. 2019;29:461–5. 10.1016/j.bmcl.2018.12.023. 30578034

[258] Wang J, Tian H, Gao Y, Qiu X, Bao Z, Zhao D, et al. Dual Roles of SIRT7 Inhibition by Oroxylin A Reprogram HSCs Fate: PRMT5 Succinylation-Driven Senescence and Ecto-Calreticulin-Dependent NK Cell Immune Clearance in Liver Fibrosis. Research (Wash D C). 2025;8:0808. 10.34133/research.0808. PMC12329212 40777599

[259] Zhou F, Zhang L, Zhu K, Bai M, Zhang Y, Zhu Q, et al. SIRT2 ablation inhibits glucose-stimulated insulin secretion through decreasing glycolytic flux. Theranostics. 2021;11:4825–38. 10.7150/thno.55330. PMC7978320 33754030

[260] Chopra V, Quinti L, Kim J, Vollor L, Narayanan KL, Edgerly C, et al. The sirtuin 2 inhibitor AK-7 is neuroprotective in Huntington’s disease mouse models. Cell Rep. 2012;2:1492–7. 10.1016/j.celrep.2012.11.001. PMC3534897 23200855

[261] Pillai VB, Samant S, Sundaresan NR, Raghuraman H, Kim G, Bonner MY, et al. Honokiol blocks and reverses cardiac hypertrophy in mice by activating mitochondrial Sirt3. Nat Commun. 2015;6:6656. 10.1038/ncomms7656. PMC4441304 25871545

[262] Zhang C, Li Y, Liu B, Ning C, Li Y, Wang Y, et al. Discovery of SIRT7 Inhibitor as New Therapeutic Options Against Liver Cancer. Front Cell Dev Biol. 2021;9:813233. 10.3389/fcell.2021.813233. PMC8841758 35174171

[263] Schiedel M, Herp D, Hammelmann S, Swyter S, Lehotzky A, Robaa D, et al. Chemically Induced Degradation of Sirtuin 2 (Sirt2) by a Proteolysis Targeting Chimera (PROTAC) Based on Sirtuin Rearranging Ligands (SirReals). J Med Chem. 2018;61:482–91. 10.1021/acs.jmedchem.6b01872. 28379698

[264] Schiedel M, Lehotzky A, Szunyogh S, Oláh J, Hammelmann S, Wössner N, et al. HaloTag-Targeted Sirtuin-Rearranging Ligand (SirReal) for the Development of Proteolysis-Targeting Chimeras (PROTACs) against the Lysine Deacetylase Sirtuin 2 (Sirt2)*. ChemBioChem. 2020;21:3371–6. 10.1002/cbic.202000351. PMC7754454 32672888

[265] Szabó A, Oláh J, Szunyogh S, Lehotzky A, Szénási T, Csaplár M, et al. Modulation Of Microtubule Acetylation By The Interplay Of TPPP/p25, SIRT2 And New Anticancer Agents With Anti-SIRT2 Potency. Sci Rep. 2017;7:17070. 10.1038/s41598-017-17381-3. PMC5719079 29213065

[266] Huang J, Su J, Wang H, Chen J, Tian Y, Zhang J, et al. Discovery of Novel PROTAC SIRT6 Degraders with Potent Efficacy against Hepatocellular Carcinoma. J Med Chem. 2024;67:17319–49. 10.1021/acs.jmedchem.4c01223. 39323022

[267] Zheng K, Sha N, Hou G, Leng Z, Zhao Q, Zhang L, et al. IGF1R-phosphorylated PYCR1 facilitates ELK4 transcriptional activity and sustains tumor growth under hypoxia. Nat Commun. 2023;14:6117. 10.1038/s41467-023-41658-z. PMC10542766 37777542

[268] Xu S, Tao H, Cao W, Cao L, Lin Y, Zhao S, et al. Ketogenic diets inhibit mitochondrial biogenesis and induce cardiac fibrosis. Signal Transduct Target Ther. 2021;6:54. 10.1038/s41392-020-00411-4. PMC7870678 33558457

[269] Cheng Y, Zheng G, Huang H, Ni J, Zhao Y, Sun Y, et al. GLSP mitigates vascular aging by promoting Sirt7-mediated Keap1 deacetylation and Keap1-Nrf2 dissociation. Theranostics. 2025;15:4345–67. 10.7150/thno.110324. PMC11984382 40225574

[270] Pulipaka S, Singuru G, Sahoo S, Shaikh A, Thennati R, Kotamraju S. Therapeutic efficacies of mitochondria-targeted esculetin and metformin in the improvement of age-associated atherosclerosis via regulating AMPK activation. Geroscience. 2024;46:2391–408. 10.1007/s11357-023-01015-w. PMC10828355 37968424

[271] Chen J, Liu B, Yao X, Yang X, Sun J, Yi J, et al. AMPK/SIRT1/PGC-1α Signaling Pathway: Molecular Mechanisms and Targeted Strategies From Energy Homeostasis Regulation to Disease Therapy. CNS Neurosci Ther. 2025;31:e70657. 10.1111/cns.70657. PMC12635776 41268687

[272] Zhang J, Xu C, Tang X, Sun S, Liu S, Yang L, et al. Endothelium-specific SIRT7 targeting ameliorates pulmonary hypertension through Krüpple-like factor 4 deacetylation. Cardiovasc Res. 2024;120:403–16. 10.1093/cvr/cvae011. PMC10981524 38198357

[273] Wang D, Wu Y, Zhao Y, Ma X, Shi J, Ni J, et al. The novel SIRT2-targeted PROTAC degraders as the efficient agents for the treatment of ovarian cancer. Eur J Med Chem. 2026;302:118295. 10.1016/j.ejmech.2025.118295. 41166762

[274] Itoh Y. Chemical protein degradation approach and its application to epigenetic targets. Chem Rec. 2018;18:1681–700. 10.1002/tcr.201800032. 29893461

[275] Chen S, Zheng Y, Liang B, Yin Y, Yao J, Wang Q, et al. The application of PROTAC in HDAC. Eur J Med Chem. 2023;260:115746. 10.1016/j.ejmech.2023.115746. 37607440

[276] Sharma C, Donu D, Curry AM, Barton E, Cen Y. Multifunctional activity-based chemical probes for sirtuins. RSC Adv. 2023;13:11771–81. 10.1039/d3ra02133e. PMC10103746 37063743

[277] Aly A, Tan Z, Sasidharan S, Oon CE. Decoding context-dependent sirtuin pharmacology in cancer: Metabolic-epigenetic switches and precision therapeutic targeting. Crit Rev Oncol Hematol. 2026;226:105513. 10.1016/j.critrevonc.2026.105513. 42543141

